# Spinal instrumentation length affects adjacent segment range of motion and intradiscal pressure

**DOI:** 10.1038/s41598-024-82132-0

**Published:** 2024-12-16

**Authors:** Christian Liebsch, Peter Obid, Morten Vogt, Benedikt Schlager, Hans-Joachim Wilke

**Affiliations:** 1 Institute of Orthopaedic Research and Biomechanics, Trauma Research Centre Ulm, Ulm University Medical Centre, Helmholtzstraße 14, 89081 Ulm, Germany; 2 Department of Orthopaedics and Trauma Surgery, Freiburg University Medical Centre, Freiburg, Germany

**Keywords:** Scoliosis, Spine, Range of motion, Intradiscal pressure, In vitro study, Biomechanics, Translational research, Paediatric research, Biomedical engineering

## Abstract

Scoliosis instrumentation length depends on the type and degree of deformity and the individual preference of the surgeon. This in vitro study aimed to explore effects of increasing instrumentation length on adjacent segment mobility and intervertebral disc loading. Six fresh frozen human spine specimens (C7-sacrum) with entire rib cage from young adult donors (26–45 years) were loaded with pure moments of 5 Nm. Range of motion (ROM) of all segments was determined using optical motion tracking. Lumbar intradiscal pressure (IDP) was measured using flexible pressure sensors from L1 to L5. The specimens were tested in two groups with increasing posterior instrumentation length in proximal (group 1) and distal direction (group 2). Significant (*p* < 0.05) adjacent segment ROM increases compared to the condition without any instrumentation and compared to other instrumentations were primarily found proximally to the instrumentation in lateral bending. IDP significantly (*p* < 0.05) increased in flexion in the distal adjacent segment for T4-L1 instrumentation and by up to 550% at instrumented levels compared to the condition without instrumentation. These findings may explain clinical complications such as adjacent segment disease and associated proximal and distal junctional kyphosis. To reduce loads on adjacent segments, instrumentation should therefore be applied as short as reasonable.

## Introduction

Surgical treatment of scoliosis comprises a wide variety of available techniques and instrumentation types. One of the most relevant challenges regarding surgical strategies represents the selection of upper (UIV) and lower (LIV) instrumented vertebrae. Although widely accepted recommendations for instrumentation levels in scoliosis surgery were published^1,2^, multiple surveys among experienced scoliosis surgeons revealed large discrepancies regarding the chosen instrumentation levels for specific curve types^3–6^. This variability could be caused by different factors, of which one might be the lack of experimental studies on the effect of the instrumentation length on spinal flexibility and loading^5^. Previous in vivo studies reported overall range of motion decrease without compensatory range of motion increase above and below the instrumentation^7–10^ and even reduced lumbar spinal range of motion in flexion^10–13^, lateral bending^12–14^, and axial rotation^12^ with a more distal LIV. One in vivo study, however, specifically investigating the adjacent level below instrumentation, detected higher lumbar spinal adjacent segment range of motion in lateral bending with a more distal LIV and concluded that preservation of motion segments better distributes functional mobility across the uninstrumented segmental levels, potentially reducing the risk of adjacent segment overstress^15^. With regard to commonly observed long-term complications following scoliosis surgery, such as adjacent segment degeneration^16,17^ and associated proximal^18,19^ and distal junctional kyphosis^20,21^, exhibiting prevalences of about 64%^16^, 26%^18^, and 14–19%^20,21^, respectively, better knowledge about potential spinal compensatory mechanisms and loading alterations due to instrumentation is essential in order to optimize instrumentation strategies on a biomechanical basis. This in vitro study therefore aimed to investigate the effects of different instrumentation lengths on the segmental range of motion of the entire thoracic and lumbar spine as well as on the intradiscal pressure of the lumbar spine under standardized conditions.

## Methods

**Fig. 1:**
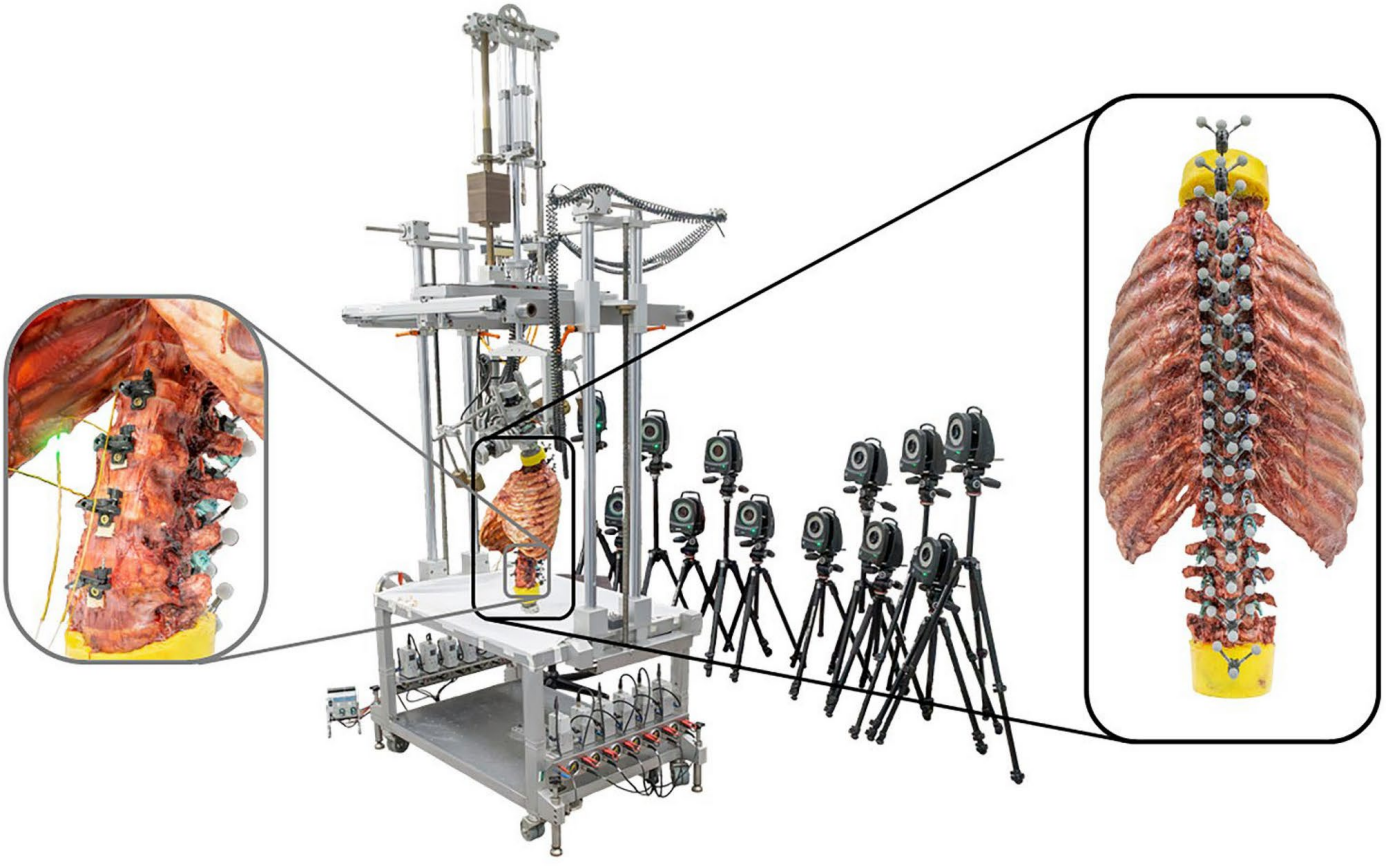
Overview of the experimental setup including the spine tester together with the optical motion tracking system consisting of twelve infrared cameras (middle) and three retroreflective markers per vertebra from C7 to the sacrum for segmental flexibility measurement (right), as well as the flexible intradiscal pressure sensors at levels L1-L2 to L4-L5 (left).

Six fresh frozen human thoracolumbar spine specimens (C7-sacrum) with complete rib cages were tested in this in vitro study. The specimens originated from young donors (26–45 years, 2 female/4 male) and were acquired from an accredited body donation program (Science Care Inc., Phoenix, Arizona, USA) after approval of the ethics committee of the University of Ulm for the use of human specimens (no. 63/17). The specimens showed no signs of clinically relevant deformity (coronal Cobb angles < 10°, sagittal Cobb angles < 40° for thoracic and < 60° for lumbar spine), degeneration, or injury and exhibited adequate bone quality (BMD 80–164 mg/cc HA), which was confirmed by CT scans (Siemens Somatom Definition AS, Siemens Healthcare, Erlangen, Germany) prior to testing. The specimens were stored at − 20 °C and thawed for about 12 h at 5 °C before preparation and testing^22^. During preparation, any muscle, fat, and nerve tissues except for the intercostal muscles, which were shown to stabilize the thoracic spine^23^, were carefully removed and the upper- (C7) and lowermost (sacrum) levels were half embedded in polymethylmethacrylate (PMMA, Technovit 3040, Heraeus Kulzer, Wehrheim, Germany), leaving intact any bone, ligament, and cartilage structures. During experimental testing, the specimens were kept moist with 0.9% saline solution. The specimens were loaded quasi-statically and displacement-controlled with pure moments of 5 Nm in flexion/extension, lateral bending, and axial rotation^22^ using a well-established spine tester^24^. Displacement was applied continuously with an angular velocity of 1°/s for 3.5 load cycles, of which the third full load cycle was chosen for data evaluation to minimize viscoelastic effects^22^. Simultaneously with spinal loading, optical motion tracking of each spinal level (C7-sacrum) was performed using three combined retroreflective markers per level, which were rigidly fixed via screwing into the spinous processes, and twelve free-standing infrared cameras (Vicon MX13+, Vicon Motion Systems Ltd., Oxford, UK) (Fig. 1), which represents a method that has already been successfully applied in previous in vitro studies on thoracic spine and rib cage specimens^23,25–29^. Additionally, intradiscal pressure measurements were performed at L1-L2, L2-L3, L3-L4, L4-L5 levels using flexible pressure sensors (FOP-LS-2FR-30, FISO Technologies Inc., Québec, Canada). Sensors were implanted via cannulas and rigidly fixed to the respective lower vertebra using screws to avoid pull-out. Prior to testing, central position of the sensor tip within the interverbal disc was confirmed by plane radiography (X-ray source: Mobil TT XD, Model 01818363, Siemens, Erlangen, Germany; film developer: FCR Prima CR IR 391 RU, Fujifilm Holdings, Tokyo, Japan).

**Fig. 2:**
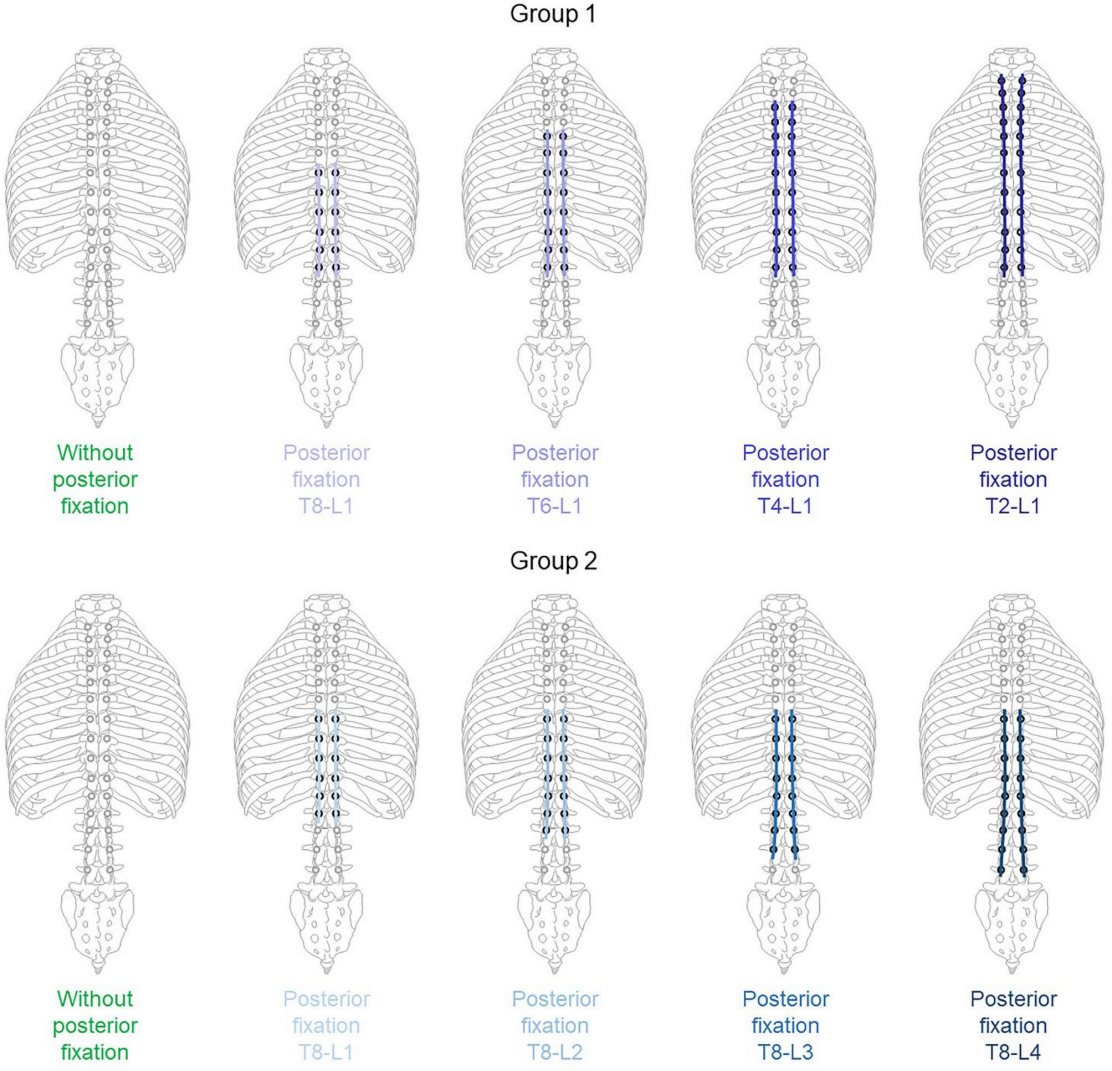
Overview of testing steps for both study groups (n = 6 per condition).

The specimens were first tested with solely polyaxial pedicle screws implanted from T2 to L4 levels, serving as reference condition, followed by seven conditions with different rod configurations (T2-L1, T4-L1, T6-L1, T8-L1, T8-L4, T8-L3, T8-L2) resulting in two groups with four increasing instrumentation lengths in proximal (group 1) and distal (group 2) directions with a sample size of n = 6 per group, respectively (Fig. 2). The two conditions without any instrumentation and with instrumentation from T8 to L1 were included in both groups to provide a sufficient number of clinically relevant conditions within the two groups. Screw sizes were chosen individually for each pedicle according to pedicle size and vertebra length, ranging from 4.5 × 30 mm to 4.5 × 40 mm at T2 level and from 6.5 × 50 mm to 7.5 × 55 mm at L4 level (Ennovate®, Aesculap AG, Tuttlingen, Germany). As connectors, two titanium rods with a diameter of 5.5 mm were used per group, which were cut in situ prior to each following test condition using a bolt cutter in order to ensure direct comparability between the single testing steps within each group. Rod configurations were based on results of two surveys among spinal deformity surgeons^3,4^ regarding their preferred surgical treatment of Lenke types 2 (double thoracic curve, group 1) and 5 (thoraco-/lumbar curve, group 2) of adolescent idiopathic scoliosis^30^. Screw implantation and fitting of the rods to the spinal curvature were performed by an experienced spine surgeon, making sure to maintain the natural curvature.

Post-processing of data was performed using Excel2019 (Microsoft Excel, Microsoft Corp., Redmond, USA). Statistical differences between the single testing steps within each group were evaluated using Friedman’s ANOVA with Bonferroni-Dunn post-hoc correction and pairwise comparisons as well as additional pairwise Friedman test in SPSS27 (IBM Corp., Armonk, USA), with the significance level being set at 0.05.

## Results

**Fig. 3:**
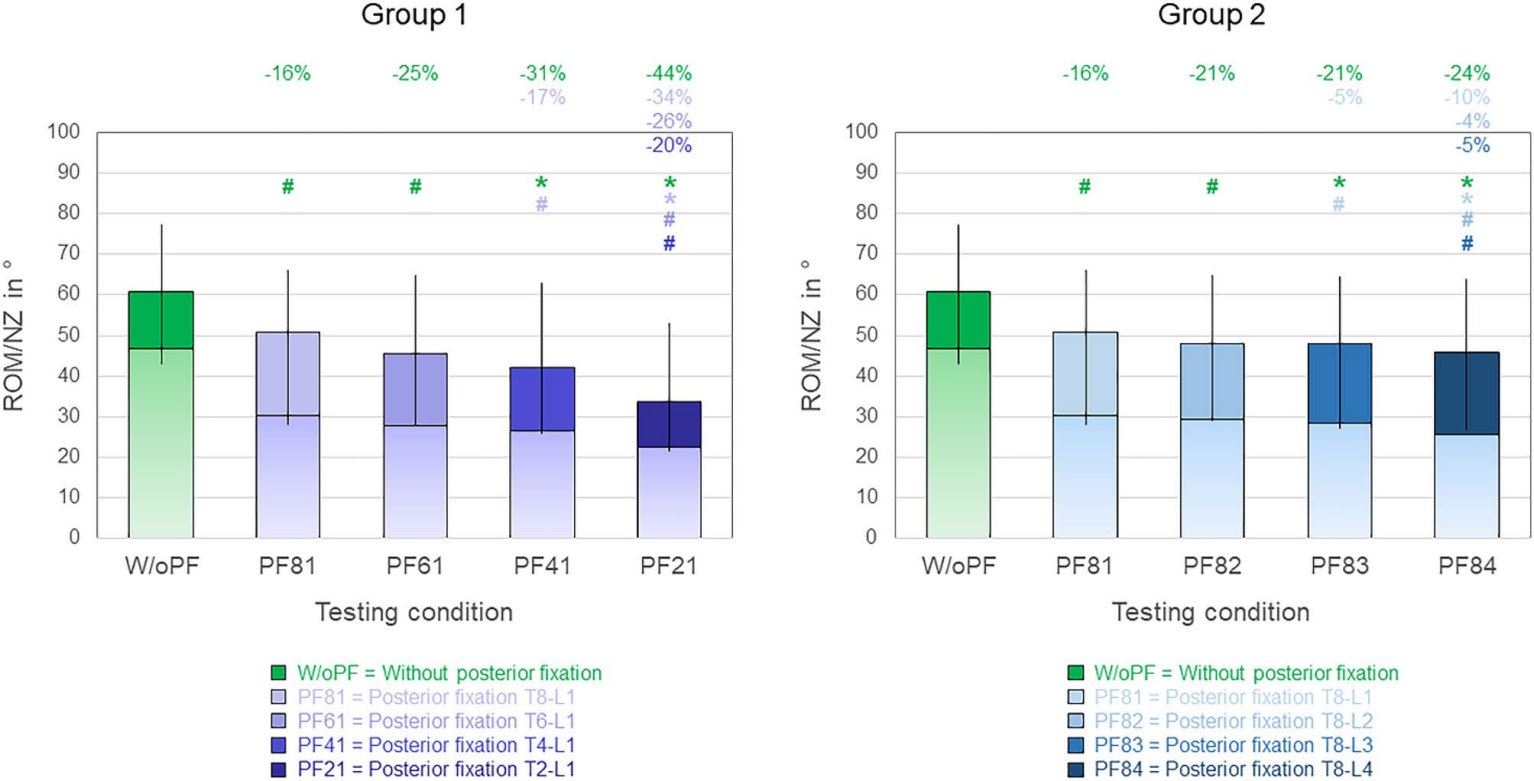
Overall (C7-S) range of motion (ROM, illustrated as single-color bars) and neutral zone (NZ, illustrated as bars with color gradient) in flexion/extension for both study groups (2 × n = 6), presented as medians with minimum and maximum values. Significant differences (p < 0.05) are marked with * (Friedman’s ANOVA with Bonferroni-Dunn post-hoc correction and pairwise comparisons) and # (sole pairwise Friedman test), each colored according to the respective condition. Data for the conditions without posterior fixation and with posterior fixation from T8 to L1 are identical for both groups.

**Fig. 4:**
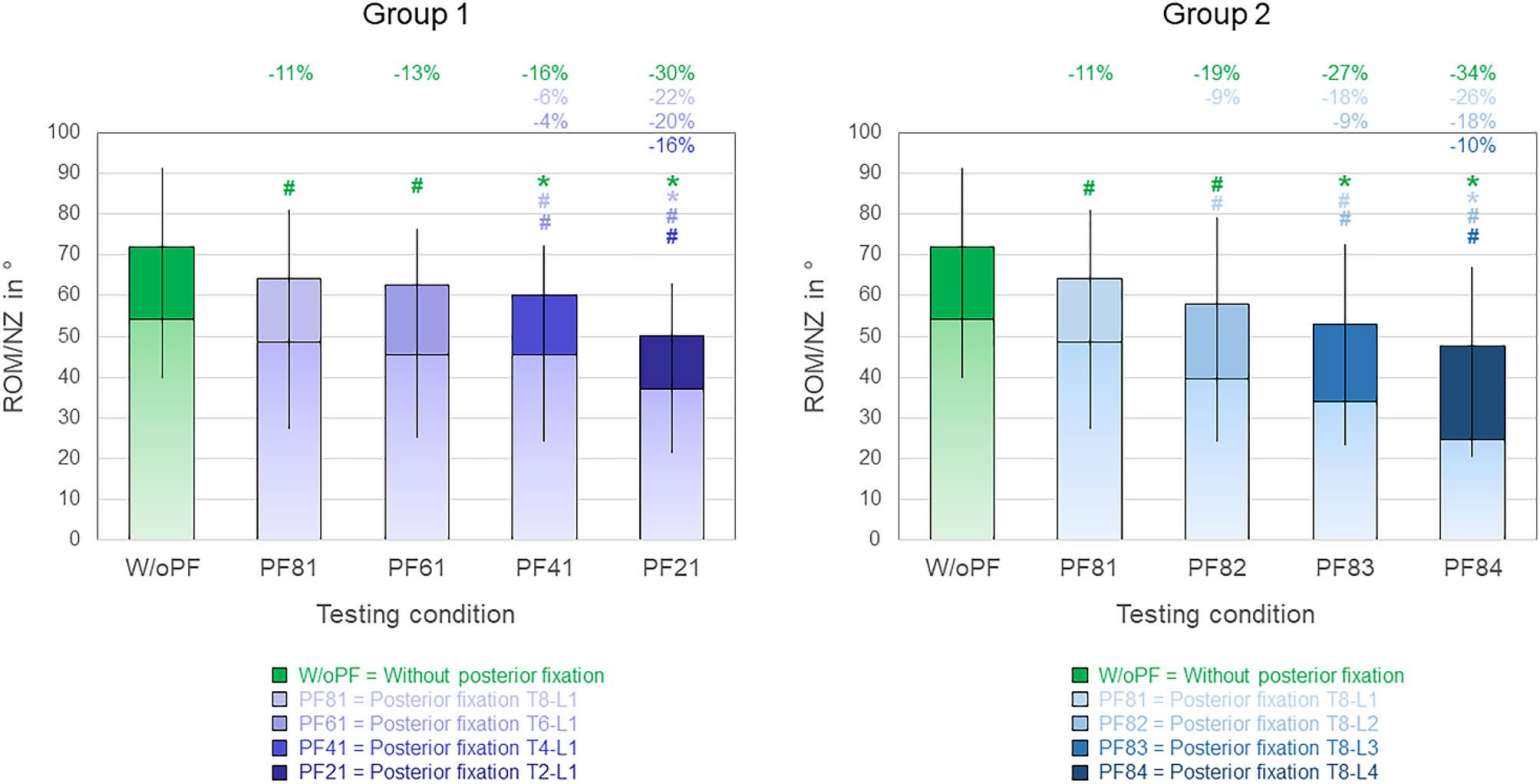
Overall (C7-S) range of motion (ROM, illustrated as single-color bars) and neutral zone (NZ, illustrated as bars with color gradient) in lateral bending for both study groups (2 × n = 6), presented as medians with minimum and maximum values. Significant differences (p < 0.05) are marked with * (Friedman’s ANOVA with Bonferroni-Dunn post-hoc correction and pairwise comparisons) and # (sole pairwise Friedman test), each colored according to the respective condition. Data for the conditions without posterior fixation and with posterior fixation from T8 to L1 are identical for both groups.

**Fig. 5:**
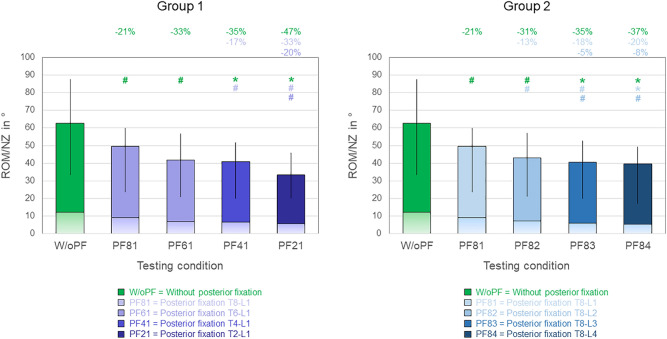
Overall (C7-S) range of motion (ROM, illustrated as single-color bars) and neutral zone (NZ, illustrated as bars with color gradient) in axial rotation for both study groups (2 × n = 6), presented as medians with minimum and maximum values. Significant differences (p < 0.05) are marked with * (Friedman’s ANOVA with Bonferroni-Dunn post-hoc correction and pairwise comparisons) and # (sole pairwise Friedman test), each colored according to the respective condition. Data for the conditions without posterior fixation and with posterior fixation from T8 to L1 are identical for both groups.

All tested posterior instrumentation lengths caused significant (*p* < 0.05) reductions of the overall range of motion (C7-S) compared to the condition without posterior fixation, respectively, in any motion plane (Figs. 3, 4, 5). The highest relative range of motion decreases were found in group 1 in axial rotation (− 21%, − 33%, − 35%, and − 47% for T8-L1, T6-L1, T4-L1, and T2-L1 instrumentations, respectively, Error! Reference source not found.). Moreover, every instrumentation length increase led to range of motion reduction compared to its respective shorter length, which, however, was not statistically significant in any case:

In flexion/extension (Fig. 3), no significant differences (*p* > 0.05) were detected between T8-L1 and T6-L1 instrumentations and between T6-L1 and T4-L1 instrumentations in group 1 as well as between T8-L1 and T8-L2 instrumentations and between T8-L2 and T8-L3 instrumentations in group 2. In group 1, highest relative range of motion decreases between single instrumentation lengths were found for T2-L1 instrumentation (− 34%, − 26%, and − 20% compared to T8-L1, T6-L1, and T4-L1 instrumentations, respectively), with all being significant (*p* < 0.05, Fig. 3 left). In group 2, differences were less pronounced, while highest relative range of motion decreases were caused by T8-L4 instrumentation (− 10%, − 4%, and − 5% compared to T8-L1, T8-L2, and T8-L4 instrumentation, respectively), also with all being significant (*p* < 0.05**, **Fig. 3 right).

In lateral bending (Fig. 4), every instrumentation length increase caused significant (*p* < 0.05) range of motion decrease, with the exception of T6-L1 instrumentation when compared with T8-L1 instrumentation in group 1. Highest relative range of motion decreases between single instrumentation lengths in group 1 were caused by T2-L1 instrumentation (− 22%, − 20%, and − 16% compared to T8-L1, T6-L1, and T4-L1 instrumentations, respectively), with all being significant (*p* < 0.05, Fig. 4 left). In group 2, a more gradual range of motion decrease was detected between the single instrumentation lengths with about 8–10% range of motion reduction per instrumentation length increase, while all range of motion decreases were significant (*p* < 0.05, Fig. 4 right).

In axial rotation (Fig. 5), significant (*p* < 0.05) range of motion decreases between directly consecutive instrumentation lengths were solely found in group 2 for T8-L2 and T8-L3 instrumentations compared to T8-L1 and T8-L2 instrumentations, respectively. In group 1, highest relative range of motion decreases between single instrumentation lengths were found for T2-L1 instrumentation (− 33% and − 20% compared to T8-L1 and T6-L1 instrumentations, respectively), with both being significant (*p* < 0.05, Fig. 5 left). In group 2, the highest relative range of motion decrease was detected for T8-L2 instrumentation (− 13% compared to T8-L1 instrumentation), which was also significant (*p* < 0.05, Fig. 5 right).

**Fig. 6:**
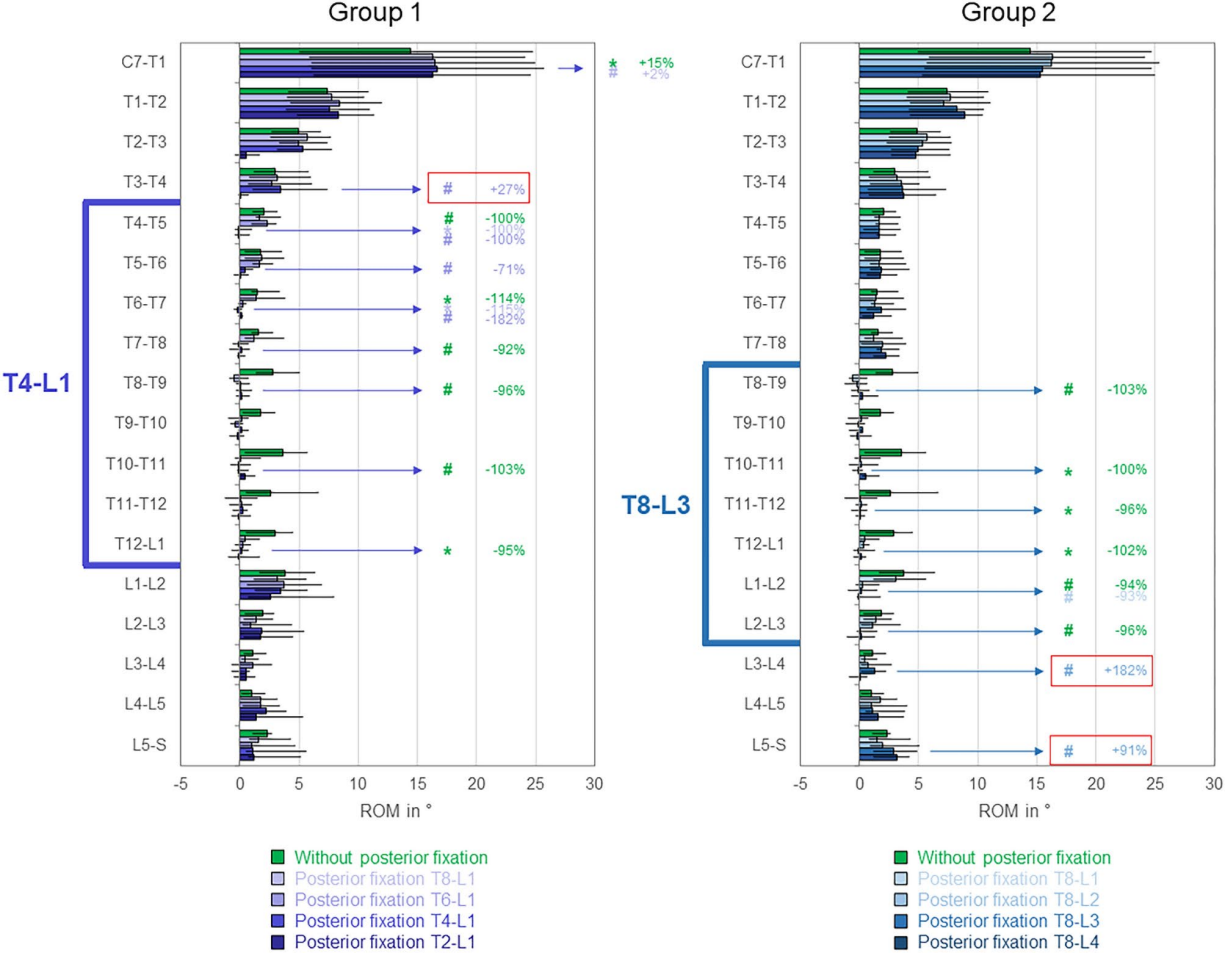
Segmental ranges of motion (ROM) in flexion/extension for the testing conditions of both study groups (2 × n = 6) exhibiting the highest ROM increases, presented as medians with minimum and maximum values. Significant differences (*p* < 0.05) are marked with * (Friedman’s ANOVA with Bonferroni-Dunn post-hoc correction and pairwise comparisons) and # (sole pairwise Friedman test), each colored according to the respective condition. Significant ROM increases of adjacent segments are bordered red. Data for the conditions without posterior fixation and with posterior fixation from T8 to L1 are identical for both groups.

**Fig. 7:**
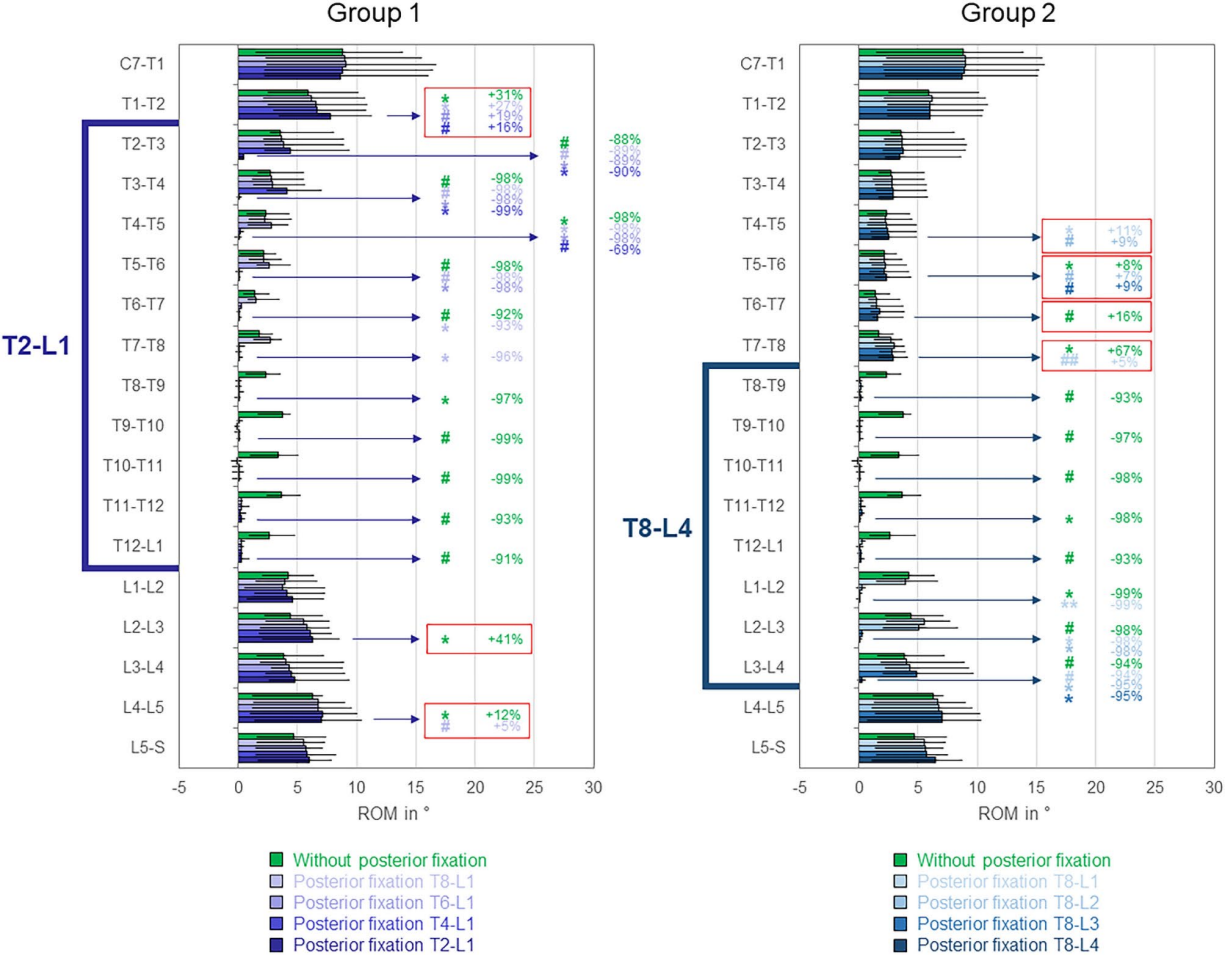
Segmental ranges of motion (ROM) in lateral bending for the testing conditions of both study groups (2 × n = 6) exhibiting the highest ROM increases, presented as medians with minimum and maximum values. Significant differences (*p* < 0.05) are marked with * (Friedman’s ANOVA with Bonferroni-Dunn post-hoc correction and pairwise comparisons) and # (sole pairwise Friedman test), each colored according to the respective condition. Significant ROM increases of adjacent segments are bordered red. Data for the conditions without posterior fixation and with posterior fixation from T8 to L1 are identical for both groups.

**Fig. 8:**
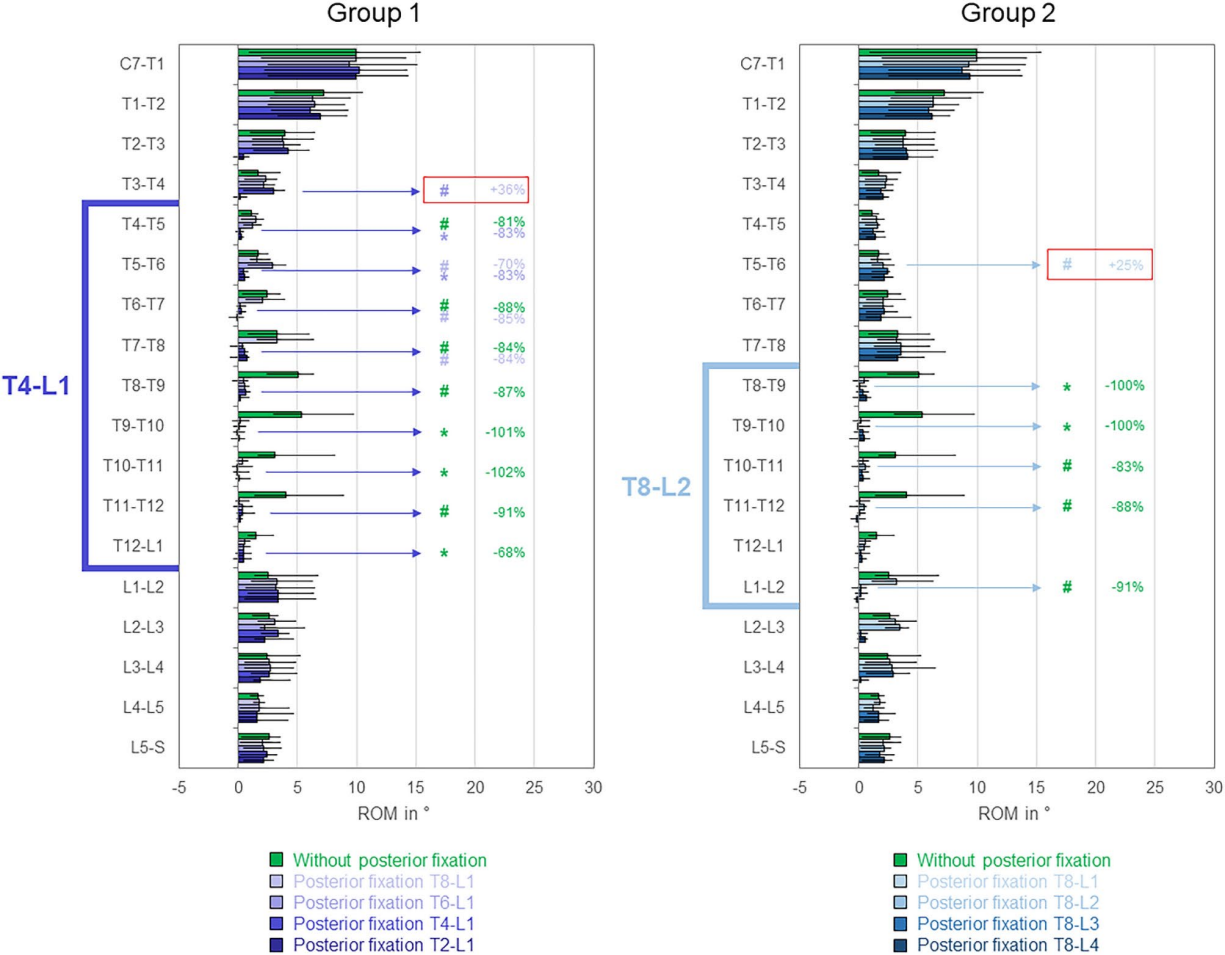
Segmental ranges of motion (ROM) in axial rotation for the testing conditions of both study groups (2 × n = 6) exhibiting the highest ROM increases, presented as medians with minimum and maximum values. Significant differences (*p* < 0.05) are marked with * (Friedman’s ANOVA with Bonferroni-Dunn post-hoc correction and pairwise comparisons) and # (sole pairwise Friedman test), each colored according to the respective condition. Significant ROM increases of adjacent segments are bordered red. Data for the conditions without posterior fixation and with posterior fixation from T8 to L1 are identical for both groups.

On the segmental level, all tested posterior instrumentation lengths led to significant (*p* < 0.05) reductions of the range of motion at the instrumented levels compared to the condition without posterior fixation in any motion plane (Figs. 6, 7, 8), with relative range of motion reductions of up to about 100%. In flexion/extension, significant (*p* < 0.05) range of motion increase at the proximal adjacent segment T3-T4 was found in group 1 for T4-L1 instrumentation compared to T6-L1 instrumentation (+ 27%, Fig. 6 left), whereas in group 2, significant (*p* < 0.05) range of motion increases were detected at the distal levels L3-L4 and L5-S for T8-L3 instrumentation compared to T8-L1 fixation (+ 182% and + 91%), respectively (Fig. 6 right).

In lateral bending, adjacent segment range of motion increases were found for all tested instrumentation lengths (Fig. 7 and supplementary material file ‘Results’). Significant (*p* < 0.05) proximal adjacent segment range of motion increases compared to the condition without posterior fixation were detected at T7-T8 level (+ 60%) for T8-L1 instrumentation (both groups), at T3-T4 level (+ 50%) for T4-L1 instrumentation and at T1-T2 level (+ 31%) for T2-L1 instrumentation (group 1), and at T7-T8 level for T8-L2 (+ 72%), T8-L3 (+ 61%), and T8-L4 (+ 67%, Fig. 7 right) instrumentations (group 2), respectively. At T1-T2 level, T2-L1 instrumentation even caused significant (*p* < 0.05) range of motion increases compared to all shorter instrumentation lengths in group 1 (+ 27%, + 19%, + 16%, Fig. 7 left), while in group 2, T8-L4 instrumentation led to significant (*p* < 0.05) range of motion increases in each of the four levels above instrumentation (Fig. 7 right). Moreover, significant (*p* < 0.05) distal level range of motion increases compared to the condition without posterior instrumentation were found at L2–L3 level (+ 25%) for T8-L1 instrumentation (both groups) as well as at L2–L3 (+ 41%) and L4–L5 (+ 12%) levels for T2-L1 instrumentation (group 1, Fig. 7 left).

In axial rotation, significant (*p* < 0.05) range of motion increase at the proximal adjacent segment was solely found at T3–T4 level for T4-L1 instrumentation compared to T6-L1 instrumentation in group 1 (+ 36%, Fig. 8 left). In group 2, no significant (*p* > 0.05) range of motion changes at the adjacent segments were detected, while the range of motion was significantly (*p* < 0.05) increased at the proximal level T5–T6 for T8-L2 instrumentation compared to T8-L1 instrumentation (+ 25%, Fig. 8 right).

**Fig. 9:**
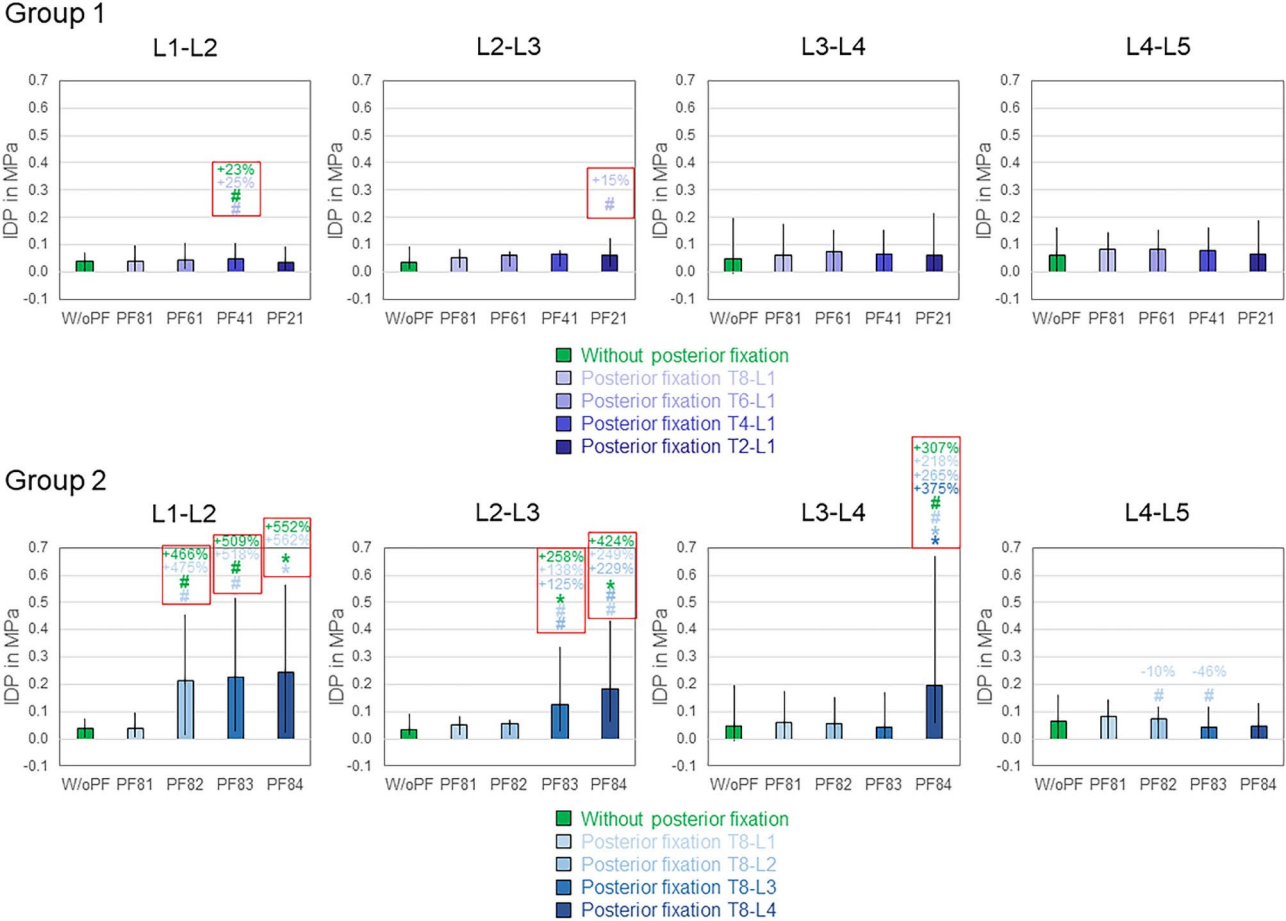
Intradiscal pressure (IDP) in flexion for both study groups (2 × n = 6), presented as medians with minimum and maximum values. Significant differences (*p* < 0.05) are marked with * (Friedman’s ANOVA with Bonferroni-Dunn post-hoc correction and pairwise comparisons) and # (sole pairwise Friedman test), each colored according to the respective condition. Significant IDP increases of instrumented and adjacent segments are bordered red. Data for the conditions without posterior fixation and with posterior fixation from T8 to L1 are identical for both groups.

Lumbar intradiscal pressure was predominantly affected in flexion direction (Fig. 9 and supplementary material file ‘Results’). While T8-L1 and T6-L1 instrumentations did not significantly (*p* > 0.05) alter the lumbar intradiscal pressure values in group 1, T4-L1 instrumentation caused significant (*p* < 0.05) intradiscal pressure increase compared to both the condition without posterior fixation (+ 23%) and T8-L1 instrumentation (+ 25%) at the distal adjacent segment L1-L2 (Fig. 9 top left). Moreover, T2-L1 instrumentation led to significantly (*p* < 0.05) increased intradiscal pressure compared to T8-L1 instrumentation at the distal level L2-L3. In group 2, instrumented levels exhibited enormous intradiscal pressure increases compared to the non-instrumented levels (Fig. 9 bottom row). At L1-L2 level, T8-L2, T8-L3, and T8-L4 instrumentations caused significant (*p* < 0.05) intradiscal pressure increases compared to both the condition without posterior instrumentation (+ 466%, + 509%, + 552%) and T8-L1 instrumentation (+ 475%, + 518%, + 562%), respectively, with the highest median intradiscal pressure of 0.244 MPa for T8-L4 instrumentation. At L2-L3 level, T8-L3 and T8-L4 instrumentations resulted in significant (p < 0.05) intradiscal pressure increases compared to the condition without posterior fixation (+ 258%, + 424%), T8-L1 instrumentation (+ 138%, + 249%), and T8-L2 instrumentation (+ 125%, + 229%). At L3-L4 level, T8-L4 instrumentation led to significantly (p < 0.05) increased intradiscal pressure compared to the condition without posterior instrumentation (+ 307%) as well as T8-L1 (+ 218%), T8-L2 (+ 265%), and T8-L3 (+ 375%) instrumentations and caused the highest maximum intradiscal pressure (0.670 MPa).

All compiled results of this study are retrievable from the supplementary material file ‘Results.pdf’.

## Discussion

Finding the optimal instrumentation length in terms of UIV and LIV represents an essential decision of spinal deformity surgeons with regard to deformity correction and its long-term preservation, while the instrumentation itself was found to cause frequent complications regarding adjacent segment disease and deformity in long-term follow-up studies^16–21^. This in vitro study found distinct effects of the instrumentation length on adjacent segment range of motion and lumbar intradiscal pressure for two specific types of scoliosis curves, potentially explaining these clinical complications.

All posterior instrumentation lengths tested in this in vitro study caused significant reductions of the overall range of motion compared to the condition without posterior fixation in any motion plane, confirming the results of previous in vivo studies^8,10,12,14^. Moreover, almost every instrumentation length increase led to further significant reduction of the overall range of motion in any motion plane, meaning that both the UIV and LIV affect the overall spinal flexibility. In particular, T2-L1 instrumentation was found to cause the highest relative range of motion decrease in any motion plane in this study, which might be explained by the additional stabilizing effect of the upper rib cage structures^31^, potentially further reinforcing proximal instrumentation length increase in the thoracic spine^29,32^. Furthermore, distinctly higher proximal adjacent segment range of motion increases were detected for both T2-L1 and T4-L1 instrumentations compared to T6-L1 and T8-L1 instrumentations within group 1. With regard to flexibility preservation, thoracic spinal instrumentation length increase in proximal direction should therefore be avoided in order to improve the post-operative patient comfort in terms of increased range of motion. Apart from that, the highest instrumentation lengths in both groups (T2-L1 and T8-L4, respectively) even caused significant range of motion increases at multiple uninstrumented levels in lateral bending, which were proximal (T1-T2) as well as distal (L2-L3, L4-L5) in case of T2-L1 instrumentation and which were proximally distributed among a total of four levels (T4-T5 to T7-T8) in case of T8-L4 instrumentation. In flexion/extension, in contrast, instrumentation length increases in proximal direction tended to promote an increase of range of motion at the proximal adjacent segment, whereas instrumentation length increases in distal direction tended to produce higher distal adjacent segment range of motion, overall indicating specific effects of instrumentation types (thoracic vs. thoracolumbar) on adjacent segment mobility in the frontal and sagittal plane. While one previous in vivo study reported higher lumbar adjacent segment range of motion with a more distal LIV (increasing from T10 to L4) in lateral bending but not in forward flexion^15^, the present in vitro study could partially confirm these results, since instrumentation length increase in distal direction primarily led to significant proximal adjacent segment range of motion increases in lateral bending, whereas there were no significant proximal and distal adjacent segment range of motion increases in flexion/extension compared to the uninstrumented condition. However, since various instrumentation lengths and configurations were evaluated in the in vivo study of Marks et al., comparability with the results of the present in vitro study is limited apart from differences regarding loading conditions and measuring techniques. Moreover, some of the findings of the present study regarding changes of adjacent segment ranges of motion, though being statistically significant, may be of low clinical significance due to their low relative change. Nevertheless, the results of these studies potentially explain common clinical complications following spinal instrumentation such as adjacent segment disease and associated proximal and distal junctional kyphosis as well as other pathologies such as coronal decompensation or adding-on, as both studies found increased motions in the adjacent segments, which might cause accelerated degenerative processes in these segments.

Clinical studies clearly revealed direct correlation of adjacent segment degeneration with the number of instrumented levels^17^ and negative correlation with the number of uninstrumented levels below the instrumentation^17,33^, potentially resulting in associated deformity^33^. Proximal junctional kyphosis, for instance, was found to be a compensatory mechanism following spinal instrumentation^34^. Interestingly, patients who received instrumentation of more than eleven vertebral levels and who had an UIV at T1-T3 levels exhibited higher risks of proximal junctional kyphosis compared to patients receiving shorter and lower instrumentation in clinical studies^18,35^. This corresponds well with the results of the present in vitro study, in which T2-L1 instrumentation resulted in significant overall range of motion reduction as well as adjacent segment range of motion increase. One explanation for this phenomenon might be the abrupt transition from rigid fixation to the flexible spine, as in vitro and in silico studies found reduced proximal adjacent segment stress using supralaminar hooks^36^ or using rods with a diameter transition and preservation of the posterior ligament complex^37^. Similarly, an in vitro study detected reduction of distal adjacent segment range of motion by replacing the LIV with posterior dynamic stabilization^38^, while results of clinical studies suggest that instrumentation should include the thoracolumbar junction to avoid distal junctional kyphosis^39–41^. Distinct distal adjacent segment range of motion increases in lateral bending found in the present study might also partially explain the clinical phenomenon of coronal decompensation, which represents an alteration of coronal balance following posterior instrumentation^42,43^. In order to prevent this common complication and to provide higher spinal mobility for the patient, a meta-analysis of comparative clinical studies therefore already suggested use of shorter instrumentation^44^, confirming the findings of the present in vitro study.

Impaired sagittal balance might also be caused by the altered load sharing situation below the instrumentation, as the present study detected specifically and slightly but statistically significantly increased intradiscal pressure at the segments distally adjacent to the longest instrumentations (T2-L1 and T4-L1), confirming the findings of a previous in vitro study^45^. However, it should also be noted that for most conditions, the intradiscal pressure at the distal adjacent levels was not significantly altered compared to the uninstrumented condition or the condition with the shortest instrumentation (T8-L1), suggesting overall minor effects of instrumentation on the loading of the distal adjacent segment. While intradiscal pressure measurements of the proximal adjacent segments were not feasible in this study due to small disc heights in the upper thoracic spine^46^, a previous in vitro study on the lower lumbar spine, applying combined flexion and shear loading in a materials testing machine, could at least prove that the proximal adjacent segment intradiscal pressure rises if the instrumentation length increases^47^. This indicates negative effects of instrumentation not only on distal but also on proximal adjacent segment intradiscal pressure. At the instrumented levels, the highest median intradiscal pressure was about 2.5 times higher and the highest maximum intradiscal pressure was even almost 7 times higher in the present study compared to the average lumbar intradiscal pressure in flexion assuming same loading conditions^48^, potentially explaining rapid fusion of adjacent instrumented vertebrae into solid bone in scoliosis patients. This phenomenon might be caused by high constraint forces on the intervertebral disc during flexion and an alteration of the kinematics in the sagittal plane at these levels due to the rigid instrumentation, as the posterior instrumentation exhibits the longest lever arms in forward bending.

Apart from limited comparability of the results of this in vitro study with in vivo findings regarding differences between pure moment loading and actual complex physiological loading in the human body^49,50^, actually requiring complex load protocols or muscle force simulation in order to completely evaluate adjacent segment flexibility, the present study used pure moments of 5 Nm for entire thoracolumbar spinal specimens. While pure moments of 5 Nm are recommended and widely accepted for the simulation of quasi-physiological loading of the thoracic spine during daily activities as well as for entire thoracolumbar spine specimens in order to prevent damage of the thoracic spine during repetitive loading^22^, the lumbar portion of the specimens might therefore not have fully reached the range of quasi-physiological loading, as the lumbar spine is standardly tested at 7.5 Nm^22^. Both the simplified loading protocol and the reduced moment on the lumbar spine could potentially have led to reduced effects of instrumentation length increase on adjacent segment range of motion and intradiscal pressure in the present study, whereas eccentric forces due to the dead weight of the specimens and missing stabilizing muscle forces could have led to higher effects. Nevertheless, this standardized approach provides the benefit of fully reproducible loading conditions and thus facilitates the interpretation of resulting data. Apart from the low sample size of n = 6, which could have limited the significance of the results while representing a common sample size for in vitro studies using human specimens due to ethical and financial reasons, testing of specimens without clinically relevant spinal deformity from young adult but non-adolescent donors represents a further limitation of this study. However, specimens from adolescent donors are generally not available due to ethical reasons, while specimens exhibiting scoliotic deformity are rarely accessible and usually quite heterogeneous regarding their deformity characteristics, which would reduce the reproducibility and interpretability of the results. Apart from that, the findings of this study can likewise be applied to adult scoliosis, as adjacent segment disease and junctional kyphosis also represent a highly relevant issue in adult spinal deformity. Nevertheless, the effects of pre-existing scoliotic deformity on adjacent segment range of motion and intradiscal pressure following posterior spinal instrumentation should be evaluated together with effects of further important parameters such as implant type, rod material, or rod diameter in future in vitro and in silico studies.

To conclude, this in vitro study found adverse effects of increasing instrumentation length in terms of the upper and lower instrumented vertebra including reduced overall spinal flexibility and increased proximal and distal adjacent segment range of motion when compared to the condition without any instrumentation as well as compared to other instrumentations in lateral bending for both thoracic and thoracolumbar curve type instrumentations, as well as specifically and slightly increased lumbar adjacent segment intradiscal pressure solely for one instrumentation configuration (T4-L1) and extremely increased lumbar intradiscal pressure at instrumented segments for increasing thoracolumbar instrumentation in distal direction. With regard to flexibility preservation and reduction of risks of long-term complications such as adjacent segment disease and associated proximal and distal junctional deformity, surgical treatment of patients should therefore prefer shorter instrumentations, if feasible considering the type and degree of deformity and adequate post-operative stability of the correction procedure in order to achieve a balance between clinical and biomechanical stability. Furthermore, this study provides reference data for future in vitro studies, validation data for future in silico studies, as well as basic biomechanical data for clinical studies and surgical decision making with regard to instrumentation for thoracic and thoracolumbar scoliosis curve types.

## Approval for human experiments

The use of the specimens was approved by the ethics committee board of the University of Ulm (63/17).

## Supplementary Information

Supplementary Information.

## Data Availability

All results including the statistical evaluations are retrievable from the supplementary material file ‘Results’ which is attached to the online version of this publication.
