# Supplementary material for: Spinal instrumentation length affects adjacent segment range of motion and intradiscal pressure
Source: Sci Rep. 2024 Dec 16;14:30496. doi: 10.1038/s41598-024-82132-0 (PMC11649938; doi:10.1038/s41598-024-82132-0)
Supplement: Supplementary file 1 — Supplementary Information. [file 41598_2024_82132_MOESM1_ESM.pdf]

Group 1: Increasing fixation length in upward direction

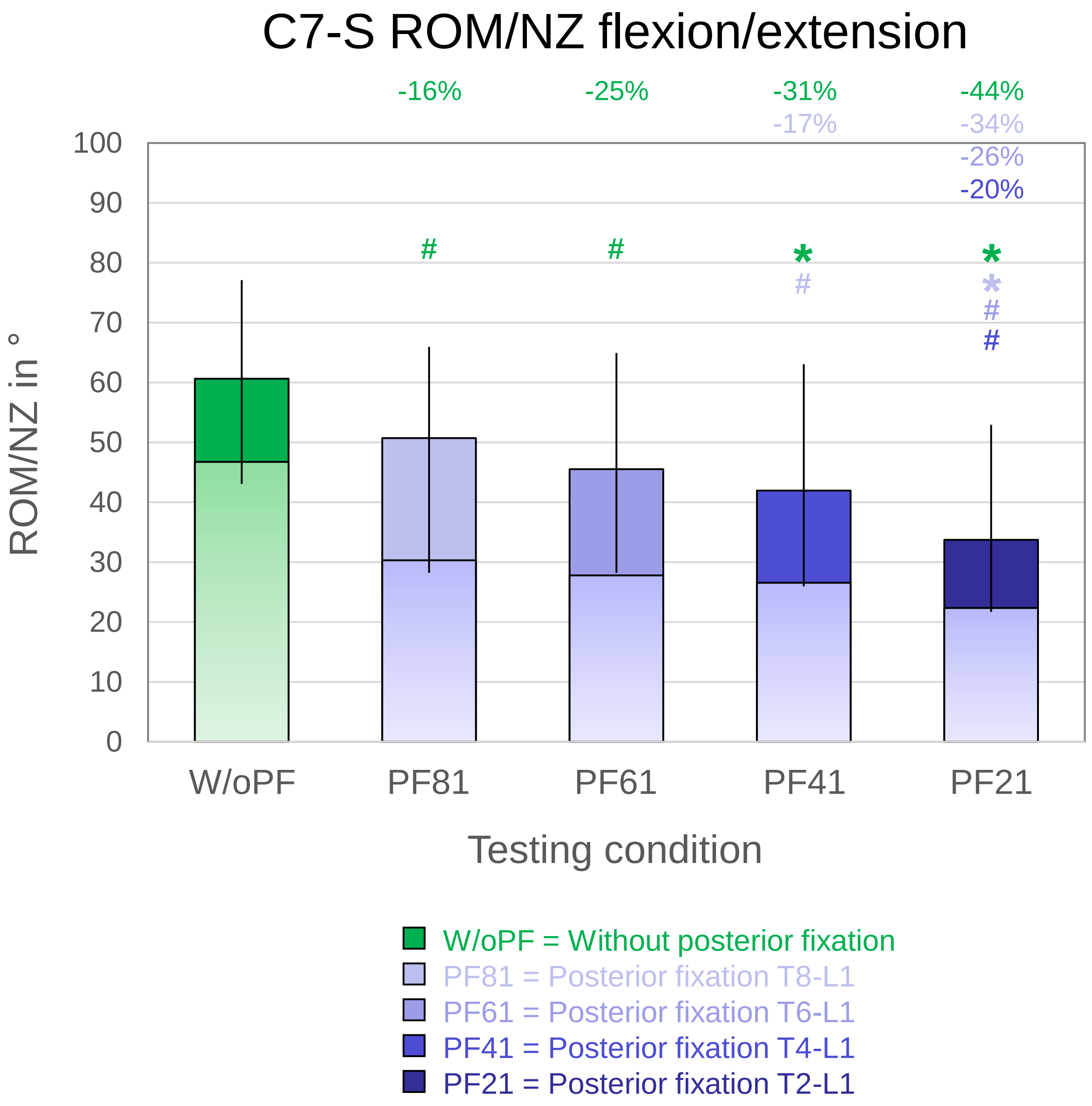

Significant change (p<0.05) compared to

* Without posterior fixation
* Posterior fixation T8-L1
* Posterior fixation T6-L1
* Posterior fixation T4-L1

tested with Friedman's ANOVA
+ Bonferroni-Dunn post-hoc correction
+ pairwise comparisons

Without posterior fixation
Posterior fixation T8-L1
Posterior fixation T6-L1
Posterior fixation T4-L1

tested with additional pairwise Friedman test without post-hoc correction

Group 2: Increasing fixation length in downward direction

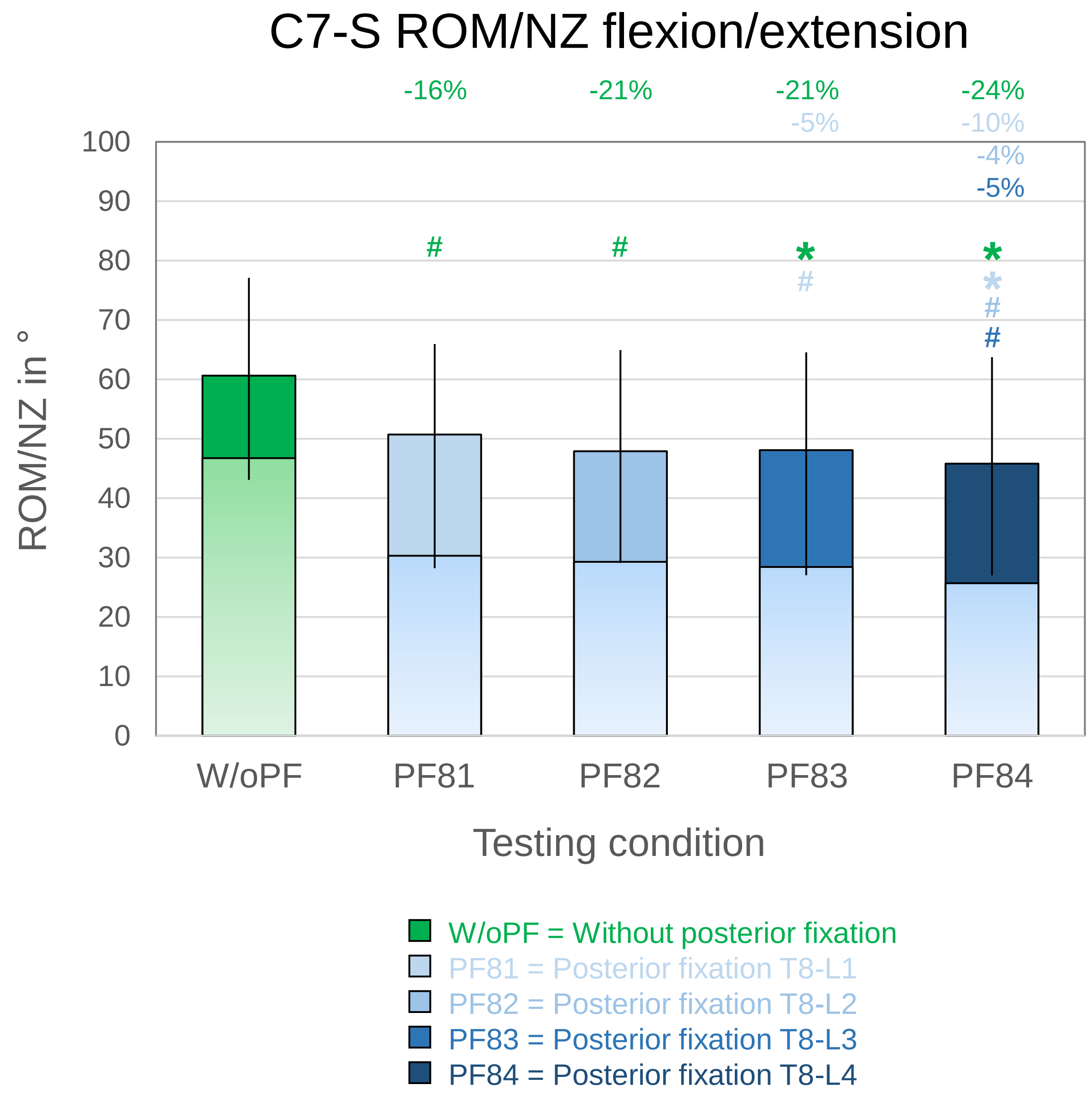

Significant change (p<0.05) compared to

* Without posterior fixation
* Posterior fixation T8-L1
* Posterior fixation T8-L2
* Posterior fixation T8-L3

tested with Friedman's ANOVA
+ Bonferroni-Dunn post-hoc correction
+ pairwise comparisons

Without posterior fixation
Posterior fixation T8-L1
Posterior fixation T8-L2
Posterior fixation T8-L3

tested with additional pairwise Friedman test without post-hoc correction

Group 1: Increasing fixation length in upward direction

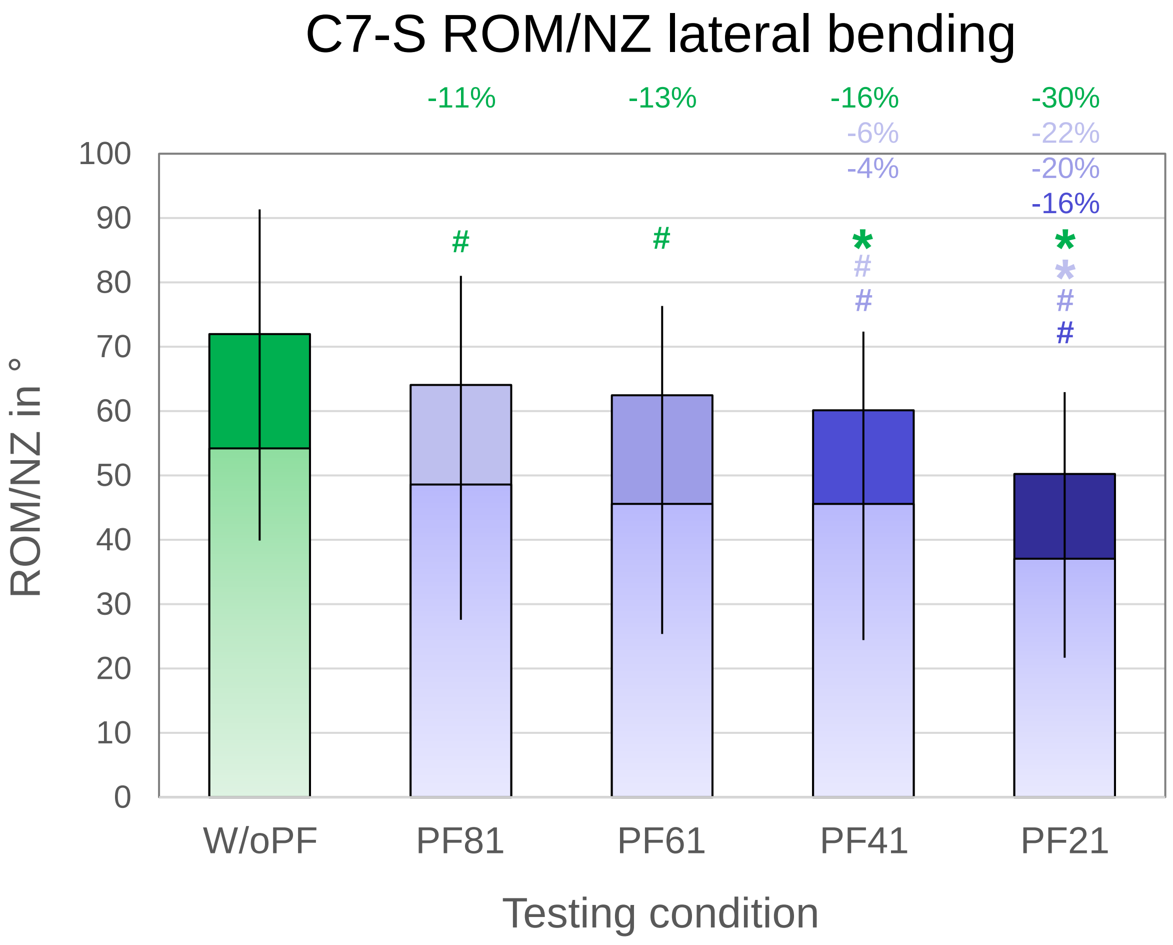

Significant change (p<0.05) compared to

- * Without posterior fixation
- * Posterior fixation T8-L1
- * Posterior fixation T6-L1
- * Posterior fixation T4-L1

tested with Friedman's ANOVA
+ Bonferroni-Dunn post-hoc correction
+ pairwise comparisons

- # Without posterior fixation
- # Posterior fixation T8-L1
- # Posterior fixation T6-L1
- # Posterior fixation T4-L1

tested with additional pairwise Friedman test without post-hoc correction

- W/oPF = Without posterior fixation
- PF81 = Posterior fixation T8-L1
- PF61 = Posterior fixation T6-L1
- PF41 = Posterior fixation T4-L1
- PF21 = Posterior fixation T2-L1

Group 2: Increasing fixation length in downward direction

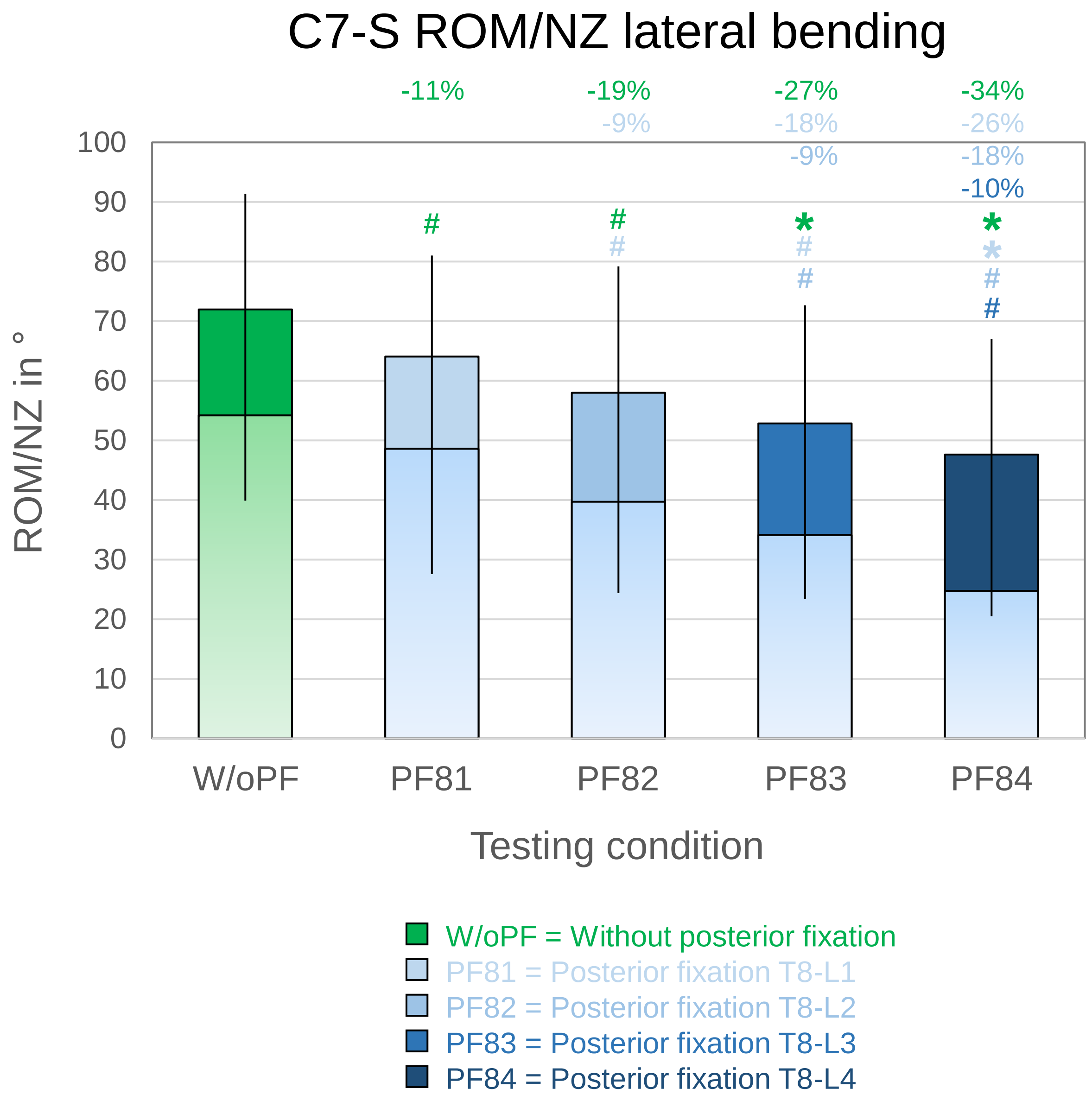

Significant change (p<0.05) compared to

* Without posterior fixation
* Posterior fixation T8-L1
* Posterior fixation T8-L2
* Posterior fixation T8-L3

tested with Friedman's ANOVA
+ Bonferroni-Dunn post-hoc correction
+ pairwise comparisons

Without posterior fixation
Posterior fixation T8-L1
Posterior fixation T8-L2
Posterior fixation T8-L3

tested with additional pairwise Friedman test without post-hoc correction

Group 1: Increasing fixation length in upward direction

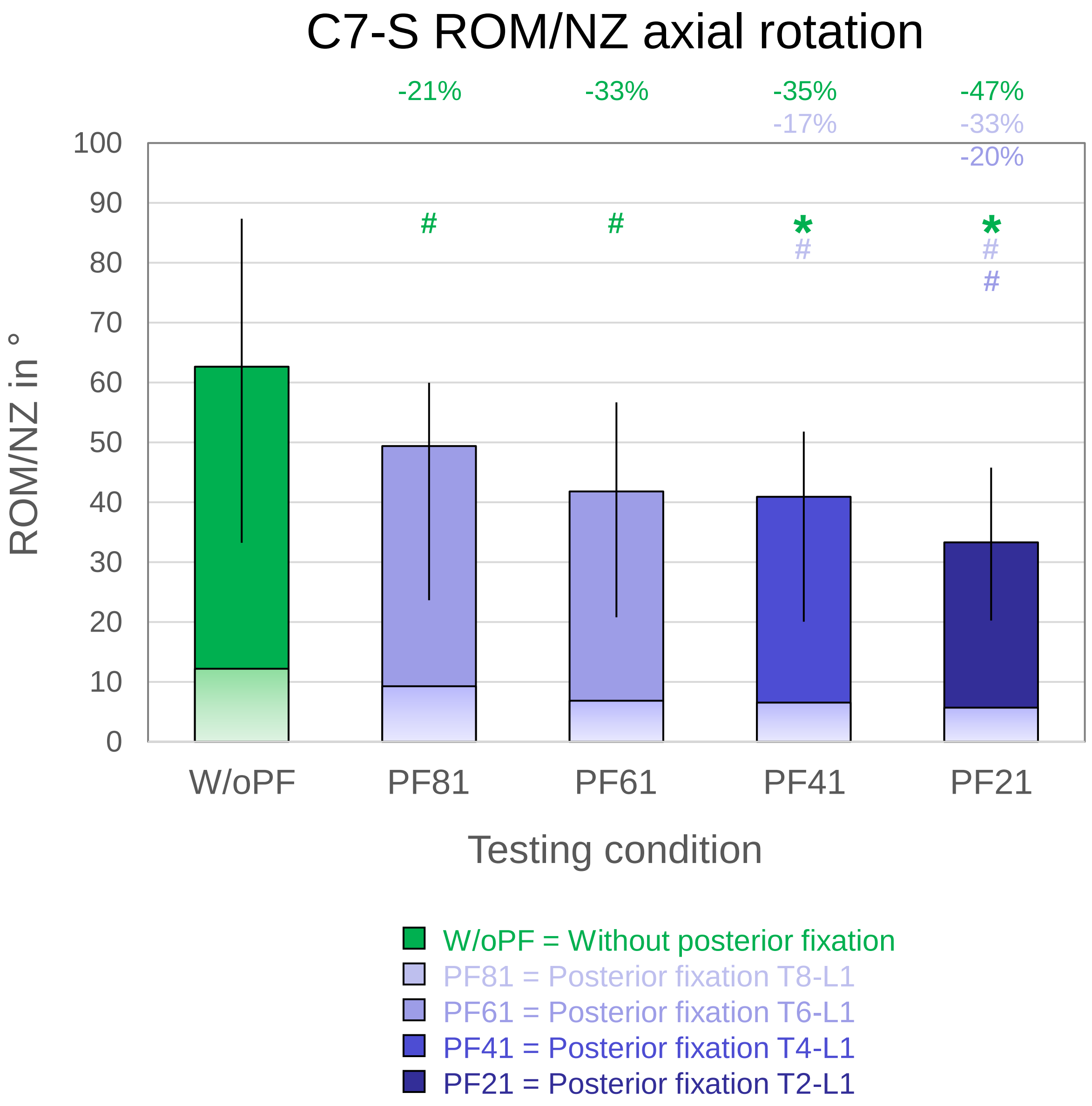

Significant change (p<0.05) compared to

* Without posterior fixation
* Posterior fixation T8-L1
* Posterior fixation T6-L1
* Posterior fixation T4-L1

tested with Friedman's ANOVA
+ Bonferroni-Dunn post-hoc correction
+ pairwise comparisons

Without posterior fixation
Posterior fixation T8-L1
Posterior fixation T6-L1
Posterior fixation T4-L1

tested with additional pairwise Friedman test without post-hoc correction

Group 2: Increasing fixation length in downward direction

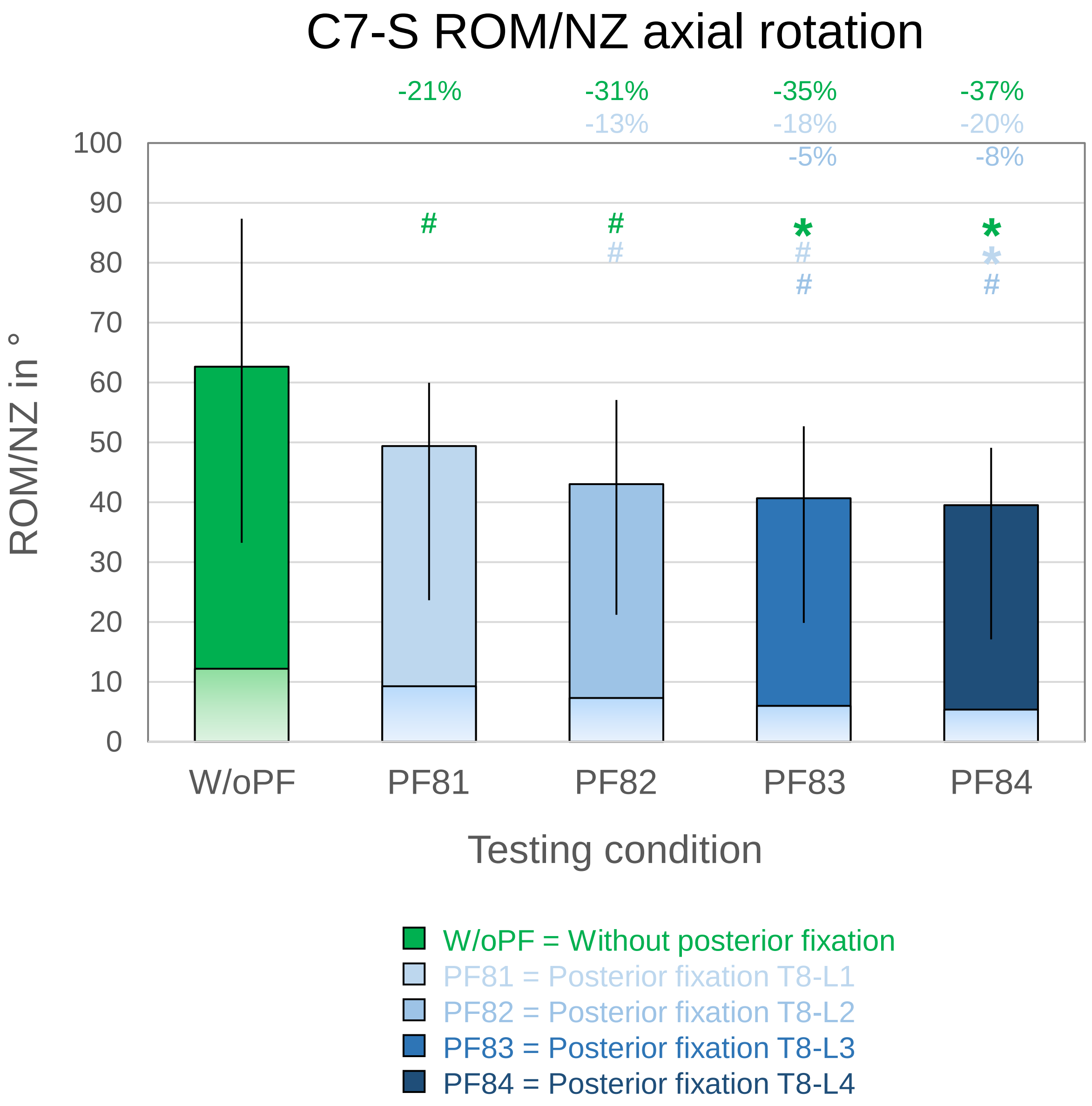

Significant change (p<0.05) compared to

* Without posterior fixation
* Posterior fixation T8-L1
* Posterior fixation T8-L2
* Posterior fixation T8-L3

tested with Friedman's ANOVA
+ Bonferroni-Dunn post-hoc correction
+ pairwise comparisons

Without posterior fixation
Posterior fixation T8-L1
Posterior fixation T8-L2
Posterior fixation T8-L3

tested with additional pairwise Friedman test without post-hoc correction

Segmental ROM flexion/extension

Group 1

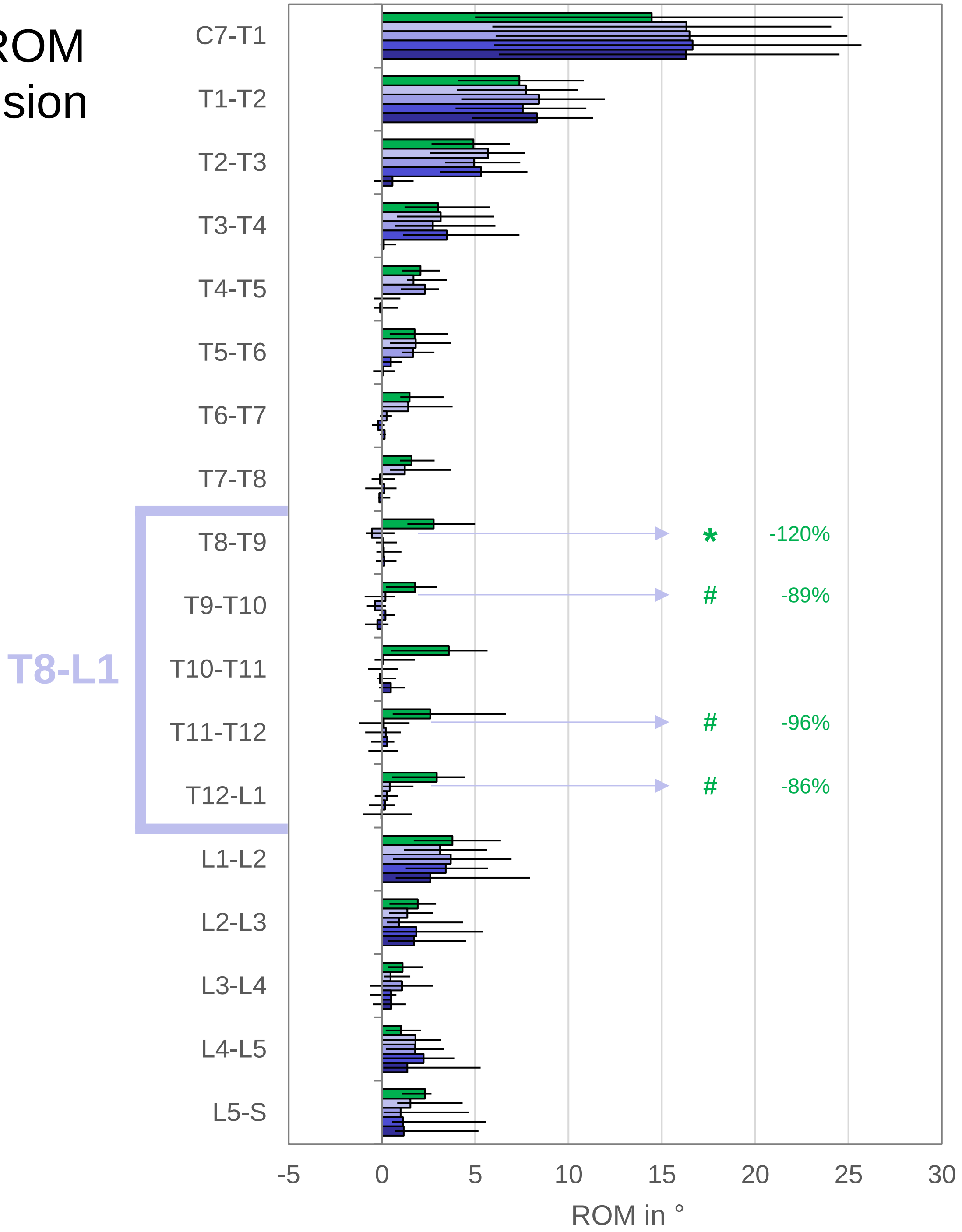

Effects of posterior fixation from T8 to L1

- Without posterior fixation
- Posterior fixation T8-L1
- Posterior fixation T6-L1
- Posterior fixation T4-L1
- Posterior fixation T2-L1

Significant change ($p<0.05$) compared to

* Without posterior fixation
* Posterior fixation T8-L1
* Posterior fixation T6-L1
* Posterior fixation T4-L1

tested with Friedman's ANOVA
+ Bonferroni-Dunn post-hoc correction
+ pairwise comparisons

Without posterior fixation
Posterior fixation T8-L1
Posterior fixation T6-L1
Posterior fixation T4-L1

tested with additional pairwise Friedman test without post-hoc correction

Segmental ROM flexion/extension

Group 1

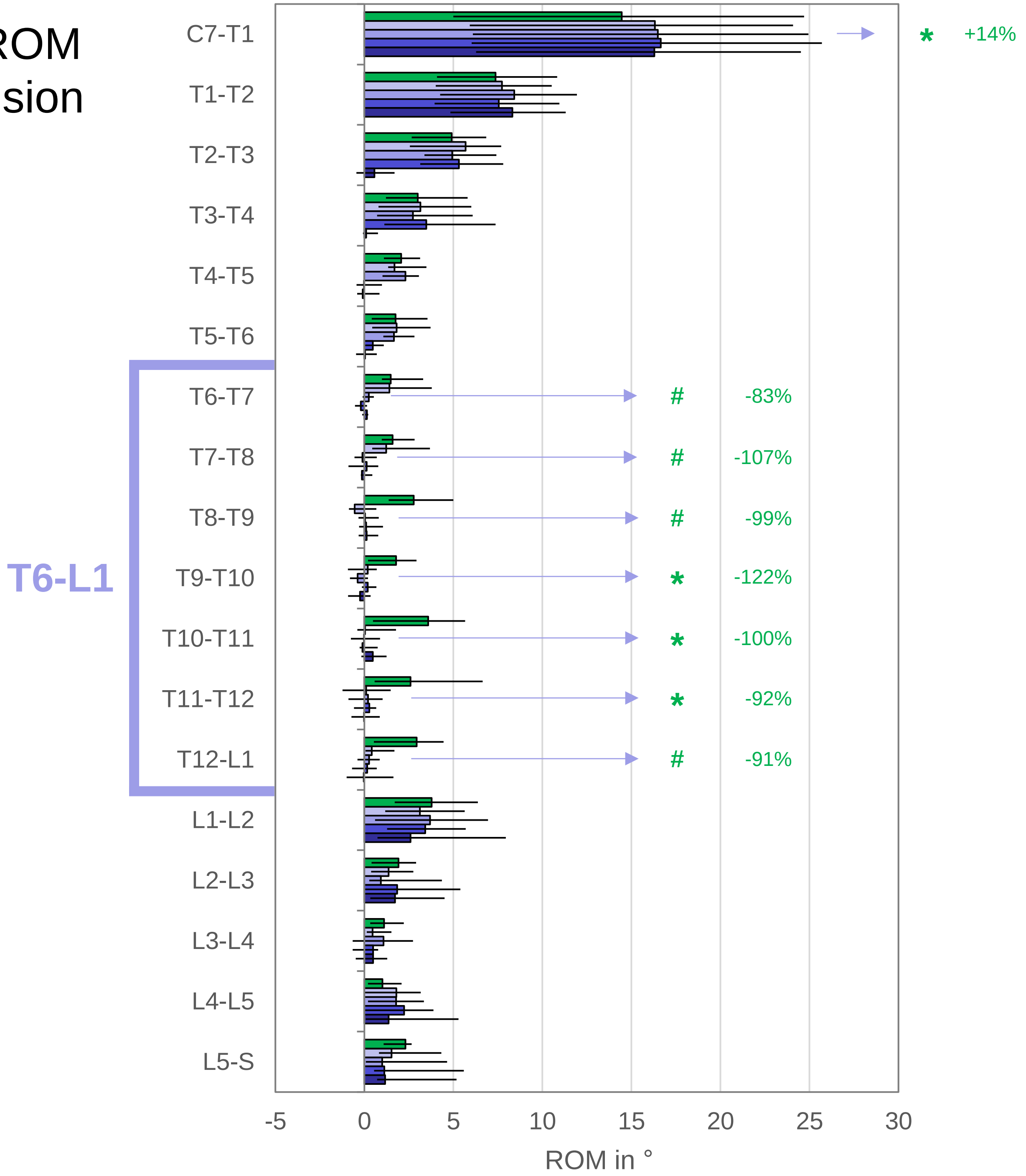

Effects of posterior fixation from T6 to L1

- Without posterior fixation
- Posterior fixation T8-L1
- Posterior fixation T6-L1
- Posterior fixation T4-L1
- Posterior fixation T2-L1

Significant change (p<0.05) compared to

* Without posterior fixation
* Posterior fixation T8-L1
* Posterior fixation T6-L1
* Posterior fixation T4-L1

tested with Friedman's ANOVA
+ Bonferroni-Dunn post-hoc correction
+ pairwise comparisons

Without posterior fixation
Posterior fixation T8-L1
Posterior fixation T6-L1
Posterior fixation T4-L1

tested with additional pairwise Friedman test without post-hoc correction

Segmental ROM flexion/extension

Group 1

Effects of posterior fixation from T4 to L1

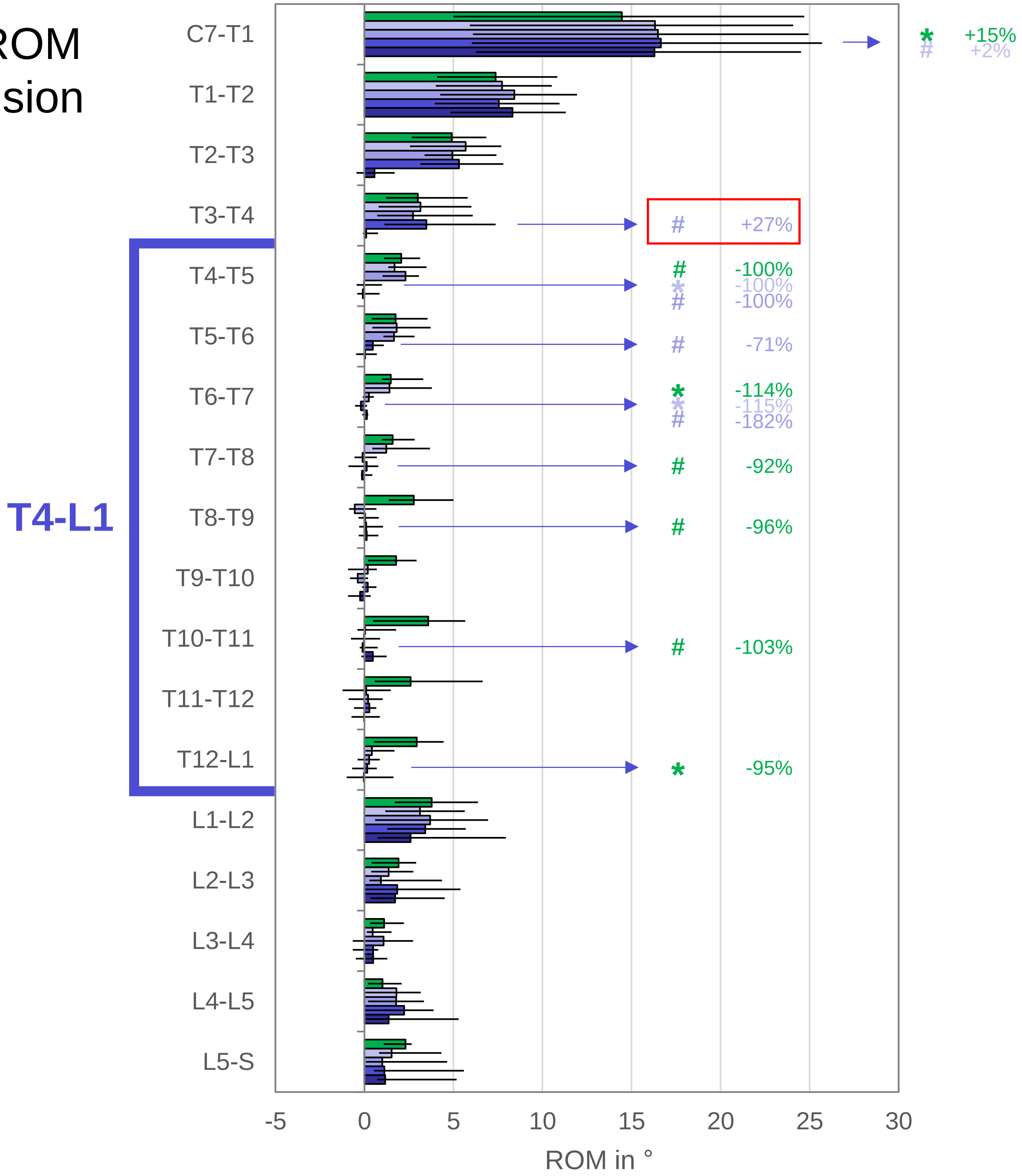

- Without posterior fixation
- Posterior fixation T8-L1
- Posterior fixation T6-L1
- Posterior fixation T4-L1
- Posterior fixation T2-L1

Significant change (p<0.05) compared to

* Without posterior fixation
* Posterior fixation T8-L1
* Posterior fixation T6-L1
* Posterior fixation T4-L1

tested with Friedman's ANOVA
+ Bonferroni-Dunn post-hoc correction
+ pairwise comparisons

Without posterior fixation
Posterior fixation T8-L1
Posterior fixation T6-L1
Posterior fixation T4-L1

tested with additional pairwise Friedman test without post-hoc correction

Segmental ROM flexion/extension

Group 1

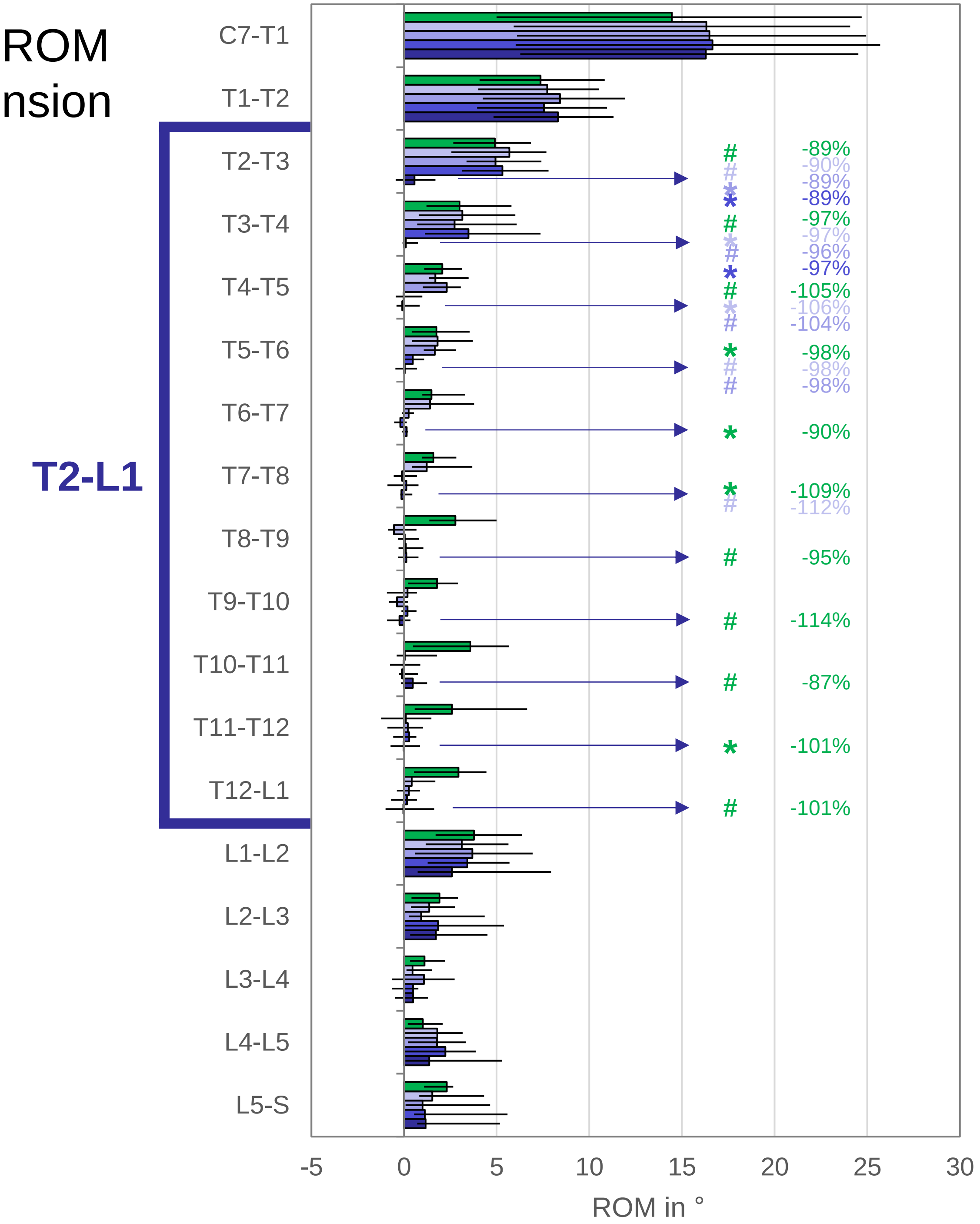

Effects of posterior fixation from T2 to L1

- Without posterior fixation
- Posterior fixation T8-L1
- Posterior fixation T6-L1
- Posterior fixation T4-L1
- Posterior fixation T2-L1

Significant change (p<0.05) compared to

* Without posterior fixation
* Posterior fixation T8-L1
* Posterior fixation T6-L1
* Posterior fixation T4-L1

tested with Friedman's ANOVA
+ Bonferroni-Dunn post-hoc correction
+ pairwise comparisons

Without posterior fixation
Posterior fixation T8-L1
Posterior fixation T6-L1
Posterior fixation T4-L1

tested with additional pairwise Friedman test without post-hoc correction

Segmental ROM
flexion/extension

Group 2

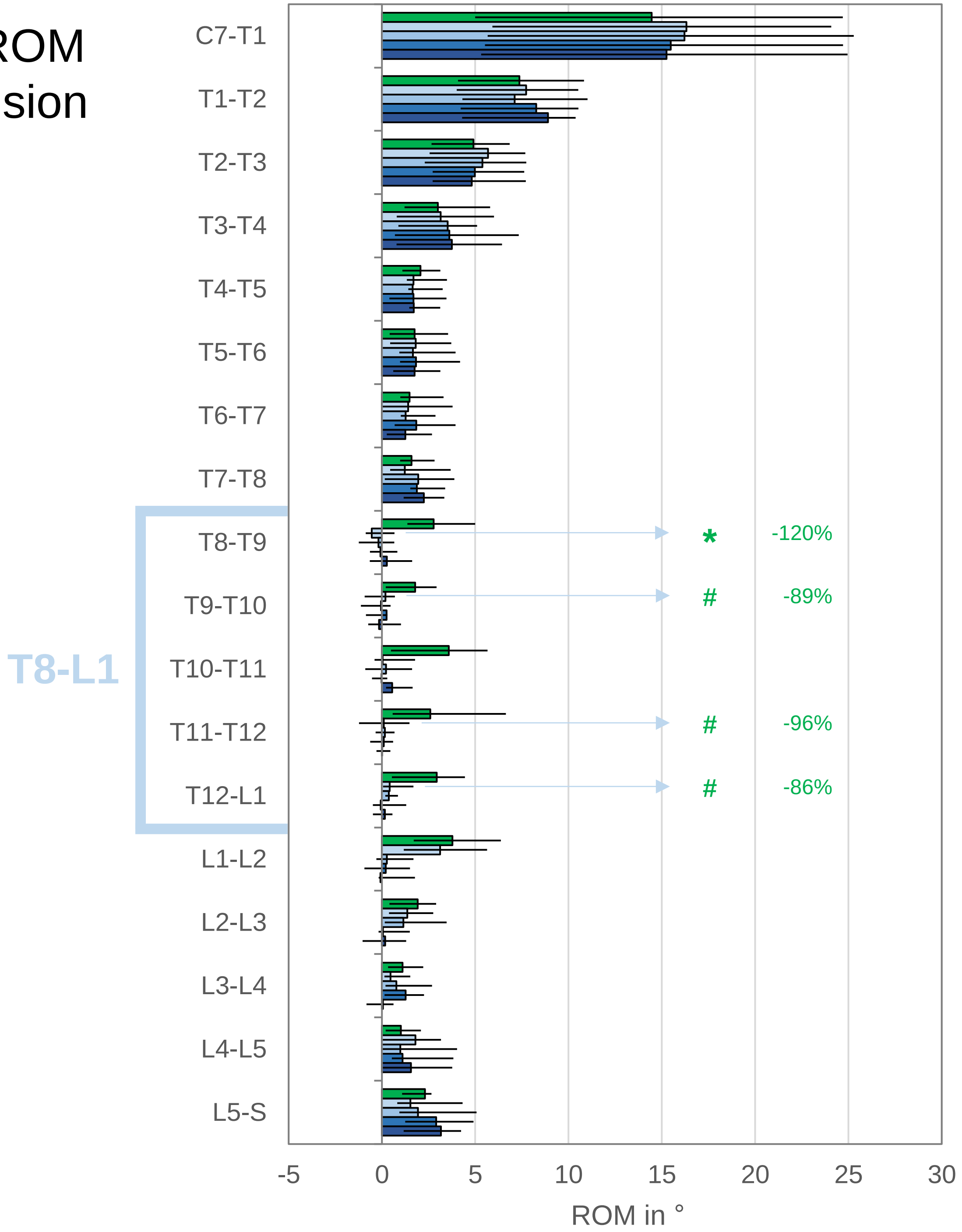

Effects of posterior fixation from T8 to L1

- Without posterior fixation
- Posterior fixation T8-L1
- Posterior fixation T8-L2
- Posterior fixation T8-L3
- Posterior fixation T8-L4

Significant change (p<0.05) compared to

* Without posterior fixation
* Posterior fixation T8-L1
* Posterior fixation T8-L2
* Posterior fixation T8-L3

tested with Friedman's ANOVA
+ Bonferroni-Dunn post-hoc correction
+ pairwise comparisons

Without posterior fixation
Posterior fixation T8-L1
Posterior fixation T8-L2
Posterior fixation T8-L3

tested with additional pairwise Friedman test
without post-hoc correction

Segmental ROM flexion/extension

Group 2

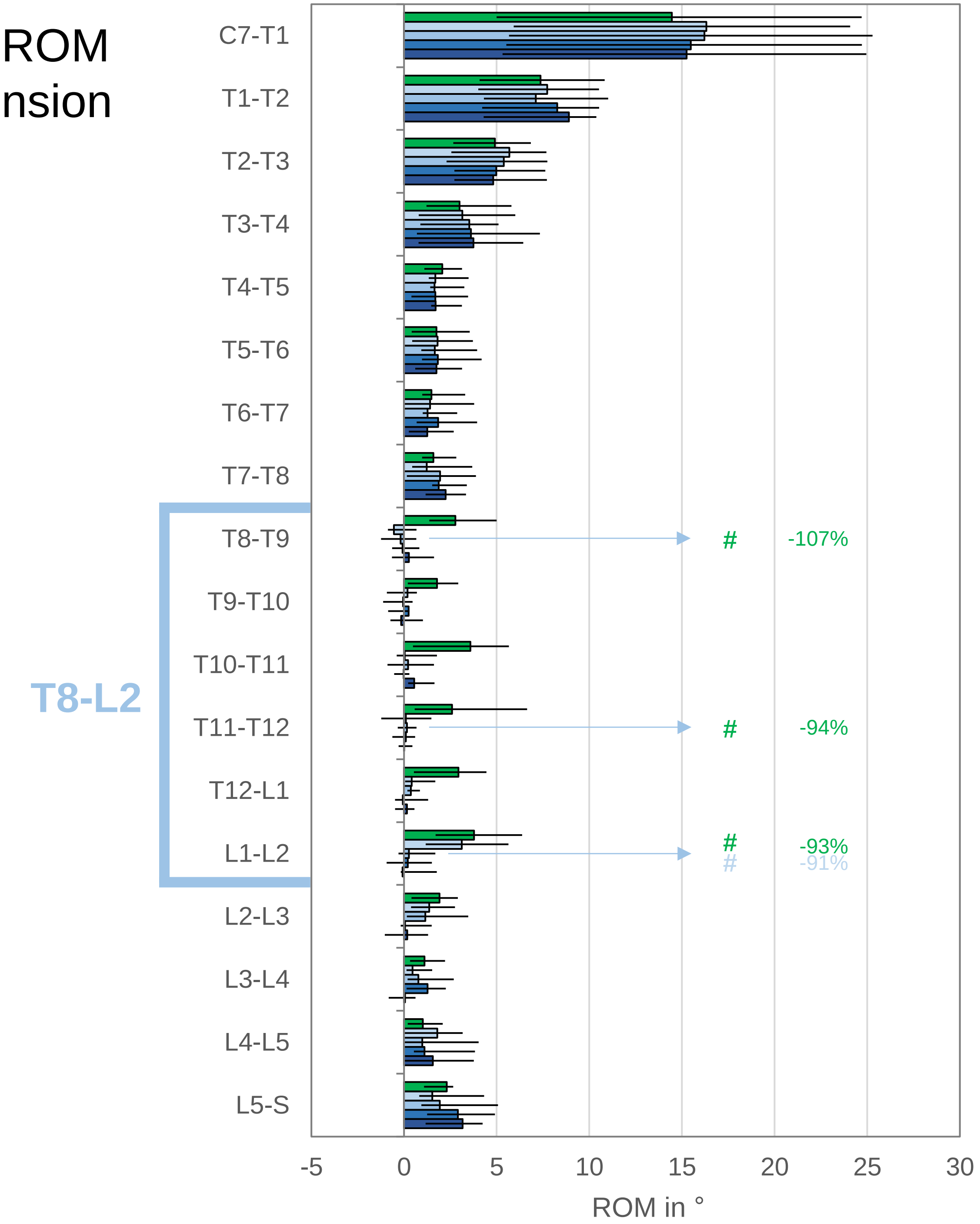

Effects of posterior fixation from T8 to L2

- Without posterior fixation
- Posterior fixation T8-L1
- Posterior fixation T8-L2
- Posterior fixation T8-L3
- Posterior fixation T8-L4

Significant change (p<0.05) compared to

* Without posterior fixation
* Posterior fixation T8-L1
* Posterior fixation T8-L2
* Posterior fixation T8-L3

tested with Friedman's ANOVA
+ Bonferroni-Dunn post-hoc correction
+ pairwise comparisons

Without posterior fixation
Posterior fixation T8-L1
Posterior fixation T8-L2
Posterior fixation T8-L3

tested with additional pairwise Friedman test without post-hoc correction

Segmental ROM flexion/extension

Group 2

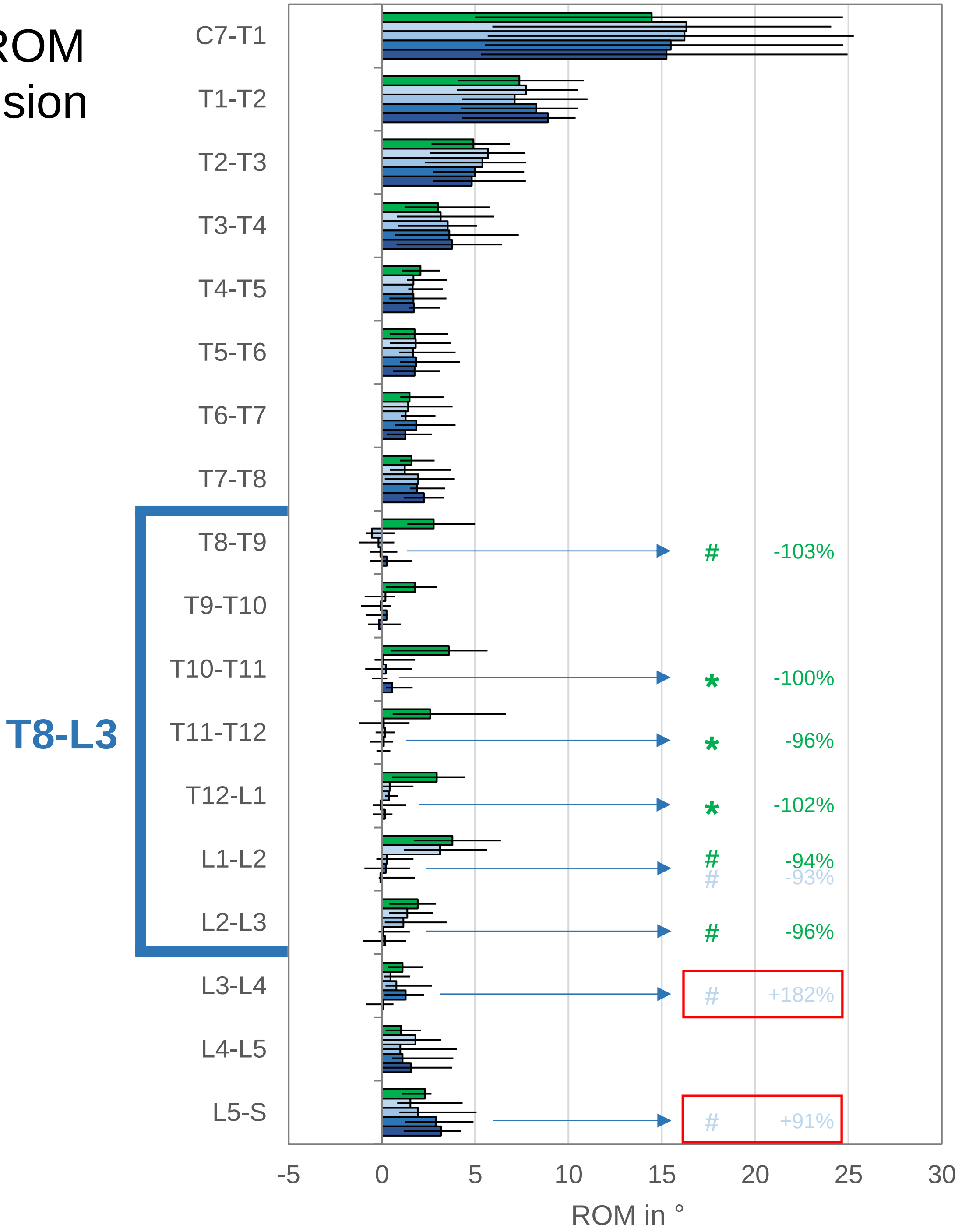

Effects of posterior fixation from T8 to L3

- Without posterior fixation
- Posterior fixation T8-L1
- Posterior fixation T8-L2
- Posterior fixation T8-L3
- Posterior fixation T8-L4

Significant change (p<0.05) compared to

* Without posterior fixation
* Posterior fixation T8-L1
* Posterior fixation T8-L2
* Posterior fixation T8-L3

tested with Friedman's ANOVA
+ Bonferroni-Dunn post-hoc correction
+ pairwise comparisons

Without posterior fixation
Posterior fixation T8-L1
Posterior fixation T8-L2
Posterior fixation T8-L3

tested with additional pairwise Friedman test without post-hoc correction

Segmental ROM flexion/extension

Group 2

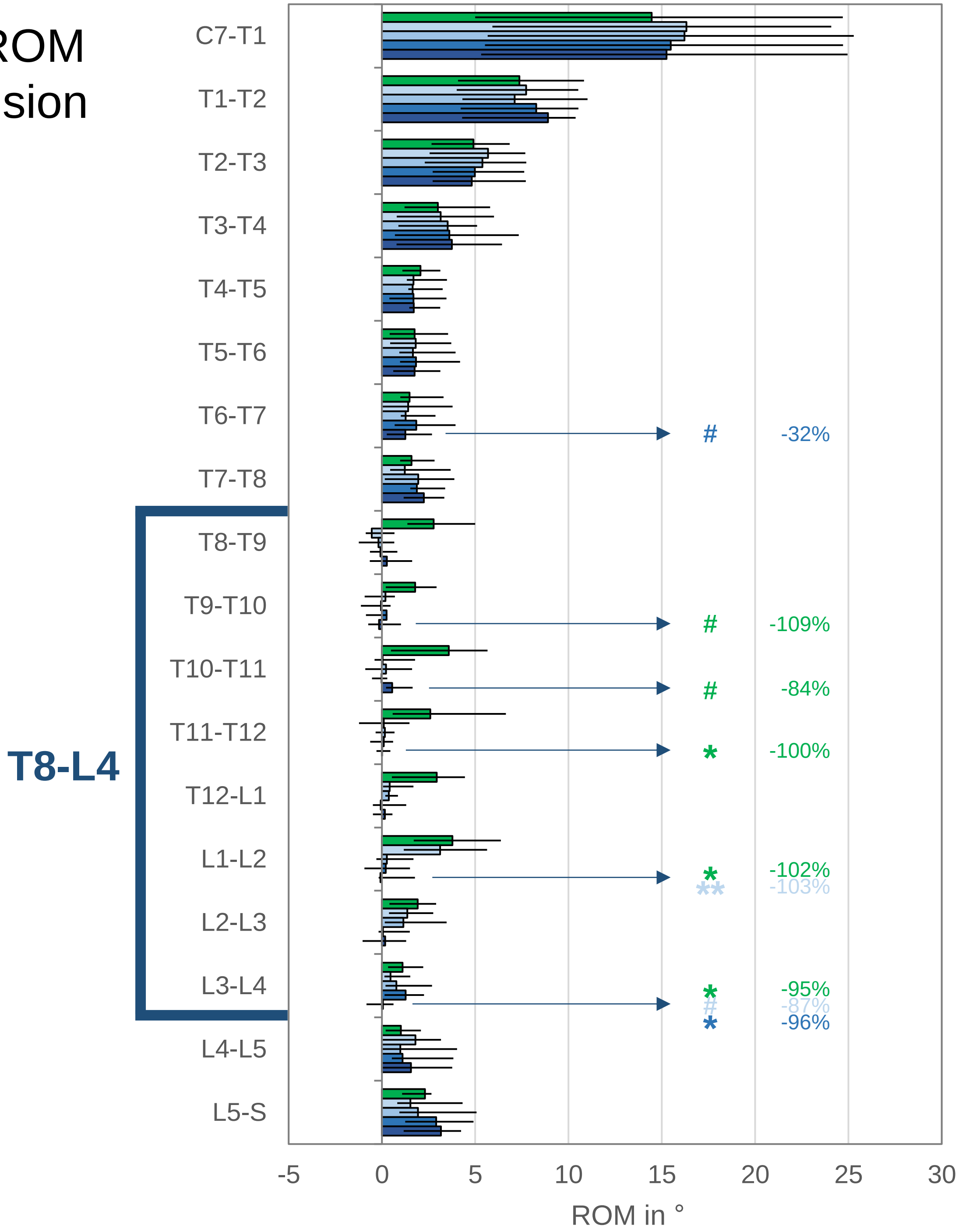

Effects of posterior fixation from T8 to L4

- Without posterior fixation
- Posterior fixation T8-L1
- Posterior fixation T8-L2
- Posterior fixation T8-L3
- Posterior fixation T8-L4

Significant change ($p<0.05$) compared to

* Without posterior fixation
* Posterior fixation T8-L1
* Posterior fixation T8-L2
* Posterior fixation T8-L3

tested with Friedman’s ANOVA
+ Bonferroni-Dunn post-hoc correction
+ pairwise comparisons

Without posterior fixation
Posterior fixation T8-L1
Posterior fixation T8-L2
Posterior fixation T8-L3

tested with additional pairwise Friedman test
without post-hoc correction

Segmental ROM lateral bending

Group 1

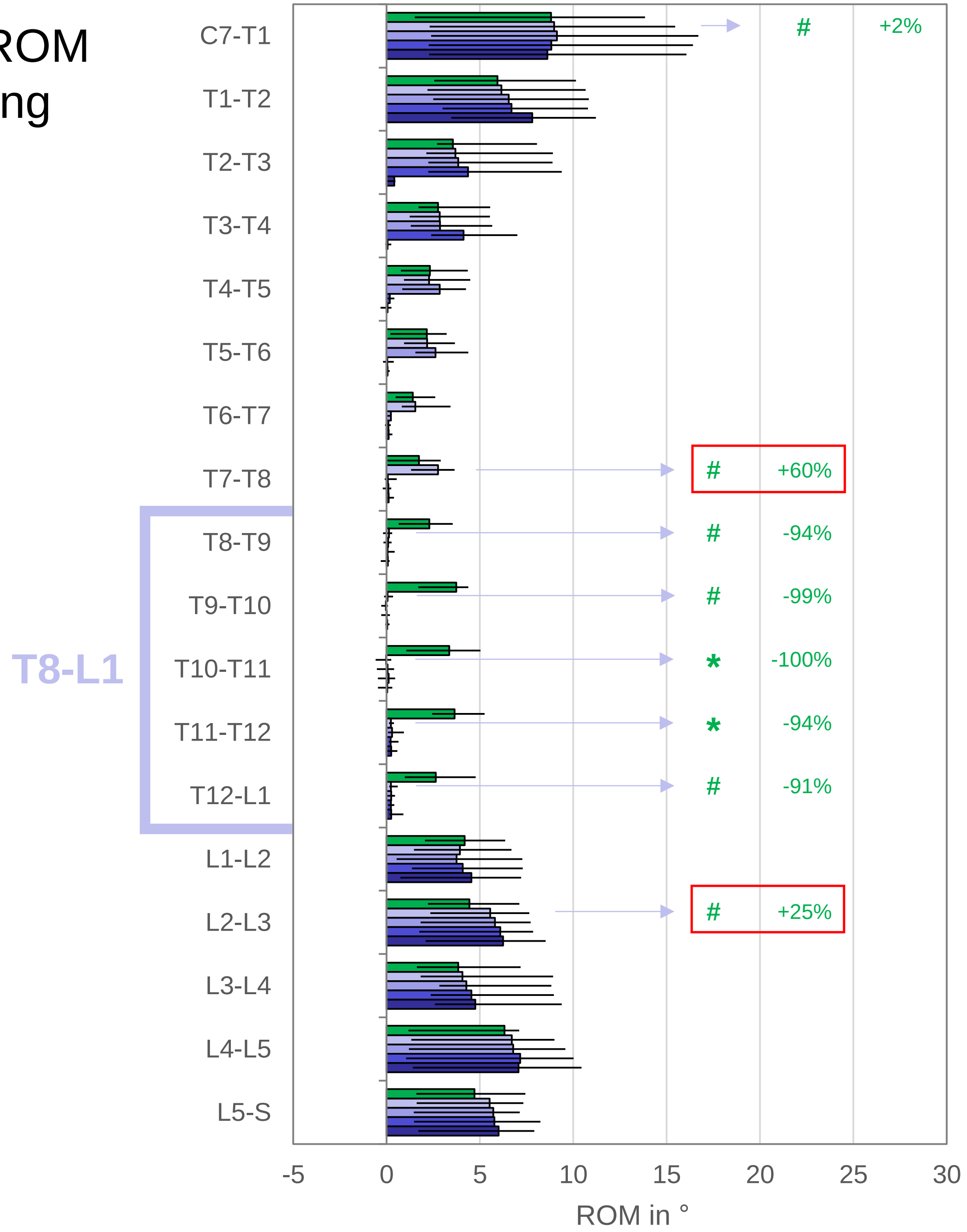

Effects of posterior fixation from T8 to L1

Without posterior fixation
Posterior fixation T8-L1
Posterior fixation T6-L1
Posterior fixation T4-L1
Posterior fixation T2-L1

Significant change ($p<0.05$) compared to

* Without posterior fixation
* Posterior fixation T8-L1
* Posterior fixation T6-L1
* Posterior fixation T4-L1

tested with Friedman's ANOVA
+ Bonferroni-Dunn post-hoc correction
+ pairwise comparisons

Without posterior fixation
Posterior fixation T8-L1
Posterior fixation T6-L1
Posterior fixation T4-L1

tested with additional pairwise Friedman test without post-hoc correction

Segmental ROM lateral bending

Group 1

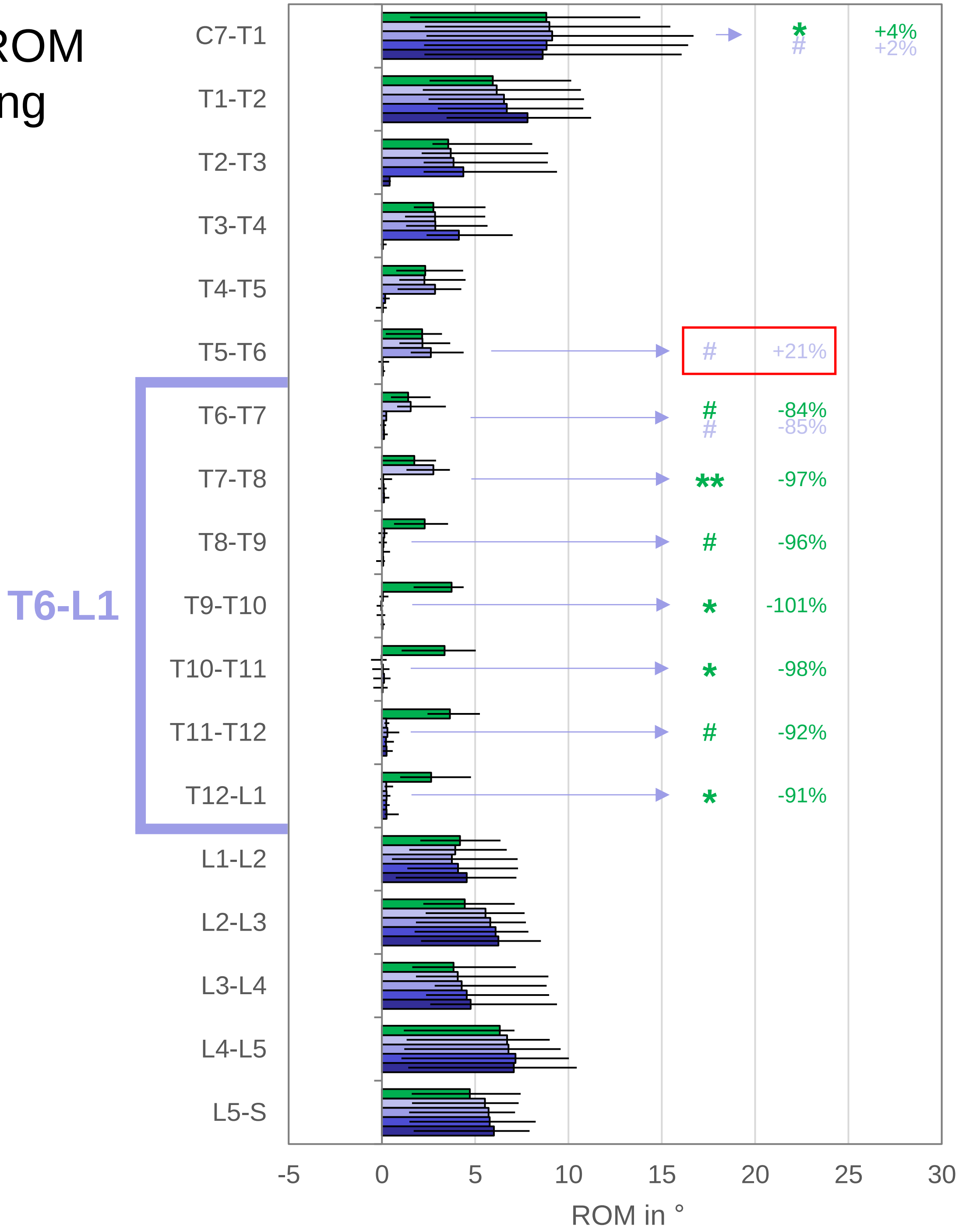

Effects of posterior fixation from T6 to L1

- Without posterior fixation
- Posterior fixation T8-L1
- Posterior fixation T6-L1
- Posterior fixation T4-L1
- Posterior fixation T2-L1

Significant change (p<0.05) compared to

* Without posterior fixation
* Posterior fixation T8-L1
* Posterior fixation T6-L1
* Posterior fixation T4-L1

tested with Friedman's ANOVA
+ Bonferroni-Dunn post-hoc correction
+ pairwise comparisons

Without posterior fixation
Posterior fixation T8-L1
Posterior fixation T6-L1
Posterior fixation T4-L1

tested with additional pairwise Friedman test
without post-hoc correction

Segmental ROM lateral bending

Group 1

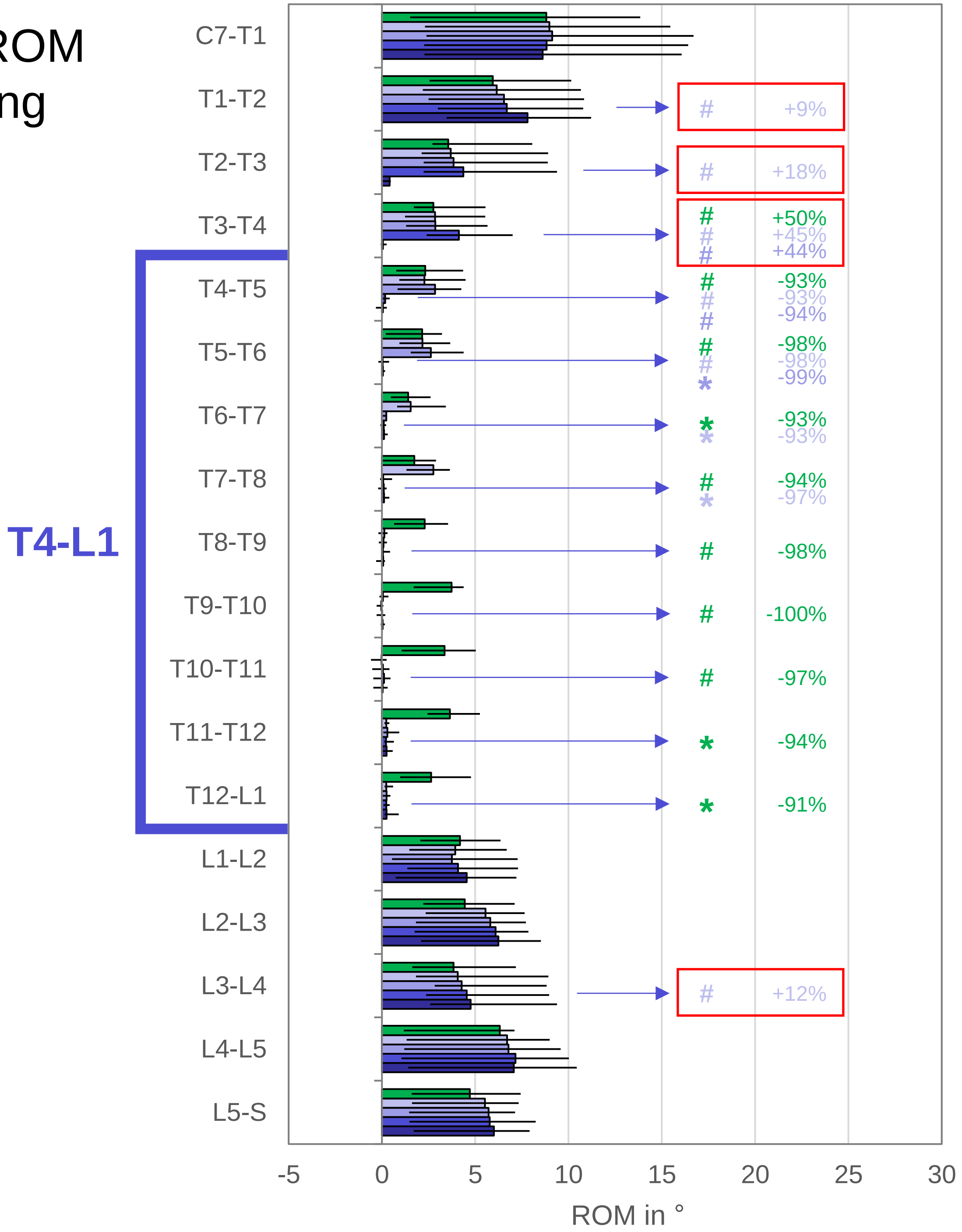

Effects of posterior fixation from T4 to L1

- Without posterior fixation
- Posterior fixation T8-L1
- Posterior fixation T6-L1
- Posterior fixation T4-L1
- Posterior fixation T2-L1

Significant change (p<0.05) compared to

* Without posterior fixation
* Posterior fixation T8-L1
* Posterior fixation T6-L1
* Posterior fixation T4-L1

tested with Friedman's ANOVA
+ Bonferroni-Dunn post-hoc correction
+ pairwise comparisons

Without posterior fixation
Posterior fixation T8-L1
Posterior fixation T6-L1
Posterior fixation T4-L1

tested with additional pairwise Friedman test
without post-hoc correction

Segmental ROM lateral bending

Group 1

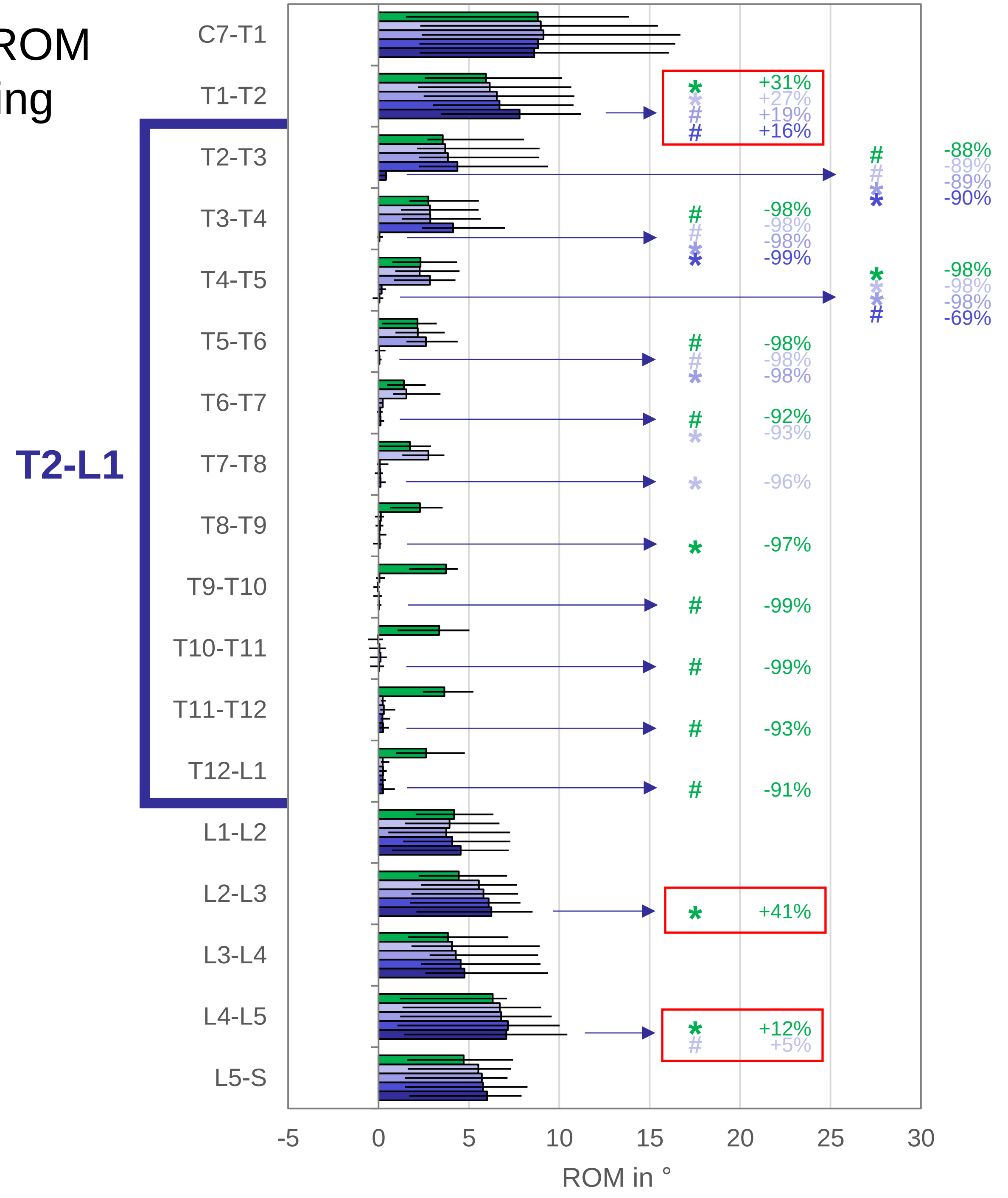

Effects of posterior fixation from T2 to L1

- Without posterior fixation
- Posterior fixation T8-L1
- Posterior fixation T6-L1
- Posterior fixation T4-L1
- Posterior fixation T2-L1

Significant change ($p<0.05$) compared to

* Without posterior fixation
* Posterior fixation T8-L1
* Posterior fixation T6-L1
* Posterior fixation T4-L1

tested with Friedman's ANOVA
+ Bonferroni-Dunn post-hoc correction
+ pairwise comparisons

Without posterior fixation
Posterior fixation T8-L1
Posterior fixation T6-L1
Posterior fixation T4-L1

tested with additional pairwise Friedman test without post-hoc correction

Segmental ROM lateral bending

Group 2

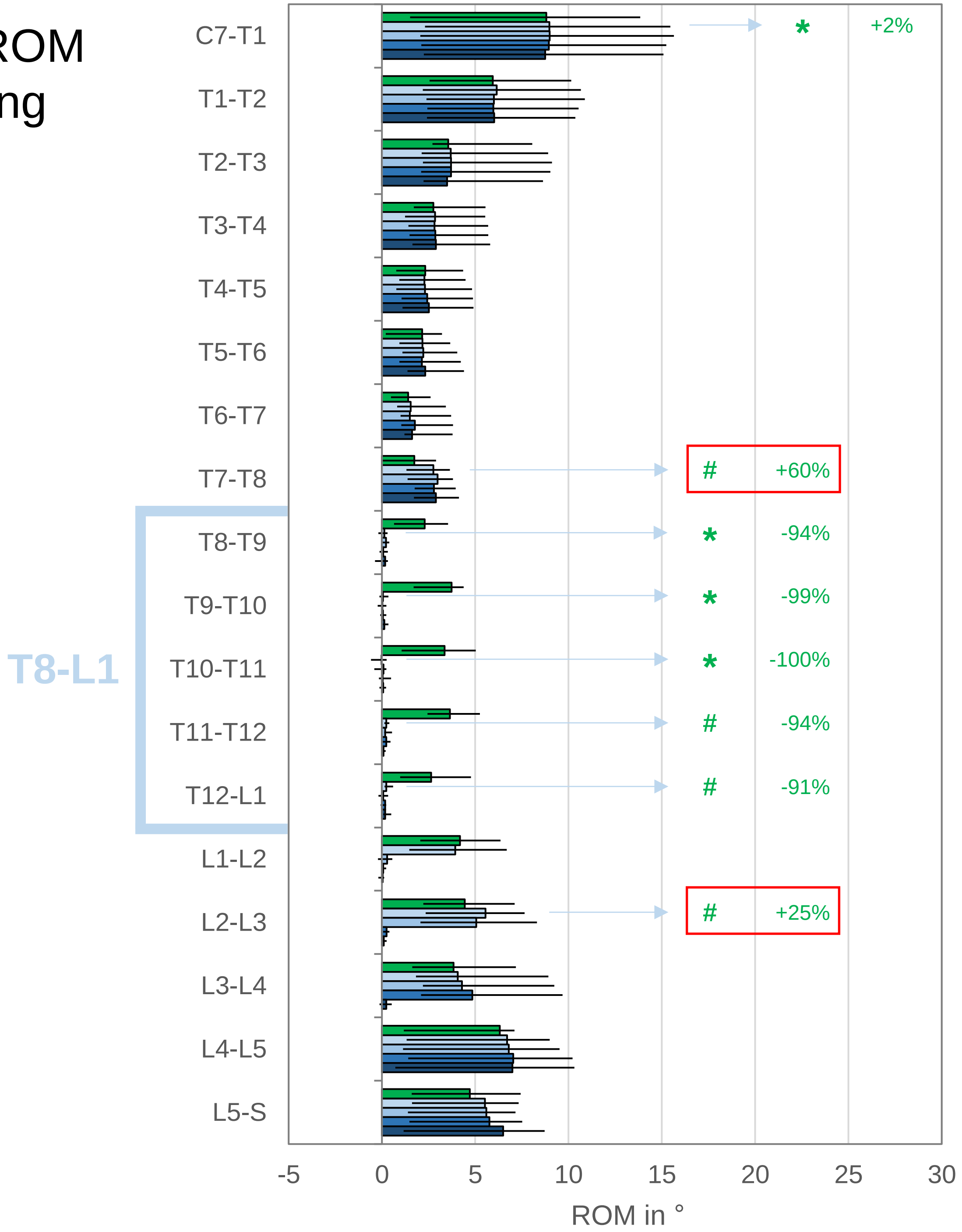

Effects of posterior fixation from T8 to L1

- Without posterior fixation
- Posterior fixation T8-L1
- Posterior fixation T8-L2
- Posterior fixation T8-L3
- Posterior fixation T8-L4

Significant change ($p<0.05$) compared to

* Without posterior fixation
* Posterior fixation T8-L1
* Posterior fixation T8-L2
* Posterior fixation T8-L3

tested with Friedman's ANOVA
+ Bonferroni-Dunn post-hoc correction
+ pairwise comparisons

Without posterior fixation
Posterior fixation T8-L1
Posterior fixation T8-L2
Posterior fixation T8-L3

tested with additional pairwise Friedman test
without post-hoc correction

Segmental ROM lateral bending

Group 2

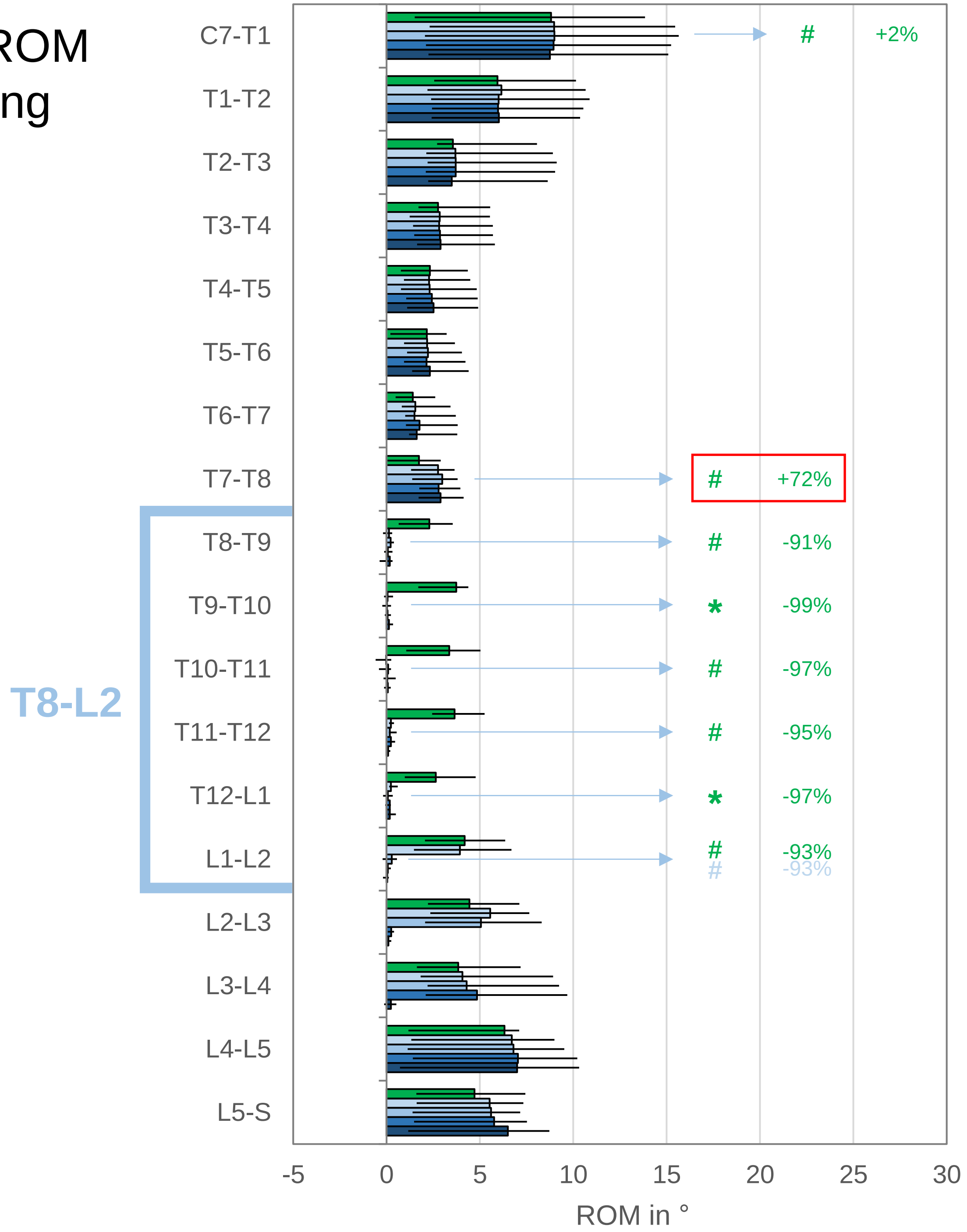

Effects of posterior fixation from T8 to L2

- Without posterior fixation
- Posterior fixation T8-L1
- Posterior fixation T8-L2
- Posterior fixation T8-L3
- Posterior fixation T8-L4

Significant change (p<0.05) compared to

* Without posterior fixation
* Posterior fixation T8-L1
* Posterior fixation T8-L2
* Posterior fixation T8-L3

tested with Friedman's ANOVA
+ Bonferroni-Dunn post-hoc correction
+ pairwise comparisons

Without posterior fixation
Posterior fixation T8-L1
Posterior fixation T8-L2
Posterior fixation T8-L3

tested with additional pairwise Friedman test
without post-hoc correction

Segmental ROM lateral bending

Group 2

Effects of posterior fixation from T8 to L3

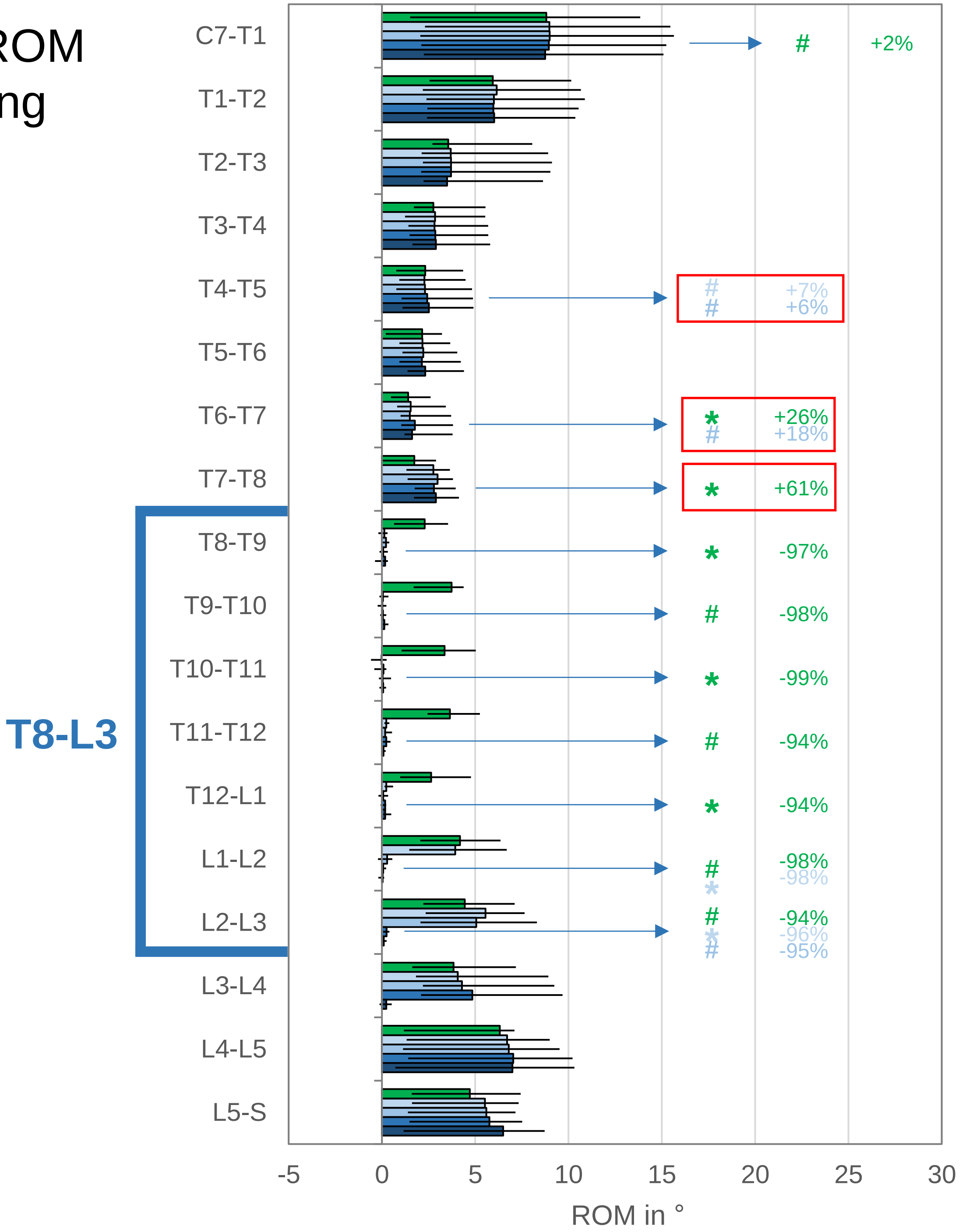

- Without posterior fixation
- Posterior fixation T8-L1
- Posterior fixation T8-L2
- Posterior fixation T8-L3
- Posterior fixation T8-L4

Significant change (p<0.05) compared to

- * Without posterior fixation
- * Posterior fixation T8-L1
- * Posterior fixation T8-L2
- * Posterior fixation T8-L3

tested with Friedman's ANOVA
+ Bonferroni-Dunn post-hoc correction
+ pairwise comparisons

- # Without posterior fixation
- # Posterior fixation T8-L1
- # Posterior fixation T8-L2
- # Posterior fixation T8-L3

tested with additional pairwise Friedman test without post-hoc correction

Segmental ROM lateral bending

Group 2

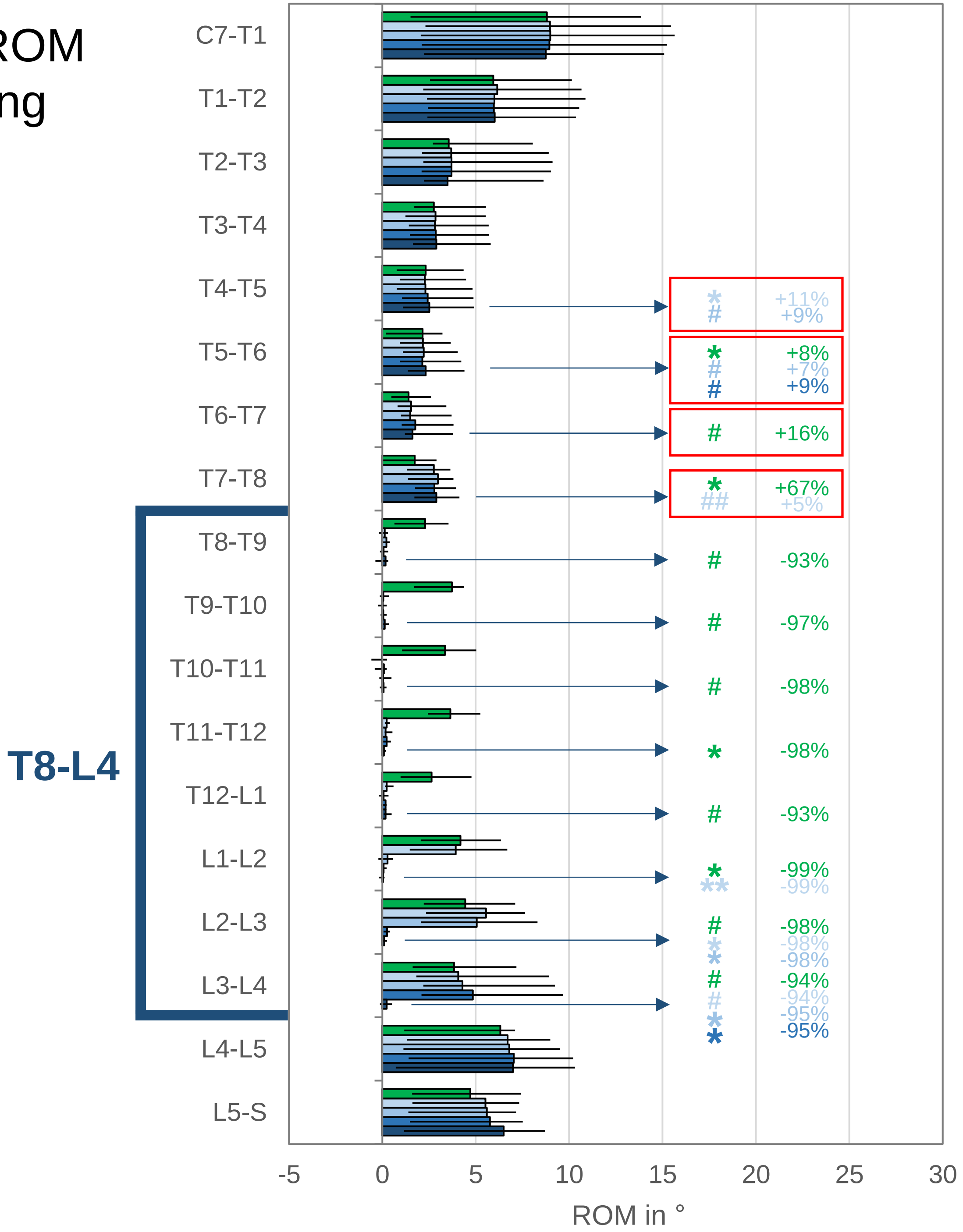

Effects of posterior fixation from T8 to L4

- Without posterior fixation
- Posterior fixation T8-L1
- Posterior fixation T8-L2
- Posterior fixation T8-L3
- Posterior fixation T8-L4

Significant change (p<0.05) compared to

* Without posterior fixation
* Posterior fixation T8-L1
* Posterior fixation T8-L2
* Posterior fixation T8-L3

tested with Friedman's ANOVA
+ Bonferroni-Dunn post-hoc correction
+ pairwise comparisons

Without posterior fixation
Posterior fixation T8-L1
Posterior fixation T8-L2
Posterior fixation T8-L3

tested with additional pairwise Friedman test
without post-hoc correction

Segmental ROM
axial rotation

Group 1

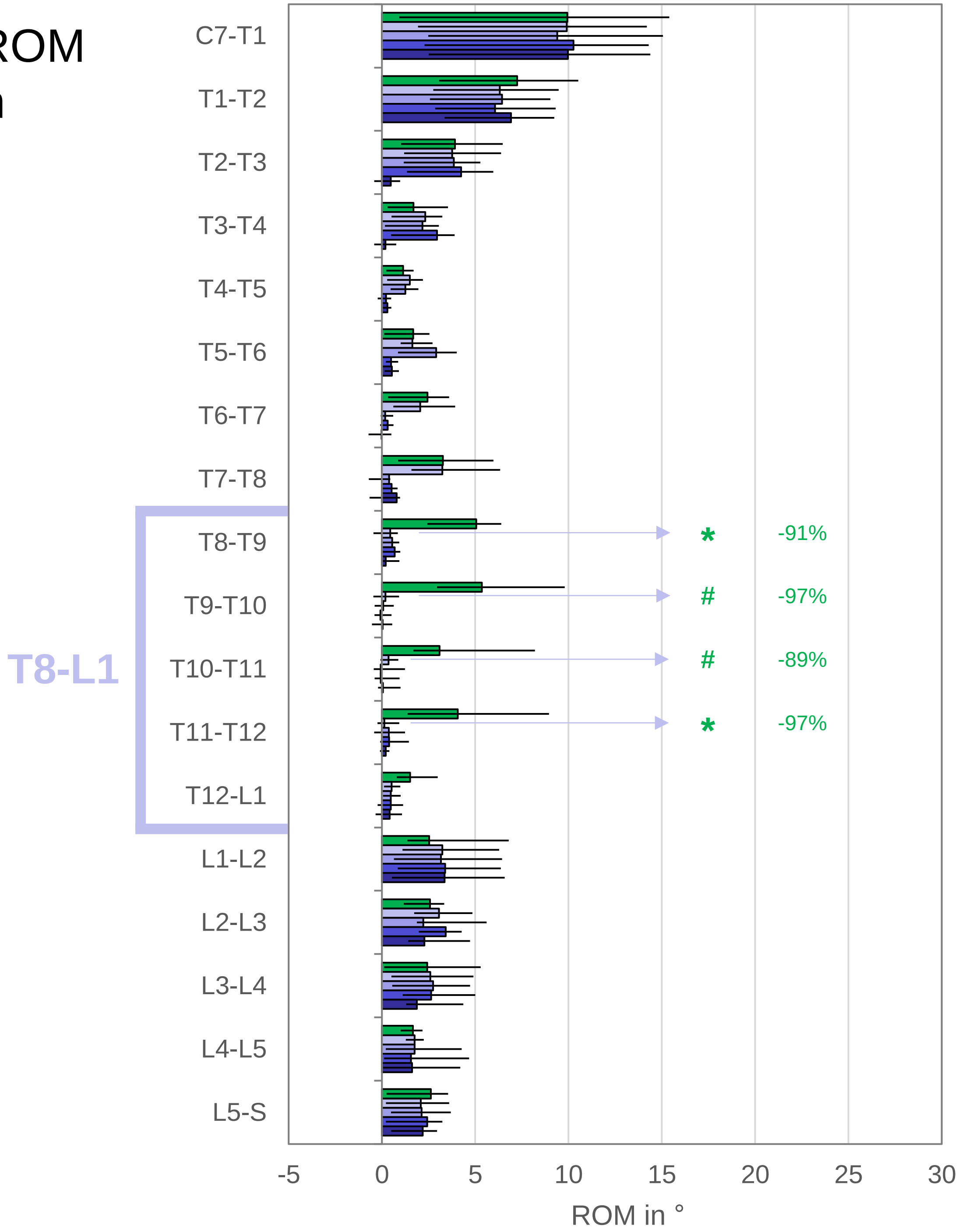

Effects of posterior fixation from T8 to L1

- Without posterior fixation
- Posterior fixation T8-L1
- Posterior fixation T6-L1
- Posterior fixation T4-L1
- Posterior fixation T2-L1

Significant change ($p<0.05$) compared to

* Without posterior fixation
* Posterior fixation T8-L1
* Posterior fixation T6-L1
* Posterior fixation T4-L1

tested with Friedman's ANOVA
+ Bonferroni-Dunn post-hoc correction
+ pairwise comparisons

Without posterior fixation
Posterior fixation T8-L1
Posterior fixation T6-L1
Posterior fixation T4-L1

tested with additional pairwise Friedman test
without post-hoc correction

Segmental ROM axial rotation

Group 1

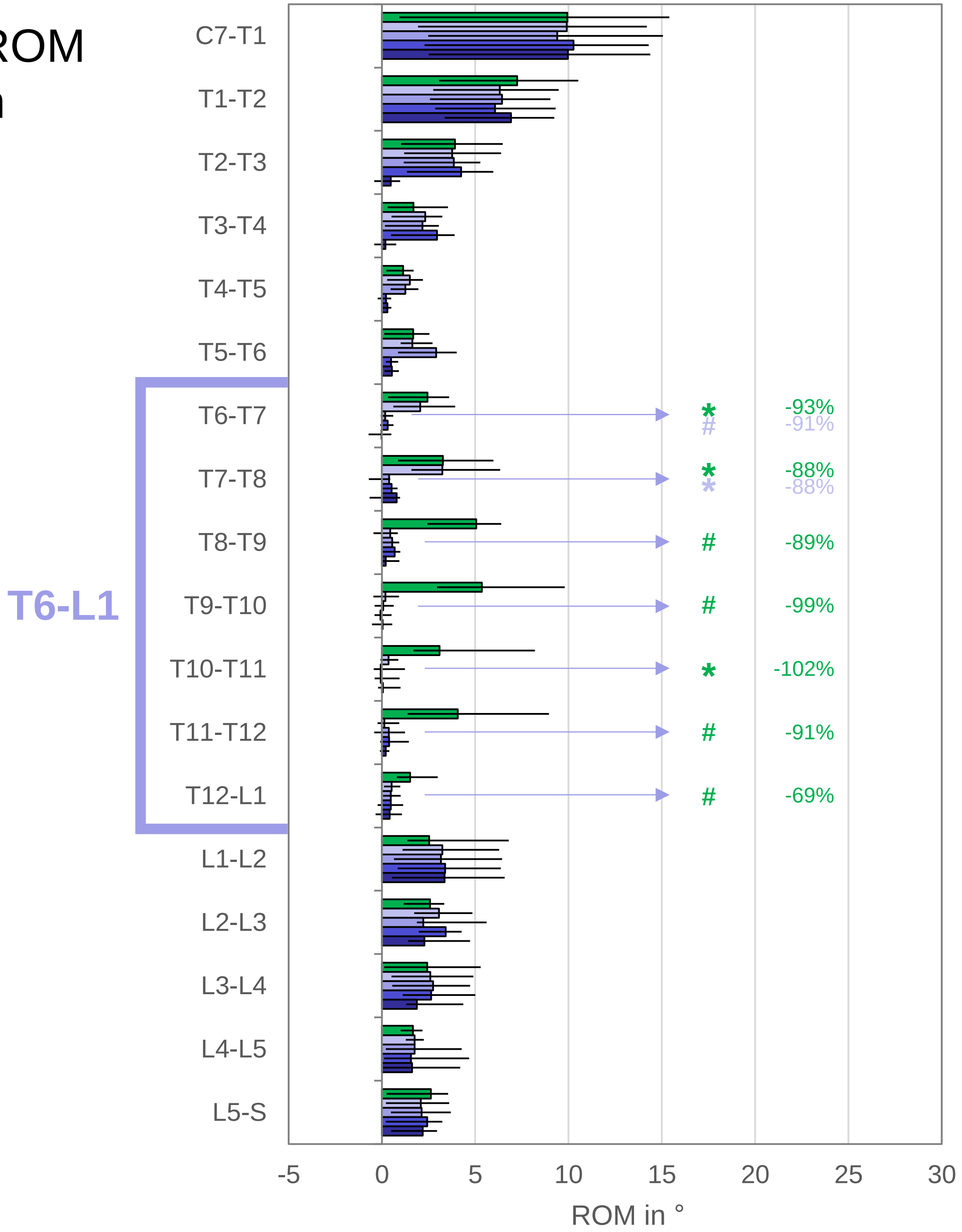

Effects of posterior fixation from T6 to L1

- Without posterior fixation
- Posterior fixation T8-L1
- Posterior fixation T6-L1
- Posterior fixation T4-L1
- Posterior fixation T2-L1

Significant change (p<0.05) compared to

* Without posterior fixation
* Posterior fixation T8-L1
* Posterior fixation T6-L1
* Posterior fixation T4-L1

tested with Friedman's ANOVA
+ Bonferroni-Dunn post-hoc correction
+ pairwise comparisons

Without posterior fixation
Posterior fixation T8-L1
Posterior fixation T6-L1
Posterior fixation T4-L1

tested with additional pairwise Friedman test without post-hoc correction

Segmental ROM axial rotation

Group 1

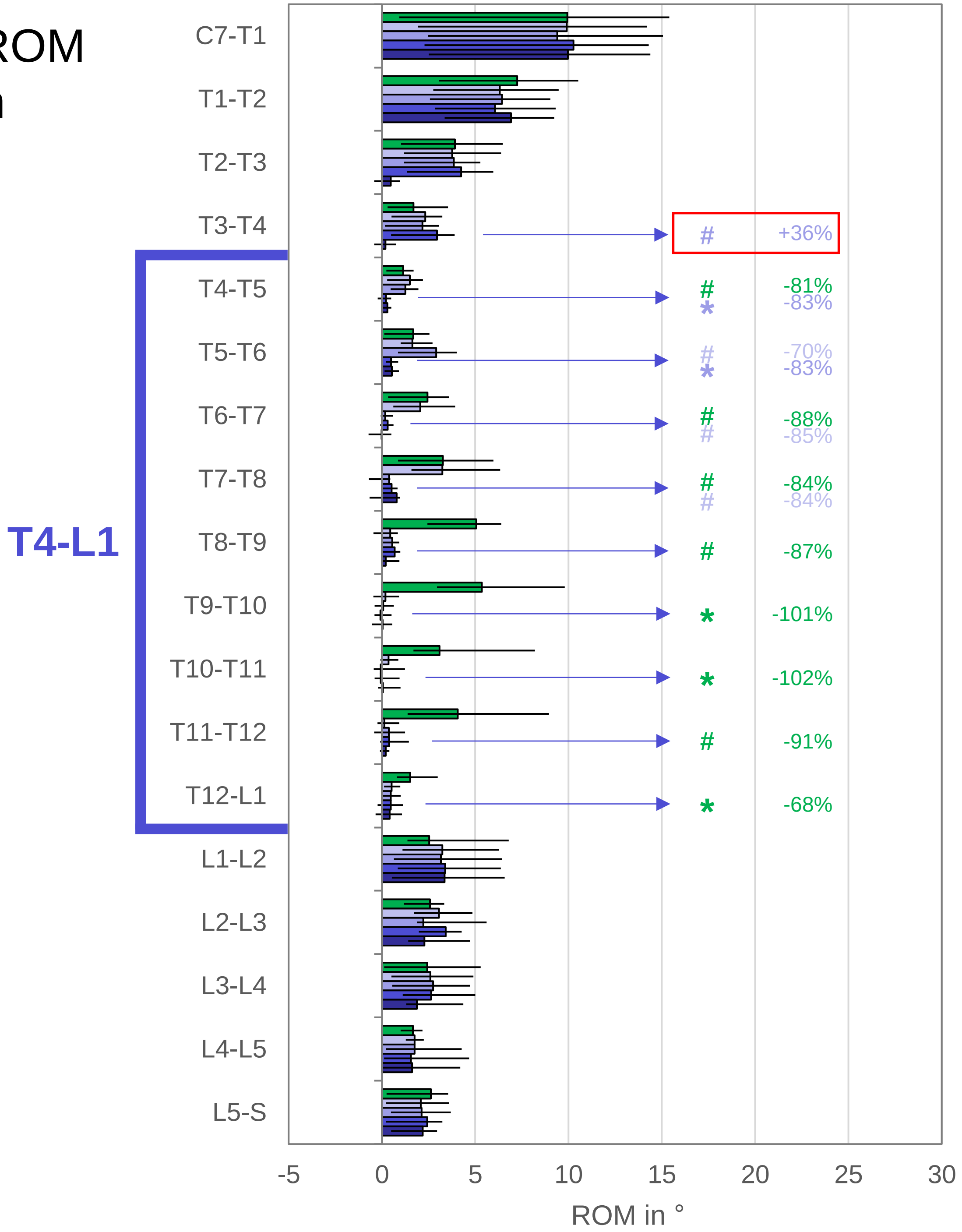

Effects of posterior fixation from T4 to L1

- Without posterior fixation
- Posterior fixation T8-L1
- Posterior fixation T6-L1
- Posterior fixation T4-L1
- Posterior fixation T2-L1

Significant change ($p<0.05$) compared to

* Without posterior fixation
* Posterior fixation T8-L1
* Posterior fixation T6-L1
* Posterior fixation T4-L1

tested with Friedman's ANOVA
+ Bonferroni-Dunn post-hoc correction
+ pairwise comparisons

Without posterior fixation
Posterior fixation T8-L1
Posterior fixation T6-L1
Posterior fixation T4-L1

tested with additional pairwise Friedman test
without post-hoc correction

Segmental ROM axial rotation

Group 1

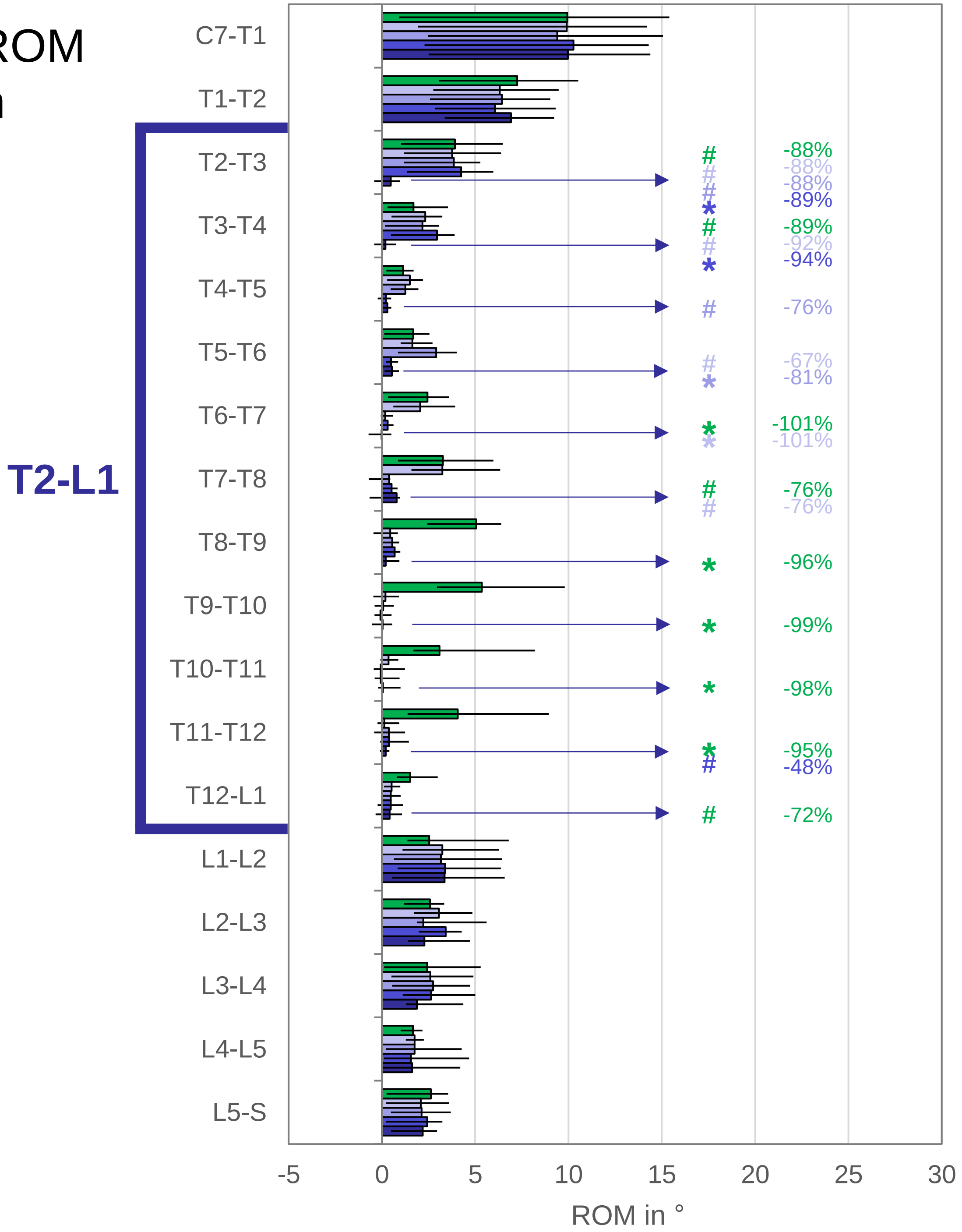

Effects of posterior fixation from T2 to L1

- Without posterior fixation
- Posterior fixation T8-L1
- Posterior fixation T6-L1
- Posterior fixation T4-L1
- Posterior fixation T2-L1

Significant change (p<0.05) compared to

* Without posterior fixation
* Posterior fixation T8-L1
* Posterior fixation T6-L1
* Posterior fixation T4-L1

tested with Friedman's ANOVA
+ Bonferroni-Dunn post-hoc correction
+ pairwise comparisons

Without posterior fixation
Posterior fixation T8-L1
Posterior fixation T6-L1
Posterior fixation T4-L1

tested with additional pairwise Friedman test without post-hoc correction

Segmental ROM axial rotation

Group 2

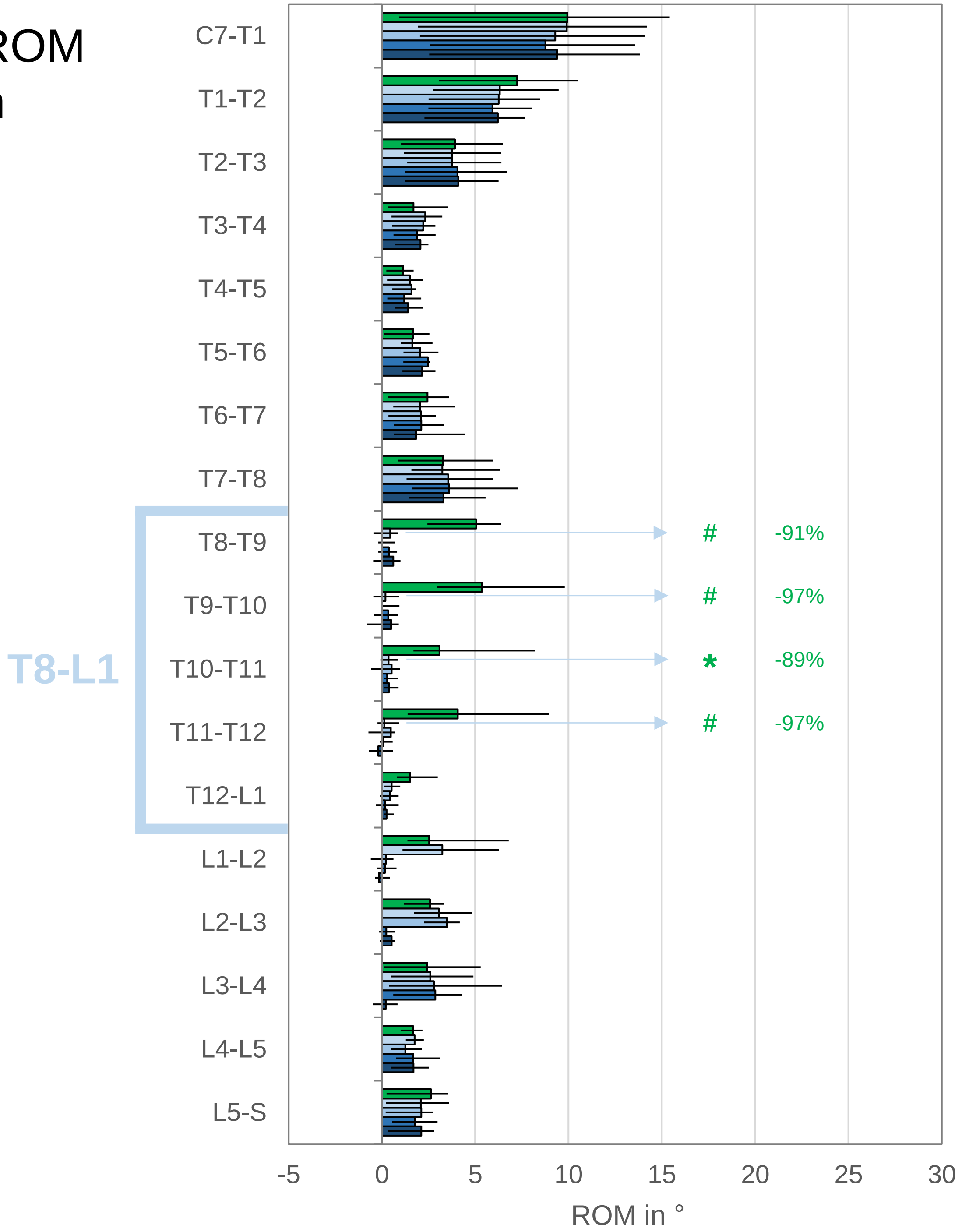

Effects of posterior fixation from T8 to L1

- Without posterior fixation
- Posterior fixation T8-L1
- Posterior fixation T8-L2
- Posterior fixation T8-L3
- Posterior fixation T8-L4

Significant change (p<0.05) compared to

* Without posterior fixation
* Posterior fixation T8-L1
* Posterior fixation T8-L2
* Posterior fixation T8-L3

tested with Friedman's ANOVA
+ Bonferroni-Dunn post-hoc correction
+ pairwise comparisons

Without posterior fixation
Posterior fixation T8-L1
Posterior fixation T8-L2
Posterior fixation T8-L3

tested with additional pairwise Friedman test without post-hoc correction

Segmental ROM axial rotation

Group 2

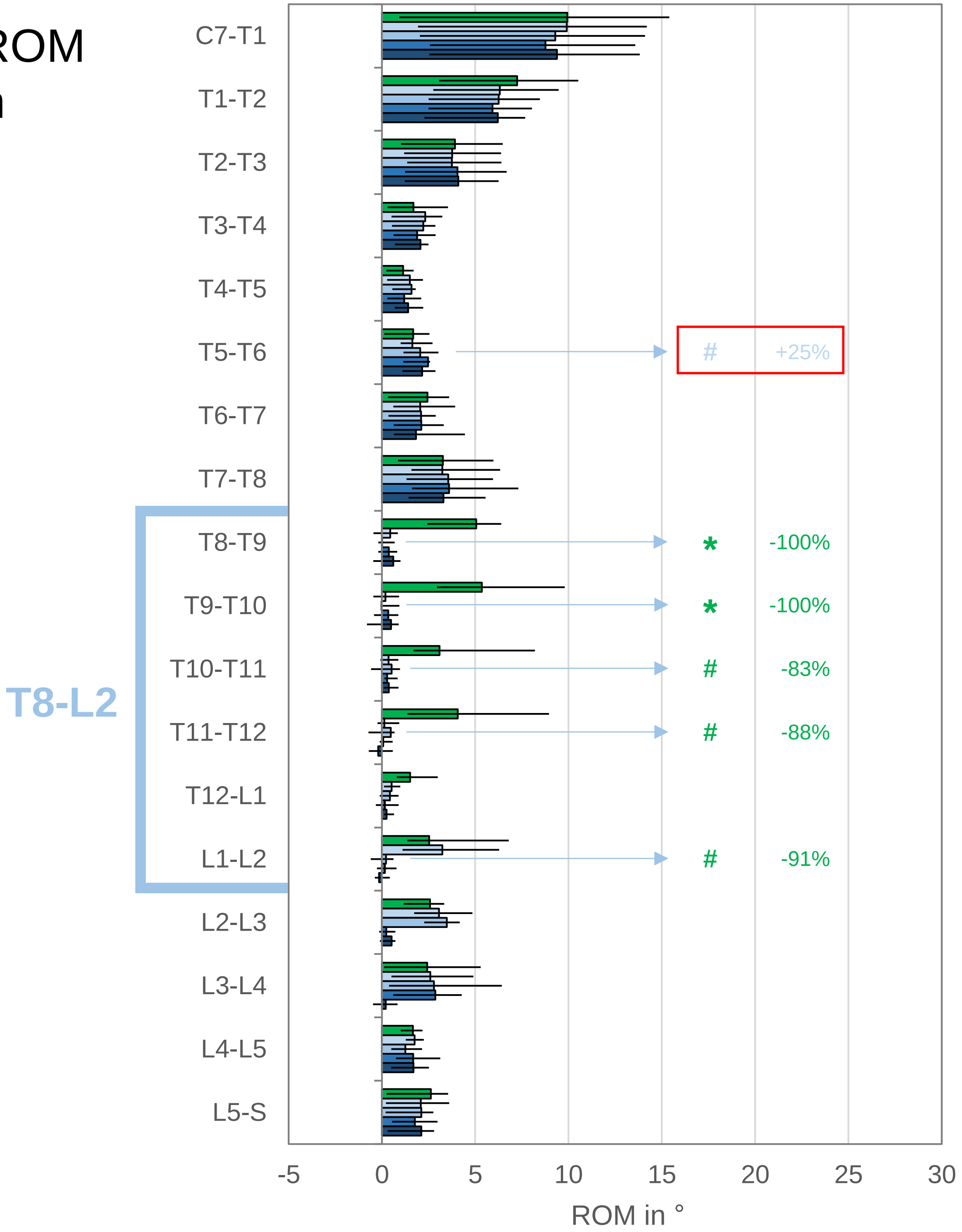

Effects of posterior fixation from T8 to L2

- Without posterior fixation
- Posterior fixation T8-L1
- Posterior fixation T8-L2
- Posterior fixation T8-L3
- Posterior fixation T8-L4

Significant change (p<0.05) compared to

* Without posterior fixation
* Posterior fixation T8-L1
* Posterior fixation T8-L2
* Posterior fixation T8-L3

tested with Friedman's ANOVA
+ Bonferroni-Dunn post-hoc correction
+ pairwise comparisons

Without posterior fixation
Posterior fixation T8-L1
Posterior fixation T8-L2
Posterior fixation T8-L3

tested with additional pairwise Friedman test without post-hoc correction

Segmental ROM
axial rotation

Group 2

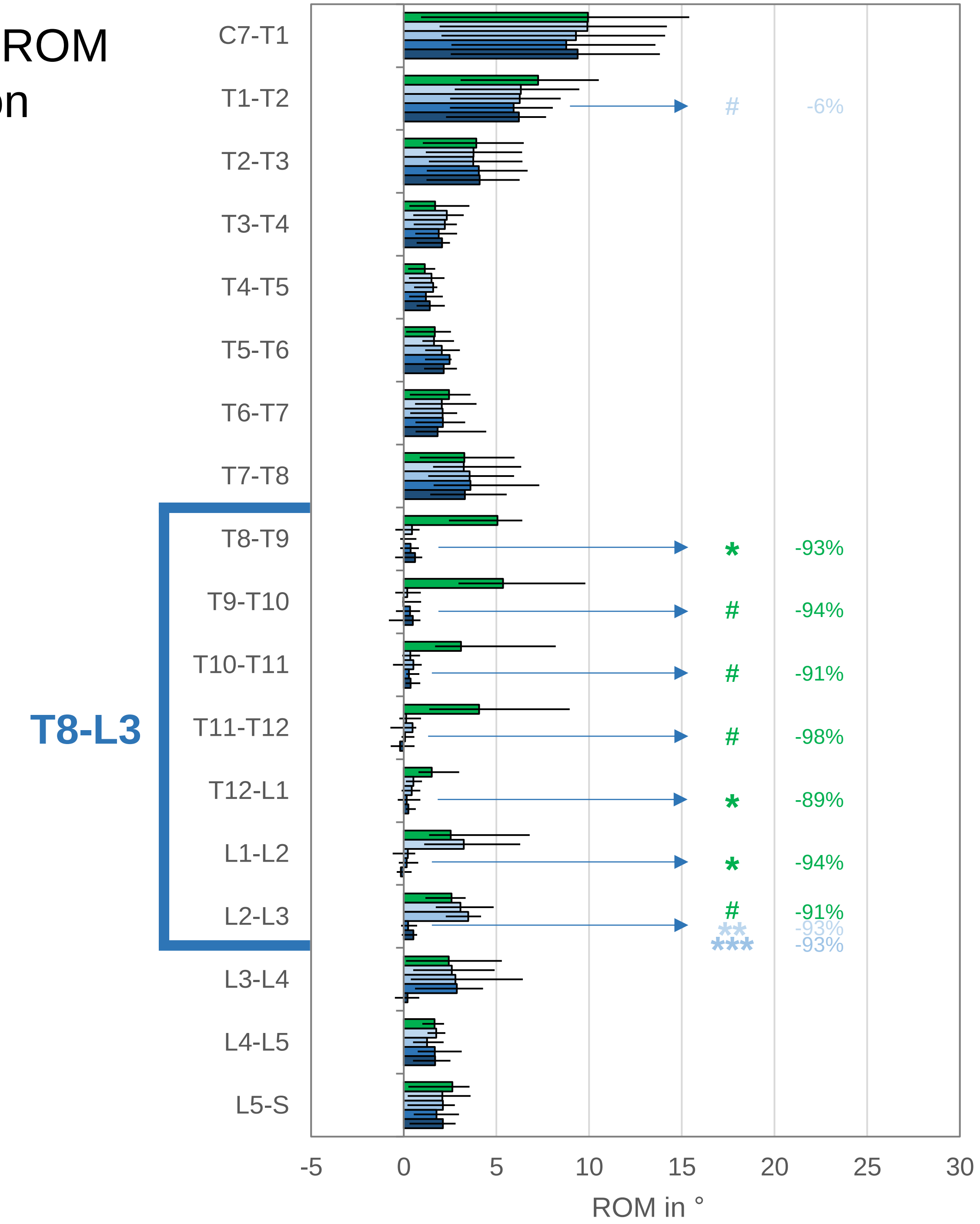

Effects of posterior fixation from T8 to L3

- Without posterior fixation
- Posterior fixation T8-L1
- Posterior fixation T8-L2
- Posterior fixation T8-L3
- Posterior fixation T8-L4

Significant change ($p<0.05$) compared to

* Without posterior fixation
* Posterior fixation T8-L1
* Posterior fixation T8-L2
* Posterior fixation T8-L3
tested with Friedman's ANOVA
+ Bonferroni-Dunn post-hoc correction
+ pairwise comparisons

Without posterior fixation
Posterior fixation T8-L1
Posterior fixation T8-L2
Posterior fixation T8-L3
tested with additional pairwise Friedman test
without post-hoc correction

Segmental ROM axial rotation

Group 2

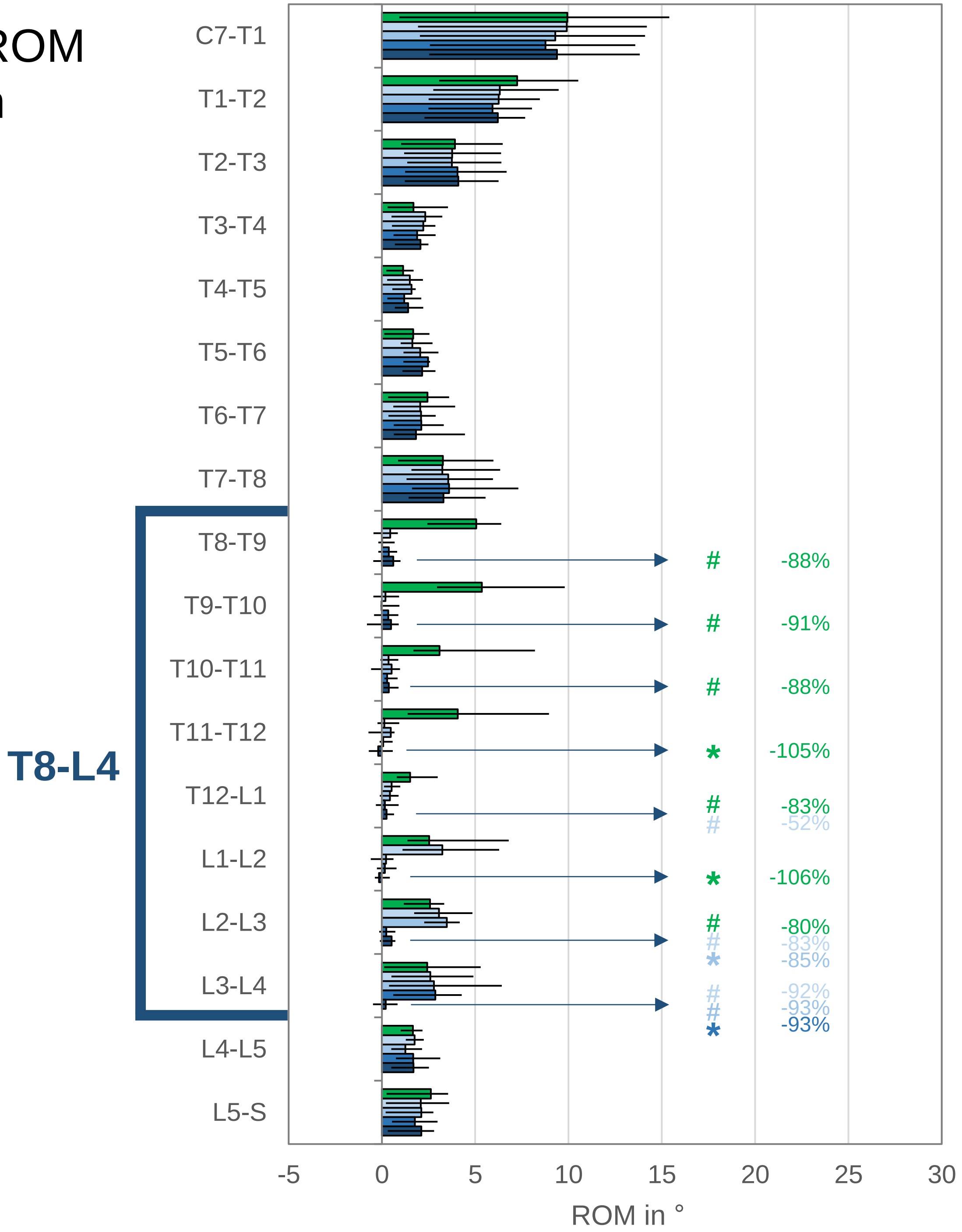

Effects of posterior fixation from T8 to L4

- Without posterior fixation
- Posterior fixation T8-L1
- Posterior fixation T8-L2
- Posterior fixation T8-L3
- Posterior fixation T8-L4

Significant change (p<0.05) compared to

* Without posterior fixation
* Posterior fixation T8-L1
* Posterior fixation T8-L2
* Posterior fixation T8-L3

tested with Friedman's ANOVA
+ Bonferroni-Dunn post-hoc correction
+ pairwise comparisons

Without posterior fixation
Posterior fixation T8-L1
Posterior fixation T8-L2
Posterior fixation T8-L3

tested with additional pairwise Friedman test
without post-hoc correction

Intrinsic IDP

Group 1: Increasing fixation length in upward direction

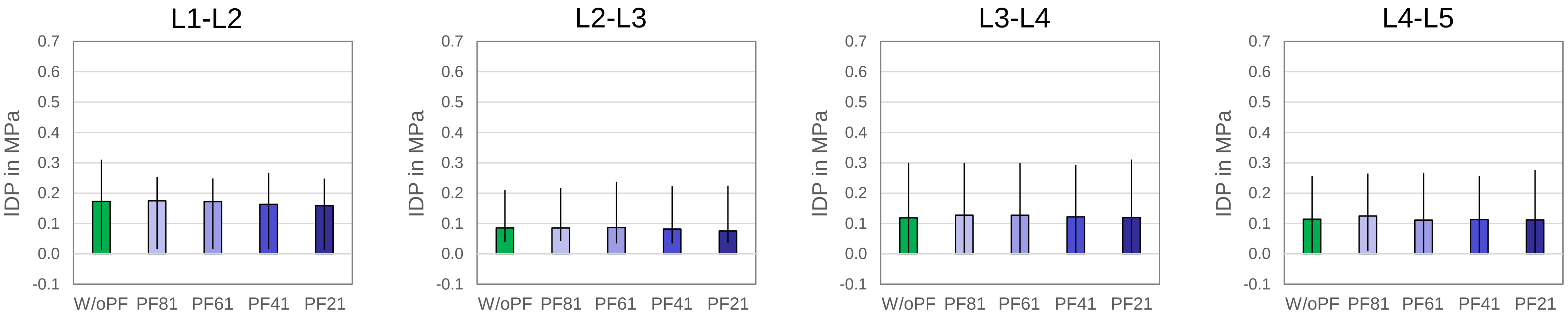

- W/oPF = Without posterior fixation
- PF81 = Posterior fixation T8-L1
- PF61 = Posterior fixation T6-L1
- PF41 = Posterior fixation T4-L1
- PF21 = Posterior fixation T2-L1

Significant change ($p<0.05$) compared to

* Without posterior fixation	# Without posterior fixation
* Posterior fixation T8-L1	# Posterior fixation T8-L1
* Posterior fixation T6-L1	# Posterior fixation T6-L1
* Posterior fixation T4-L1	# Posterior fixation T4-L1
tested with Friedman's ANOVA<br>+ Bonferroni-Dunn post-hoc correction<br>+ pairwise comparisons	tested with additional pairwise Friedman test<br>without post-hoc correction

Intrinsic IDP

Group 2: Increasing fixation length in downward direction

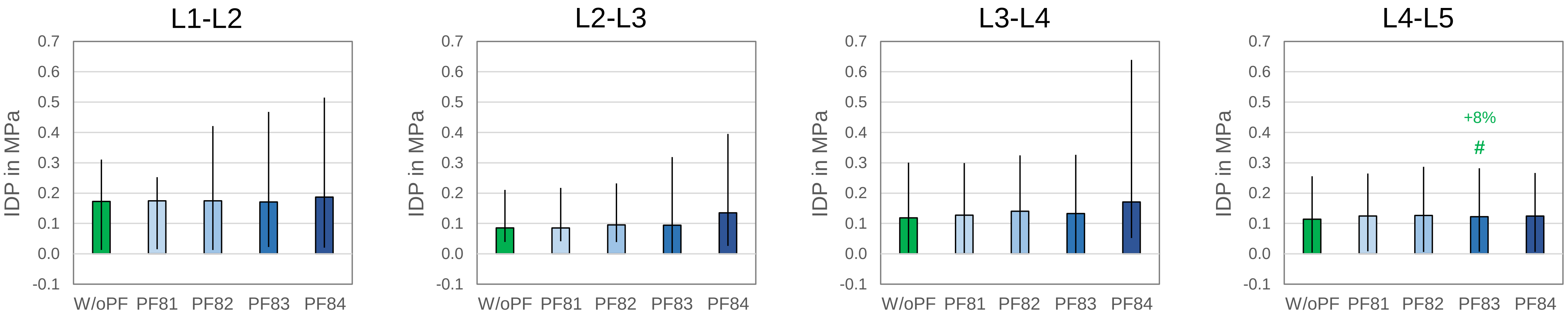

- W/oPF = Without posterior fixation
- PF81 = Posterior fixation T8-L1
- PF82 = Posterior fixation T8-L2
- PF83 = Posterior fixation T8-L3
- PF84 = Posterior fixation T8-L4

Significant change ($p<0.05$) compared to

* Without posterior fixation	# Without posterior fixation
* Posterior fixation T8-L1	# Posterior fixation T8-L1
* Posterior fixation T8-L2	# Posterior fixation T8-L2
* Posterior fixation T8-L3	# Posterior fixation T8-L3
tested with Friedman's ANOVA<br>+ Bonferroni-Dunn post-hoc correction<br>+ pairwise comparisons	tested with additional pairwise Friedman test<br>without post-hoc correction

IDP Flexion

Group 1: Increasing fixation length in upward direction

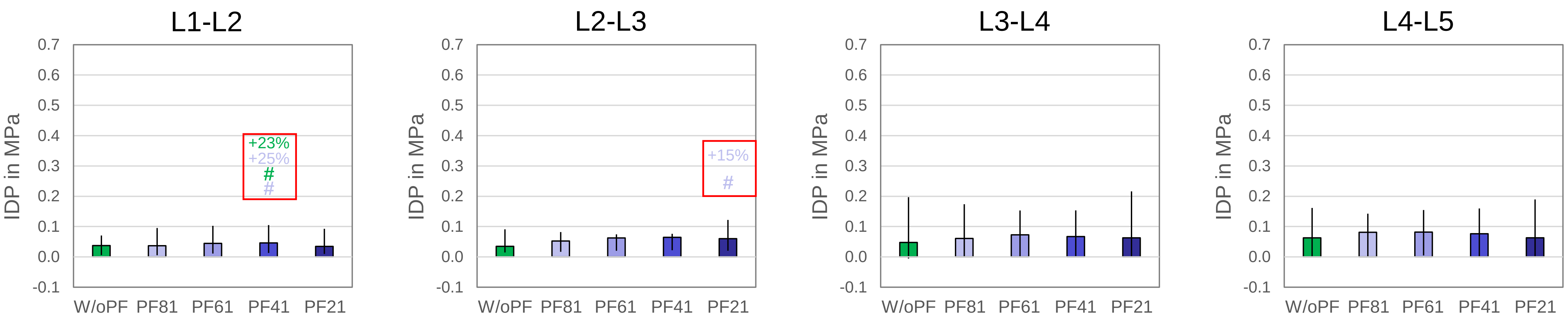

- W/oPF = Without posterior fixation
- PF81 = Posterior fixation T8-L1
- PF61 = Posterior fixation T6-L1
- PF41 = Posterior fixation T4-L1
- PF21 = Posterior fixation T2-L1

Significant change ($p<0.05$) compared to

* Without posterior fixation
* Posterior fixation T8-L1
* Posterior fixation T6-L1
* Posterior fixation T4-L1

tested with Friedman's ANOVA
+ Bonferroni-Dunn post-hoc correction
+ pairwise comparisons

Without posterior fixation
Posterior fixation T8-L1
Posterior fixation T6-L1
Posterior fixation T4-L1

tested with additional pairwise Friedman test
without post-hoc correction

IDP Flexion

Group 2: Increasing fixation length in downward direction

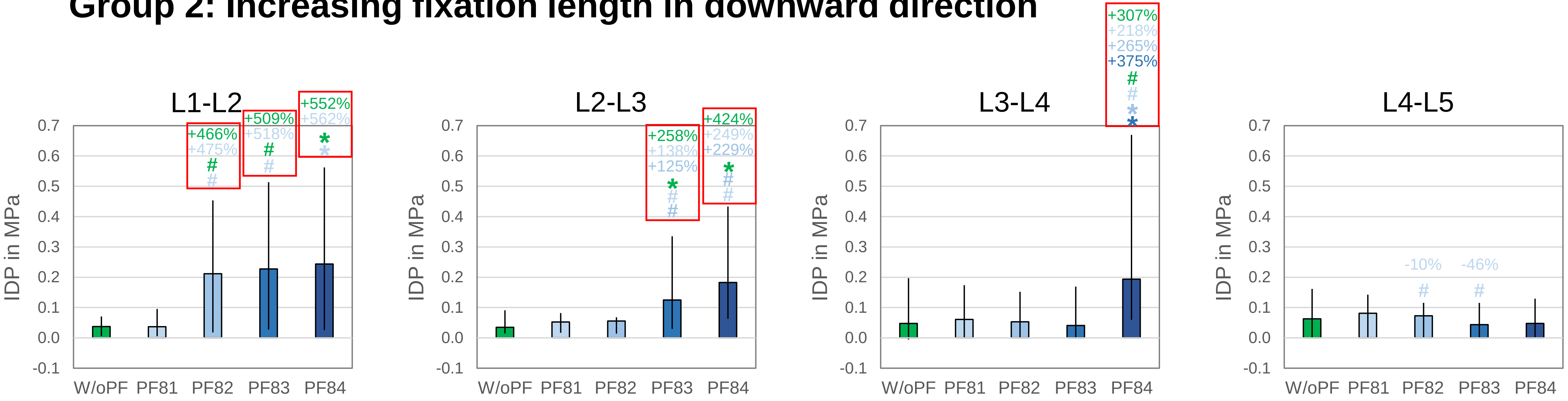

W/oPF = Without posterior fixation
PF81 = Posterior fixation T8-L1
PF82 = Posterior fixation T8-L2
PF83 = Posterior fixation T8-L3
PF84 = Posterior fixation T8-L4

Significant change ($p<0.05$) compared to

* Without posterior fixation	# Without posterior fixation
* Posterior fixation T8-L1	# Posterior fixation T8-L1
* Posterior fixation T8-L2	# Posterior fixation T8-L2
* Posterior fixation T8-L3	# Posterior fixation T8-L3
tested with Friedman's ANOVA<br>+ Bonferroni-Dunn post-hoc correction<br>+ pairwise comparisons	tested with additional pairwise Friedman test<br>without post-hoc correction

IDP Extension

Group 1: Increasing fixation length in upward direction

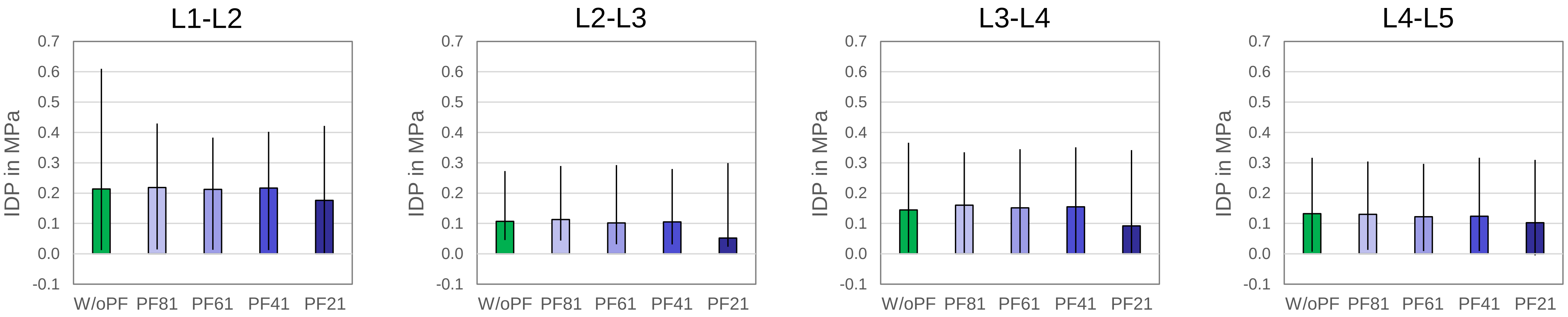

- W/oPF = Without posterior fixation
- PF81 = Posterior fixation T8-L1
- PF61 = Posterior fixation T6-L1
- PF41 = Posterior fixation T4-L1
- PF21 = Posterior fixation T2-L1

Significant change ($p<0.05$) compared to

* Without posterior fixation	# Without posterior fixation
* Posterior fixation T8-L1	# Posterior fixation T8-L1
* Posterior fixation T6-L1	# Posterior fixation T6-L1
* Posterior fixation T4-L1	# Posterior fixation T4-L1
tested with Friedman's ANOVA + Bonferroni-Dunn post-hoc correction + pairwise comparisons	tested with additional pairwise Friedman test without post-hoc correction

IDP Extension

Group 2: Increasing fixation length in downward direction

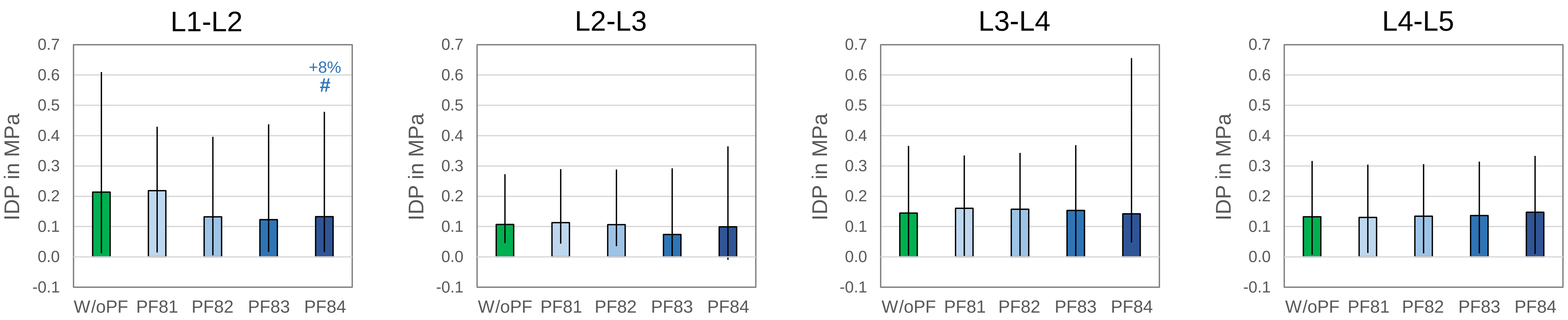

- W/oPF = Without posterior fixation
- PF81 = Posterior fixation T8-L1
- PF82 = Posterior fixation T8-L2
- PF83 = Posterior fixation T8-L3
- PF84 = Posterior fixation T8-L4

Significant change (p<0.05) compared to

* Without posterior fixation	# Without posterior fixation
* Posterior fixation T8-L1	# Posterior fixation T8-L1
* Posterior fixation T8-L2	# Posterior fixation T8-L2
* Posterior fixation T8-L3	# Posterior fixation T8-L3
tested with Friedman's ANOVA + Bonferroni-Dunn post-hoc correction + pairwise comparisons	tested with additional pairwise Friedman test without post-hoc correction

IDP Left lateral bending

Group 1: Increasing fixation length in upward direction

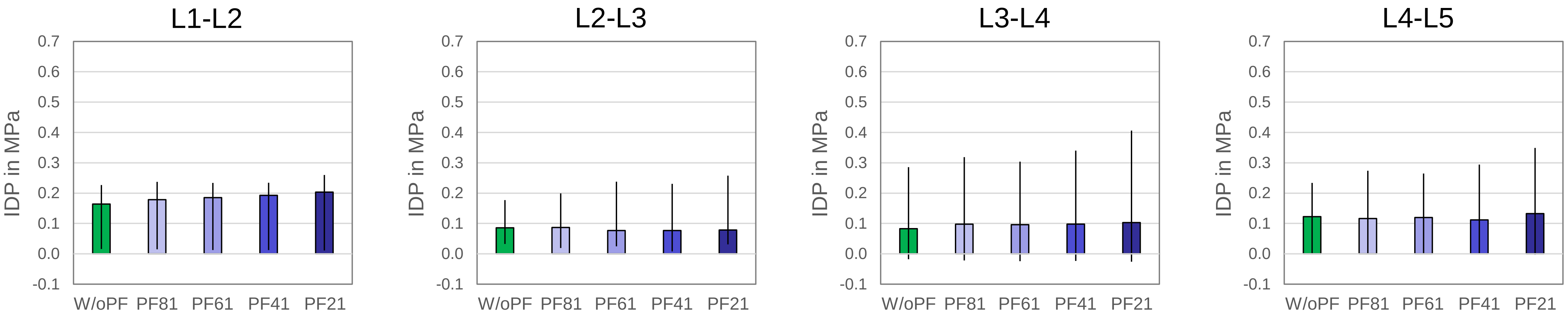

- W/oPF = Without posterior fixation
- PF81 = Posterior fixation T8-L1
- PF61 = Posterior fixation T6-L1
- PF41 = Posterior fixation T4-L1
- PF21 = Posterior fixation T2-L1

Significant change ($p<0.05$) compared to

* Without posterior fixation	# Without posterior fixation
* Posterior fixation T8-L1	# Posterior fixation T8-L1
* Posterior fixation T6-L1	# Posterior fixation T6-L1
* Posterior fixation T4-L1	# Posterior fixation T4-L1
tested with Friedman's ANOVA<br>+ Bonferroni-Dunn post-hoc correction<br>+ pairwise comparisons	tested with additional pairwise Friedman test<br>without post-hoc correction

IDP Left lateral bending

Group 2: Increasing fixation length in downward direction

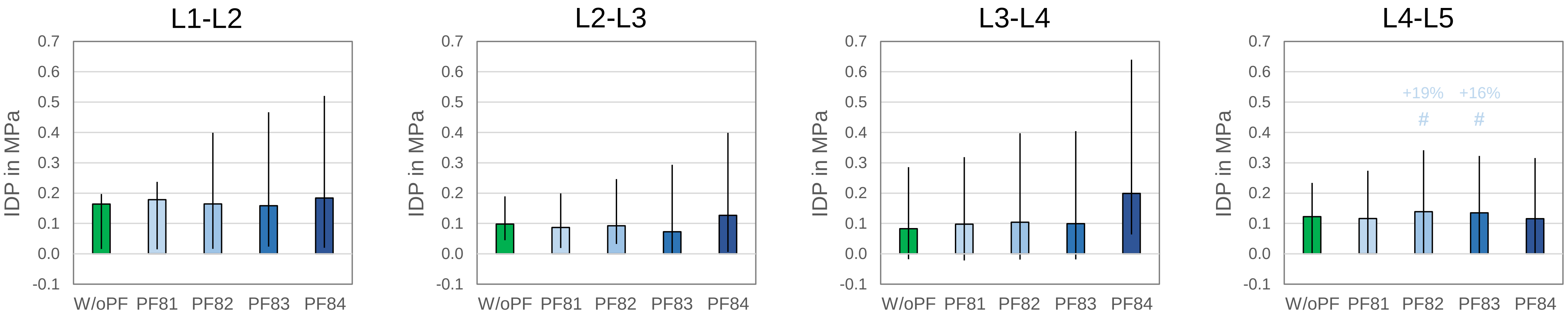

W/oPF = Without posterior fixation
PF81 = Posterior fixation T8-L1
PF82 = Posterior fixation T8-L2
PF83 = Posterior fixation T8-L3
PF84 = Posterior fixation T8-L4

Significant change (p<0.05) compared to

* Without posterior fixation
* Posterior fixation T8-L1
* Posterior fixation T8-L2
* Posterior fixation T8-L3

tested with Friedman’s ANOVA
+ Bonferroni-Dunn post-hoc correction
+ pairwise comparisons

Without posterior fixation
Posterior fixation T8-L1
Posterior fixation T8-L2
Posterior fixation T8-L3

tested with additional pairwise Friedman test
without post-hoc correction

IDP Right lateral bending

Group 1: Increasing fixation length in upward direction

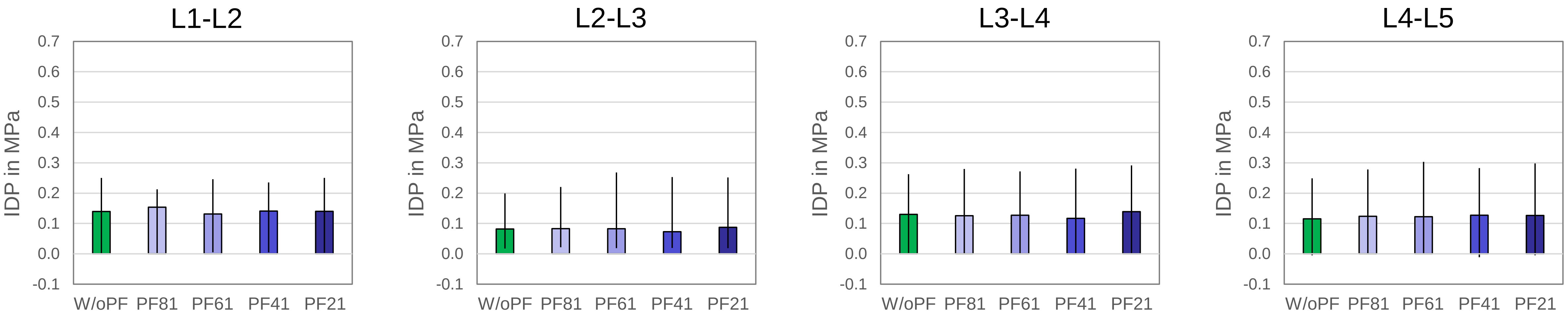

- W/oPF = Without posterior fixation
- PF81 = Posterior fixation T8-L1
- PF61 = Posterior fixation T6-L1
- PF41 = Posterior fixation T4-L1
- PF21 = Posterior fixation T2-L1

Significant change ($p<0.05$) compared to

* Without posterior fixation
* Posterior fixation T8-L1
* Posterior fixation T6-L1
* Posterior fixation T4-L1

tested with Friedman's ANOVA
+ Bonferroni-Dunn post-hoc correction
+ pairwise comparisons

Without posterior fixation
Posterior fixation T8-L1
Posterior fixation T6-L1
Posterior fixation T4-L1

tested with additional pairwise Friedman test
without post-hoc correction

IDP Right lateral bending

Group 2: Increasing fixation length in downward direction

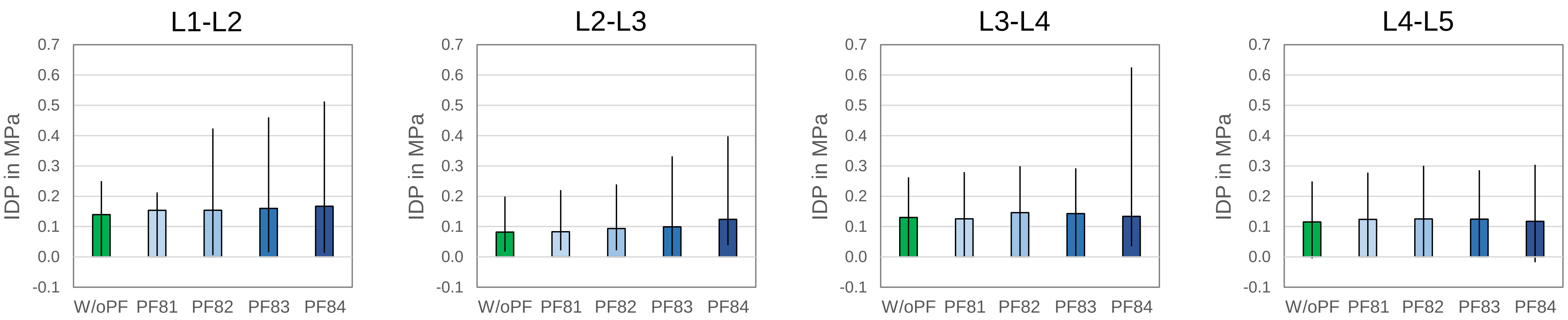

- W/oPF = Without posterior fixation
- PF81 = Posterior fixation T8-L1
- PF82 = Posterior fixation T8-L2
- PF83 = Posterior fixation T8-L3
- PF84 = Posterior fixation T8-L4

Significant change ($p<0.05$) compared to

*	Without posterior fixation	#	Without posterior fixation
*	Posterior fixation T8-L1	#	Posterior fixation T8-L1
*	Posterior fixation T8-L2	#	Posterior fixation T8-L2
*	Posterior fixation T8-L3	#	Posterior fixation T8-L3
	tested with Friedman's ANOVA<br>+ Bonferroni-Dunn post-hoc correction<br>+ pairwise comparisons		tested with additional pairwise Friedman test<br>without post-hoc correction

IDP Left axial rotation

Group 1: Increasing fixation length in upward direction

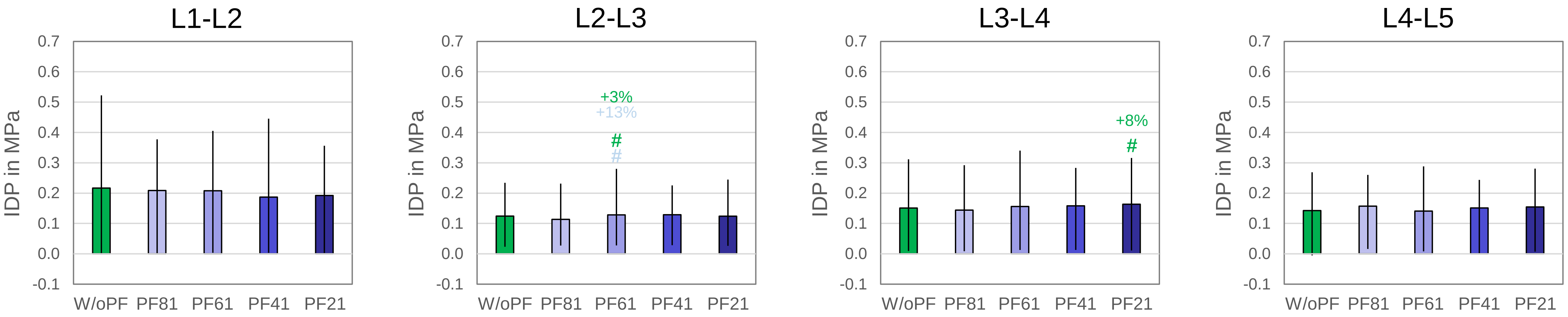

W/oPF = Without posterior fixation
PF81 = Posterior fixation T8-L1
PF61 = Posterior fixation T6-L1
PF41 = Posterior fixation T4-L1
PF21 = Posterior fixation T2-L1

Significant change (p<0.05) compared to

* Without posterior fixation	# Without posterior fixation
* Posterior fixation T8-L1	# Posterior fixation T8-L1
* Posterior fixation T6-L1	# Posterior fixation T6-L1
* Posterior fixation T4-L1	# Posterior fixation T4-L1
tested with Friedman’s ANOVA<br>+ Bonferroni-Dunn post-hoc correction<br>+ pairwise comparisons	tested with additional pairwise Friedman test<br>without post-hoc correction

IDP Left axial rotation

Group 2: Increasing fixation length in downward direction

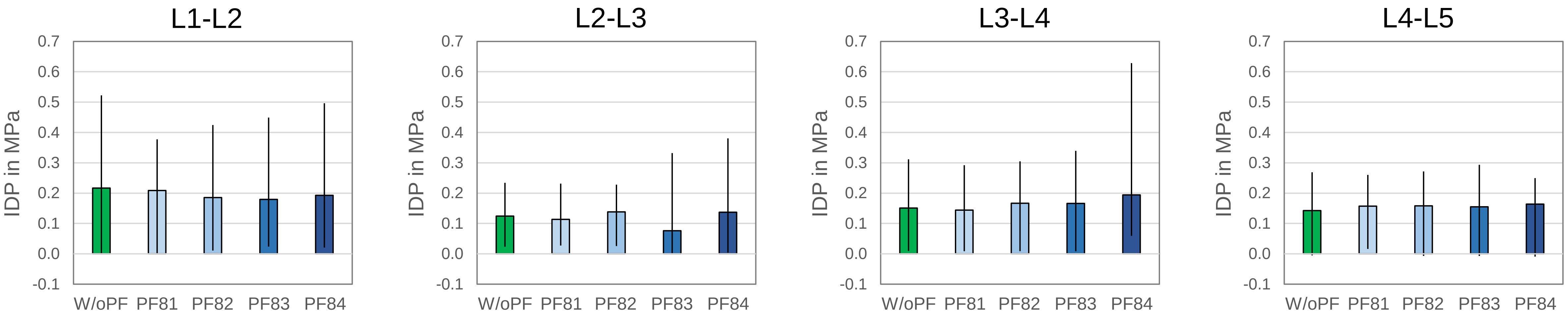

- W/oPF = Without posterior fixation
- PF81 = Posterior fixation T8-L1
- PF82 = Posterior fixation T8-L2
- PF83 = Posterior fixation T8-L3
- PF84 = Posterior fixation T8-L4

Significant change (p<0.05) compared to

* Without posterior fixation	# Without posterior fixation
* Posterior fixation T8-L1	# Posterior fixation T8-L1
* Posterior fixation T8-L2	# Posterior fixation T8-L2
* Posterior fixation T8-L3	# Posterior fixation T8-L3
tested with Friedman's ANOVA<br>+ Bonferroni-Dunn post-hoc correction<br>+ pairwise comparisons	tested with additional pairwise Friedman test<br>without post-hoc correction

IDP Right axial rotation

Group 1: Increasing fixation length in upward direction

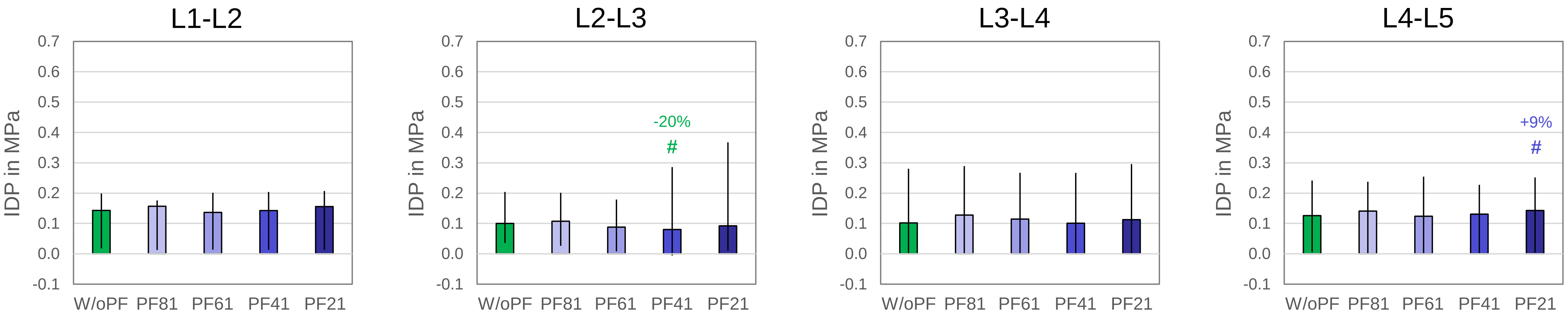

- W/oPF = Without posterior fixation
- PF81 = Posterior fixation T8-L1
- PF61 = Posterior fixation T6-L1
- PF41 = Posterior fixation T4-L1
- PF21 = Posterior fixation T2-L1

Significant change (p<0.05) compared to

* Without posterior fixation
* Posterior fixation T8-L1
* Posterior fixation T6-L1
* Posterior fixation T4-L1

tested with Friedman's ANOVA
+ Bonferroni-Dunn post-hoc correction
+ pairwise comparisons

Without posterior fixation
Posterior fixation T8-L1
Posterior fixation T6-L1
Posterior fixation T4-L1

tested with additional pairwise Friedman test
without post-hoc correction

IDP Right axial rotation

Group 2: Increasing fixation length in downward direction

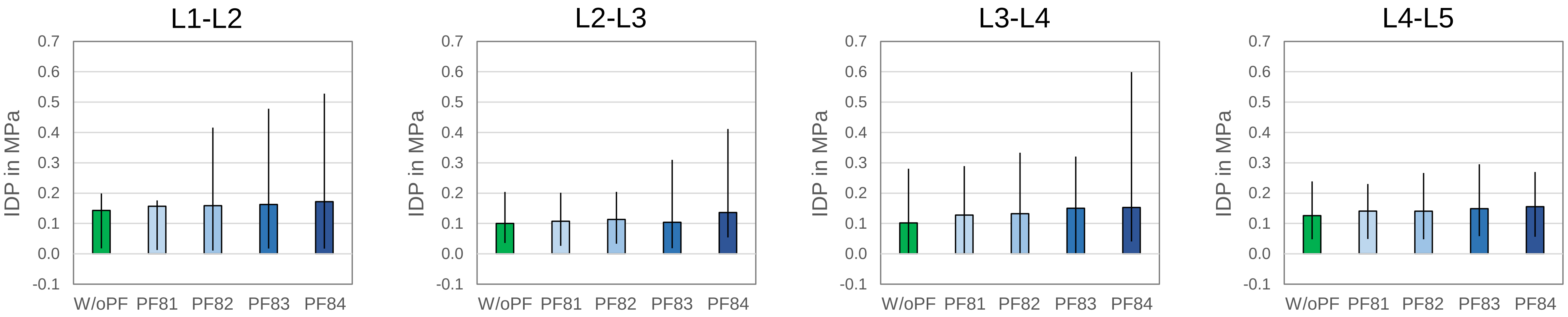

■ W/oPF = Without posterior fixation
■ PF81 = Posterior fixation T8-L1
■ PF82 = Posterior fixation T8-L2
■ PF83 = Posterior fixation T8-L3
■ PF84 = Posterior fixation T8-L4

Significant change (p<0.05) compared to

* Without posterior fixation
* Posterior fixation T8-L1
* Posterior fixation T8-L2
* Posterior fixation T8-L3

tested with Friedman’s ANOVA
+ Bonferroni-Dunn post-hoc correction
+ pairwise comparisons

Without posterior fixation
Posterior fixation T8-L1
Posterior fixation T8-L2
Posterior fixation T8-L3

tested with additional pairwise Friedman test
without post-hoc correction
